# Survey data on energy and fuel use of firms in economic zones in the Philippines

**DOI:** 10.1016/j.dib.2021.107637

**Published:** 2021-11-26

**Authors:** Majah-Leah Ravago, Raul Fabella, Karl Robert Jandoc, Renzi Frias, J. Kathleen Magadia

**Affiliations:** a Department of Economics, Ateneo de Manila University, Room 409 Leong Hall, Katipunan Avenue, Loyola Heights, 1108, Quezon City, Philippines; b School of Economics, University of the Philippines, Guerrero corner Osmeña Streets, Diliman, 1101, Quezon City, Philippines; c School of Statistics, University of the Philippines, T. M. Kalaw Street, Diliman, 1101, Quezon City, Philippines; d Gas Policy Development Project, UP Statistical Center Research Foundation, Inc., School of Statistics, University of the Philippines, Quirino Avenue Kalaw Street, Diliman, 1101, Quezon City, Philippines

**Keywords:** Fuel consumption, Natural gas, Fuel switch, Manufacturing and agro-industrial, Firm-level data, Philippines

## Abstract

The data describe characteristics, operations, utilities, and fuels used in the production of 115 manufacturing and agro-industrial firms in Philippine special economic zones. The data include information on the firm's production, sales, and schedules; electricity sources, requirements, and uses; the importance of various conventional fuels, and the firms’ fuel expenditure in their major production processes. The data also include their employee's aptitude, knowledge, considerations, and opinions on alternative fuels and primary energies, and experiences in using them. The data were gathered through a series of focus group discussions (FGDs) in June 2019 and an online survey conducted in August to September 2019. The data can be used in the analysis of energy consumption and expenditure of manufacturing and agro-industrial firms in the Philippines. The respondents’ knowledge of and perceptions toward adopting alternative fuels in their firms’ production processes are useful in the analysis of future energy demand.

## Specifications Table

**Table:** 

Subject	Energy (General), Economics
Specific subject area	Energy profile of manufacturing and agro-industrial firms in Philippine special economic zones
Type of data	Anonymized raw data (.csv) Data dictionary (.txt) Tables Figures
How data were acquired	Focus group discussions Online survey using subscription-based platform: SurveyMonkey (see https://www.surveymonkey.com)
Data format	Raw
Parameters for data collection	The data were collected from a series of focus group discussions and an online survey of manufacturing and agro-industrial firms in Philippine special economic zones.
Description of data collection	Focus group discussions (FGDs) were organized to pilot-test the online survey questionnaire in the Batangas and Laguna provinces. A total of 23 firms participated in the FGDs—15 of which responded to the questionnaire. The online survey was conducted for 30 days from August 8 to September 7, 2019. Participation in the survey was free and voluntary. Complete information of 100 unique firms was collected. The conduct of data collection adhered to the standard protocol of doing research and has satisfied the requirements of the Ateneo de Manila University Research Ethics Office (see https://www.ateneo.edu/research/university-research-ethics-office) for exemption from ethics review. Informed consent was obtained from the participants who have agreed to participate in the FGDs and online survey. Data gathered from the FGDs and the online survey were anonymized.
Data source location	The FGDs and online survey covered a sample of firms from the provinces of Batangas, Benguet, Bulacan, Cavite, Cebu, Laguna, and Pampanga, and as well as the Metro Manila region.
Data accessibility	Ravago, Majah-Leah; Fabella, Raul; Jandoc, Karl Robert; Frias, Renzi; Magadia, J. Kathleen (2021), “Survey Data on Energy and Fuel Use of Firms in Economic Zones in the Philippines”, Mendeley Data, V2, doi:10.17632/88t45xbn59.2[1] Instructions for accessing these data: Standard access via Mendeley **Supplementary appendices** Appendix 1 DIB Energy Ravago et al 2021_Data.csv Appendix 2 DIB Energy Ravago et al 2021_Dictionary.txt Appendix 3 DIB Energy Ravago et al 2021_FGD questionnaire.pdf Appendix 4 DIB Energy Ravago et al 2021_Survey questionaire.pdf Appendix 5 DIB Energy Ravago et al 2021_General results.pdf
Related research article	Ravago, Majah-Leah; Fabella, Raul; Jandoc, Karl Robert; Frias, Renzi; Magadia, J. Kathleen, “Gauging the Market Potential for Natural Gas among Philippine Manufacturing Firms”, Energy, 237, 121563, https://doi.org/10.1016/j.energy.2021.121563 [2]

## Value of the Data

• The data are useful in analysing the energy consumption and expenditure behavior of large and intensive users such as manufacturing and agro-industrial firms in the Philippines.
• The data are useful in evaluating how knowledge and perceptions toward alternative fuel can shape future demand.
• The data are useful for policymakers and energy sector regulators in crafting policies and guidelines on energy related concerns such as energy efficiency, transition to clean energy, among others.
• The data can provide insights on the likelihood of switching from conventional to alternative fuels in major manufacturing and industrial production processes.
• The data may be used by researchers to develop longitudinal studies that could capture how technological changes influence the demand and usage of various fuels.
• The data offer the potential to scale the size of data collection to include other manufacturing and industrial firms outside the special economic zones.

## 1 Data Description

The data were gathered from firms operating inside the manufacturing and agro-industrial special economic zones in the Philippines through a series of focus group discussions (FGDs) and an online survey. The collected data include information on the firm's profile, production schedule and operation, utilities, energy efficiency initiatives, fuels used in production, aptitude on alternative fuels and primary energies, and business considerations. A total of 115 firms coming from 8 provinces participated in the online survey.

Supplementary appendices provide the full data set and survey instruments. Supplementary Appendix 1 is the CSV file containing the anonymized raw survey data. Supplementary Appendix 2 is the data dictionary (in .txt format), which includes the questions, response options, and variable names used in data preparation and tabulation. Supplementary Appendices 3 and 4 are the questionnaires. Supplementary Appendix 5 is the summary of the results.

### 1.1 Reading the data

The raw data are available in Supplementary Appendix 1 (in .csv format). Each row in the data set represents one set of responses of each unique firm. A unique firm identification number was generated to aid the data users. The firm ID (“*locatorid”* variable) contains nine digits. The first two digits represent the province. The next two digits represent the city or municipality, followed by another two digits for the economic zone. The last three digits represent the firm.

The data set only includes the non-personal and non-identifiable information gathered from the survey. Information such as firm and respondent names and contact information were permanently deleted in the database in compliance with the data privacy protection law and standard research ethics protocols.

The data set is organized the same way as the survey questionnaire (i.e., section and question numbers are the same). Each column in the data set is headed by the variable name. The variable names start with the section number and question number followed by keywords for the summary of question, unit of measure, and response option. For example, variable “*sIq1_ecozone*” is the corresponding variable for Section I Question 1 on ecozone. Another example: “*sIVq80_diesel_cons_lit_transpo*” is the corresponding variable for Section IV Question 80 on diesel consumption for transportation and has a unit of a liter.

Useful in reading the data set is the Supplementary Appendix 2 or the data dictionary (in .txt format). Basic information about each variable is described in the dictionary. Each line or entry in the dictionary contains three key information about each variable – data type, variable name, and variable label. The first part of each entry, data type, tells whether the variable assumes numeric or string data. Numeric data are represented in the data dictionary as either byte, int, long, float, or double. String data, on the other hand, are represented as “str” followed by a number which represents the maximum number of characters of the variable.

For questions that asked for multiple responses, one variable is generated for each response option. For example, Section III Question 33 asked the types of fuel used for self-generation of power. The variables associated with this question are “*sIIIq33_selfgen_biodie*”, “*sIIIq33_selfgen_bunker*”, etc. For questions that required only one response, the actual response is recorded as it is.

### 1.2 General results

The general results of the survey are presented as a supplementary document of this data article (see Supplementary Appendix 5). The organization of the results in Supplementary Appendix 5 follows the sections of the questionnaire as outlined in Table 4. The following provides brief descriptions of the information gathered and some selected results as provided in Supplementary Appendix 5.

#### 1.2.1 General information

Section I of Supplementary Appendix 5 provides a summary of basic ecozone and firm information such as geographic location, size, and book value. This subsection covers Questions 1 to 9 of the survey questionnaire.

Out of the 115 firm-respondents, 64.35% came from the Laguna province and 10.43% from the Batangas province. The other respondents were from Cavite (8.70%), Cebu (7.83%), Pampanga (6.09%), Metro Manila (0.87%), Bulacan (0.87%), and Benguet (0.87%).

#### 1.2.2 Production schedule and operation

Section II of Supplementary Appendix 5 presents information on the production, sales, peak and low month schedules, and operations of the firms in the sample. This subsection covers Questions 10 to 27 of the survey questionnaire.

#### 1.2.3 Utilities and energy efficiency

Section III of Supplementary Appendix 5 includes a discussion on electricity sources, requirements, uses, and considerations of the firms. Consumption and expenditure on electricity and water and energy conservation practices are also presented. This subsection covers Questions 28 to 56 of the survey questionnaire.

In Table 1, 81 firms in the sample source electricity either from the Manila Electric Company (Meralco) or any electric cooperative, the former being the largest power distributor in the Philippines. On the other hand, 21 firms source their electricity from retail electricity suppliers (RES). This is followed by generation firms directly connected to the National Grid Corporation of the Philippines (NGCP) (6), power plants inside the economic zone (5), other private firms and PEZA (5), and self-generation (2).

**Table 1:** Number of firms by electricity source.

Source	N
Power plant inside ecozone	5
Meralco or electric cooperative	81
Retail electricity supplier (RES)	21
Direct from generation company (directly connected to NGCP)	6
Self-generation	2
Other (private electric companies, PEZA, etc.)	5

Note: Five of the sampled firms source electricity from more than one source.

#### 1.2.4 Fuels used in production

Section IV of Supplementary Appendix 5 examines the firms’ production fuel mix, importance, use, consumption, and expenditure on the different types of fuel in main production processes. This subsection covers Questions 57 to 130. The definitions of each fuel and each production process are also available in Supplementary Appendix 5.

Out of the 115 respondents, only 56 firms use any fuel aside from electricity in their main production processes. 39.29% of the respondents use diesel in their production processes. These production processes include processes with a heating component such as fabrication, heat treatment, die casting or wire bonding, and without heating components such as forklift operation, transportation and logistics, stamping, and engine loading. On LPG use, 39.29% also uses it for their heating and non-heating production components. Gasoline, on the other hand, is used by 17.86% of the fuel-using firms, followed by kerosene (8.93%), natural gas (5.36%), and propane. (3.57%). The complete data on which production process uses each fuel can be found in the accompanying Supplementary Appendix 5.

**Fig 1:**
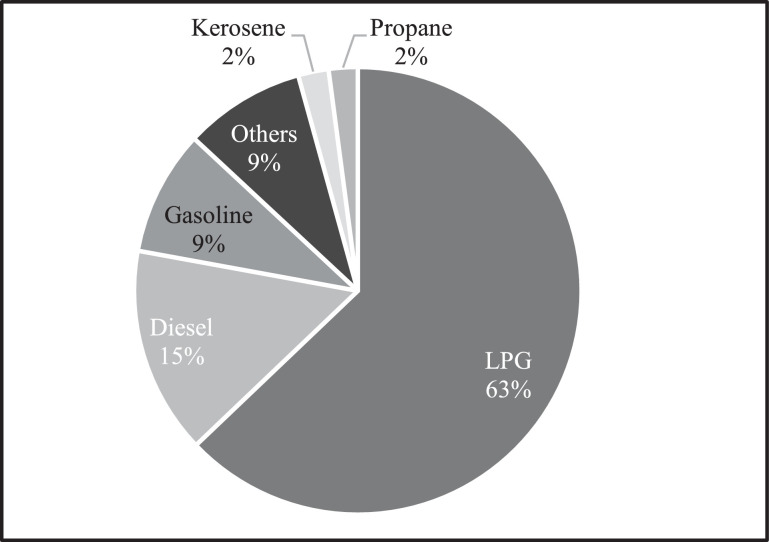
Expenditure share per fuel.

Notes: In the survey, LPG is defined as a combination of propane and butane. Biodiesel, bunker, and coal were not included in the figure. The “Others” category includes electricity, hydrogen, biomass, Thuban, nitrogen, oxygen, argon, helium, ricehull, hydraulic oil, and engine oil. Since natural gas is not yet commercially available in the Philippines, further verification was conducted to firms that said they are using natural gas in their production processes. After verification, the firms clarified that the natural gas that they are referring to is nitrogen, oxygen, argon, and helium gases, so it was reclassified to the other fuel category.

Figure 1 below shows that the majority of the firms in the sample spend the most on LPG as a production fuel. 63% of the fuel expenditures of firms is LPG. This is followed by diesel at 15%, gasoline at 9%, other fuels (9%), kerosene (2%), and propane (2%).

#### 1.2.5 Aptitude on alternative fuels and primary energies

Section V of Supplementary Appendix 5 (aptitude on alternative fuels and primary energies) covers Questions 131 to 169 of the questionnaire. This subsection section presents information on knowledge, considerations, and opinions on alternative fuels (natural gas), and primary energies (solar and wind), and their experiences in using them. Natural gas in this subsection is defined as a fossil energy source that formed deep beneath the earth's surface and is largely composed of methane [3]. On the other hand, solar energy is energy from the sun that is converted to thermal or electrical energy [4], while wind energy is from the airflows that are also converted to the same energies [5].

Table 2 shows that most respondents have limited knowledge of natural gas and wind as fuel or fuel sources and moderate knowledge of solar as fuel or fuel source. Of the total, 44.35% of the respondent firms have limited knowledge, while 29.57% have moderate knowledge of natural gas as fuel. In terms of wind as an energy source, 36.52% have limited knowledge, while 26.09% have moderate knowledge. On the other hand, 38.26% of the respondents have moderate knowledge, while 26.09% have an above moderate level of knowledge with solar as a primary energy source.

**Table 2:** Percentage of respondents with knowledge of natural gas, solar, and wind.

	Natural Gas	Solar	Wind
1 (Limited)	44.35%	13.91%	36.52%
2	14.78%	13.04%	18.26%
3	29.57%	38.26%	26.09%
4	9.57%	26.09%	15.65%
5 (Advanced)	1.74%	8.70%	3.48%
Weighted Mean	2.10	3.03	2.31
N	115	115	115

Even though the firms have limited to moderate knowledge on natural gas, solar, and wind as fuel, the majority of them still thinks that the fuel sources are safe to use in the production process. 57.37% of the respondents perceive natural gas as a safe production fuel, 86.09% for solar, and 58.26% for wind. The complete results on this can be found in Supplementary Appendix 5.

#### 1.2.6 Business considerations

Section VI of Supplementary Appendix 5 covers respondents considerations in business expansions.

## 2 Experimental design, materials, and methods

Special Economic Zones (SEZs) are characterized as distinct areas where firms can benefit from lower export fees, taxes, import tariffs, and less bureaucracy, inspections, and paperwork [6]. By providing such preferential policies, SEZs can provide an attractive environment for foreign direct investments. It also paves way for the adoption of new technologies and upgrading of skills. These are very important factors particularly to developing economies that aim to diversify their production base into manufacturing.

Due to its specialized facilities and technology, the energy demand and intensity of manufacturing and agro-industrial in SEZs are recognizably much greater than their counterparts in non-SEZs. A good number of firms in the SEZs use heaters, boilers, and turbines as part of their production process. They use the more expensive diesel, liquified petroleum gas (LPG), coal, among others as their fuel. Taken together, the primary fuel requirement of these firms can be quite substantial.

The data collected on the energy and fuel usage from firms of SEZ are useful in analysing the energy consumption and expenditure of manufacturing and agro-industrial firms in the Philippines. The data are useful in evaluating the shape of future demand.

Our primary survey aims to characterize the profile of the firms within manufacturing and agro-industrial ecozones. It asked for information on the firm's profile, production schedule and operation, utilities, energy efficiency initiatives, fuels used in production, aptitude on alternative fuels and primary energies, and business considerations. It also collected data on the likelihood of adopting alternative fuels and primary energies such as natural gas, solar, and wind in their existing production processes.

### 2.1 Scope and coverage area

The respondents of the primary survey are firms within ecozones classified as manufacturing and agro-industrial by the Philippine Economic Zone Authority (PEZA). Table 3 provides the list of sixty-one (61) public and private ecozones that house 1,613 operating firms invited to answer the survey. The manufacturing and agro-industrial zones identified were based on the February 2018 list of firms available online [7].

**Table 3:** List of ecozones invited to participate in the survey.

1. Baguio City Economic Zone* 2. Cavite Economic Zone * 3. Mactan Economic Zone* 4. Pampanga Economic Zone * 5. AG&P SEZ 6. Agrotex Gensan Economic Zone 7. Agus Industrial Estate 8. AJMR Agro-Industrial Economic Zone 9. Angeles Industrial Park 10. Asahi Glass SEZ 11. Balo-I Agro-Industrial Economic Zone 12. Calamba Premiere Industrial Park 13. Carmelray Industrial Park 14. Carmelray Industrial Park II 15. Cavite Biofuels Ecozone 16. Cavite Technopark 17. Cebu Light Industrial and Science Park 18. Carmen Cebu Gum Industrial 19. Cocochem Agro-Industrial Park 20. DADC Economic Zone 21. Daiichi Industrial Park 22. First Cavite Industrial Estate 23. First Industrial Township 24. First Philippine Industrial Park 25. Gensan Economic Zone 26. Golden Gate Business Park 27. Golden Mile Business Park 28. Greenfield Automotive Park 29. Hermosa Ecozone Industrial Park 30. Keppel Phils. Marine SEZ 31. Laguna International Industrial Park	32. Laguna Technopark, Inc. 33. Leyte Industrial Development Estate 34. Light Industry & Science Park I 35. Light Industry & Science Park II 36. Light Industry & Science Park III & IV 37. Lima Technology Center 38. Luisita Industrial Park 39. Mactan Economic Zone II 40. MRI Special Economic Zone 41. New Jubilee Agro-Industrial Economic Zone 42. Palayan City Government Center and Central Business Hub 43. Pangasinan Industrial Park II 44. Phil. Packaging Agricultural EPZ 45. Phividec Industrial Estate-Economic Zone 46. Plastic Processing Center SEZ 47. Samar Agro-Industrial Economic Zone 48. San Carlos Economic Zone 49. Santa Maria Industrial Park 50. Sarangani Economic Development Zone 51. SRC Allah Valley Economic Development Zone 52. SRC Calumpang Economic Development Zone 53. Subic Shipyard Special Economic Zone 54. Suntrust Ecotown Tanza 55. Tabangao Special Economic Zone 56. Taganito Special Economic Zone 57. TECO-Special Economic Zone 58. Toyota Sta. Rosa (Laguna) Special Zone 59. Victoria Wave Special Zone 60. West Cebu Industrial Park 61. YTMI Realty Special Economic Zone

Note: With asterisks are public ecozones; the others are private. Public ecozones are owned by PEZA. Private ecozones are owned by private developers. Both public and private ecozones in the list are registered with PEZA.

### 2.2 Design of survey instrument

The survey questionnaire covers six sections as listed in Table 4. We asked for general information about the firm, production and operation schedule, fuels used in production, employees' aptitude on alternative fuels, and other information including the factors that affect business expansion. A copy of the survey questionnaire is provided in Supplementary Appendix 4.

**Table 4:** Coverage of survey questionnaire.

Section	Coverage
—	About the survey; administrator contact information; privacy notice including statements on personal data collection, methods of processing, purposes of collection, information on personal information controller, data sharing and disclosure to third parties, confidentiality, and contact information; general instructions; overview of sections
I. General information	Ecozone and firm's information, personnel, book value
II. Production schedule and operation	Production, sales, peak and low month schedule and operation
III. Utilities	Electricity sources, requirements, uses, and considerations; electricity and water consumption and expenditure; energy conservation
IV. Fuels used in production	Importance, uses, consumption, and expenditure on different types of fuel in main production processes
V. Aptitude on alternative fuels and primary energies	Knowledge, considerations, and opinions on alternative fuels and primary energies, and experiences in using them
VI. Other questions	Business expansion considerations
VII. Respondent information	Primary and secondary respondent information
—	Project information

Note: Accessible data in Supplementary Appendix 1 do not contain identifiable information.

The questionnaire was administered using the subscription-based survey platform called SurveyMonkey.^1^ PEZA's assistance was instrumental in the data gathering, endorsing the study through a memorandum sent to all concerned economic zones, which also contained the survey link. The survey link and password were forwarded to the respective zone managers of each of the 61 ecozones. The survey was open for 30 days from August 8 to September 7, 2019.

### 2.3 Pilot testing via focus group discussion

Focus group discussions (FGDs) were organized prior to the dissemination and conduct of the primary online survey to pilot-test the instrument and gather feedback from the firms on the clarity and ease of understanding of the questionnaire and to improve the accuracy of the response categories. The FGDs were also conducted to seek clarification on the firms’ responses and gain a better understanding of their production processes and finances. The insights gathered from the FGDs were used to improve the accuracy and comprehensiveness of the survey instrument. The conduct of the FGDs was concentrated in ecozones located in Batangas and Laguna.

During the FGDs, participants were consulted regarding select suvery questions, specifically those with response options in order to verify clarity and ease of understanding. Level of difficulty in answering the survey; officials who they thought should accomplish the questionnaire; and suggestions to improve the instrument were also asked among the participants. The questions were designed to fit 1.5 hours of FGD sessions.

Five manufacturing ecozones in Laguna and Batangas were selected for the FGDs. The ecozones were chosen based on proximity with each other to facilitate efficiency. Firms within the ecozones with the greatest number of employees were chosen to participate in the FGDs and pilot-test of questionnaire as it suggests sizeable production and operations. Employees who accomplished the survey were the ones requested to represent the firms during the FGD.

### 2.4 Sampling protocol

Adhering to the standard ethics protocol in conducting research and keeping the survey optional, we employed a simple random sampling procedure [8] targeted to manufacturing and agro-industrial SEZs. Our survey was sent to 61 manufacturing and agro-industrial SEZs with a total of 1,613 operating firms [7]. Given this population, a 95 per cent confidence level, and a 10 per cent margin of error, our ideal sample size is 91 firms.

After the survey period, a total of 115 unique firms responded to the survey – 100 from the primary survey and 15 from the FGD survey. This sample gives us a 9 per cent margin of error. These firms are from 24 ecozones out of the 61 targeted ecozones. The 115 total respondents were unique and submitted complete responses. A response was classified as complete only when the respondent was able to answer the entire questionnaire. In addition, only one response per firm was considered valid. All other responses from the same firm were classified as duplicates. Firms with multiple responses were followed up to verify which among their responses should be considered. Respondents representing the firms are directors, supervisors, managers, or officers for finance and accounting; sales and marketing; human resource; pollution control and environment; production and operations; or facilities, equipment, and utilities.

Published research on the conduct of surveys in organizations and workplaces typically has 15-60 participants [9]. Our sample of 115 firms is considered a successful return, given that the survey is voluntary. Our sample is larger than the 82 firms surveyed by the Japan International Cooperation Agency (JICA) in 2011 [10], although the JICA study covers only firms in economic zones along the proposed Batangas-Manila (BatMan 1) natural gas pipeline (i.e., Batangas and Laguna areas only).

### 2.5 Imputation

After the FGD, some value ranges were revised to account for the feedback obtained from the FGD. For primary survey questions that required exact numbers, values were imputed from the FGD responses by averaging the lowest and the highest value in the range option.

For FGD responses that do not fall within any of the new ranges in the survey, the second-lowest options in the upper brackets were chosen since the FGD participants were chosen based on firm size. For FGD responses that fall within more than one range, the average of the original value range was used to assign the new value range.

Meanwhile, the personnel categories “administrative staff” and “other personnel” in the FGD survey were aggregated as “administrative and support” in the primary survey, while “technical staff” and “production staff” were aggregated as “technical and production.”

## Ethics statement

The proposal to conduct the survey has been examined and validated exempt from review by the Ateneo de Manila University Research Ethics Committee.^2^ As such, the conduct of the survey fulfilled the technical requirements necessary to demonstrate the use of the ethical procedure in research involving human respondents. Implicit informed consent has been obtained from the participants because they have agreed to be interviewed. They have also been appropriately informed that personal information is treated with the utmost confidentiality. No identifiable information appears in the data gathered from the survey.

## CRediT author statement

**MVRavago** conceptualized the project, in charge of the overall supervision of the project; collaborated with the other authors in the design and creation of the survey; supervised the data collection process; was involved in the focus group discussion; was involved and contributed to the analysis, writing, and review of the manuscript.

**RVFabella** collaborated with the other authors in the design and creation of the survey; was involved in the focus group discussion; was involved and contributed to the analysis, writing, and review of the manuscript.

**KLJandoc** collaborated with the other authors in the design and creation of the survey; was involved in the focus group discussion; was involved and contributed to the analysis, writing, and review of the manuscript.

**RGFrias** collaborated with the other authors in the creation of the survey; was responsible for translating the survey instrument using the online platform; was involved in the data collection process and data processing; was involved and contributed to the analysis, writing, and review of the manuscript.

**JPMagadia** collaborated with the other authors in the creation of the survey; was involved in the data collection process and coordination with the respondents; was involved and contributed to the writing, and review of the manuscript.

## Declaration of Competing Interest

The authors declare that they have no known competing financial interests or personal relationships which have, or could be perceived to have, influenced the work reported in this article.

## Appendix Supplementary materials

Appendix 1 Anonymized raw data (DIB Energy Ravago et al 2021_Data.csv) Appendix 2 Data dictionary (DIB Energy Ravago et al 2021_Dictionary.txt) Appendix 3 FGD Survey questionnaire (DIB Energy Ravago et al 2021_FGD questionnaire.pdf) Appendix 4 Survey questionnaire (DIB Energy Ravago et al 2021_Survey questionaire.pdf) Appendix 5 General results from the survey (DIB Energy Ravago et al 2021_General results.pdf) Image, application 1

## Footnotes

1 See https://www.surveymonkey.com.

2 See https://www.ateneo.edu/research/university-research-ethics-office.
