# Supplementary material for: Survey data on energy and fuel use of firms in economic zones in the Philippines
Source: Data Brief. 2021 Nov 26;39:107637. doi: 10.1016/j.dib.2021.107637 (PMC8645435; doi:10.1016/j.dib.2021.107637)
Supplement: Supplementary file 1 — Appendix 1 Anonymized raw data (DIB Energy Ravago et al 2021_Data.csv) Appendix 2 Data dictionary (DIB Energy Ravago et al 2021_Dictionary.txt) Appendix 3 FGD Survey questionnaire (DIB Energy Ravago et al 2021_FGD questionnaire.pdf) Appendix 4 Survey questionnaire (DIB Energy Ravago et al 2021_Survey questionaire.pdf) Appendix 5 General results from the survey (DIB Energy Ravago et al 2021_General results.pdf) [file mmc1.zip › Supplementary Appendix 3 DIB Energy Ravago et al 2021_ FGD Questionnaire .pdf]

Survey Data on Energy and Fuel Use of Firms in Economic Zones in the Philippines
Supplementary Appendix 3: FGD Survey questionnaire

Authors

Majah-Leah Ravago[1], Raul Fabella[2], Karl Robert Jandoc[2], Renzi Frias[3,4], J. Kathleen Magadia[4]

Affiliations

1. Department of Economics, Ateneo de Manila University, Room 409 Leong Hall, Katipunan Avenue, Loyola Heights, 1108, Quezon City, Philippines
2. School of Economics, University of the Philippines, Guerrero corner Osmeña Streets, Diliman, 1101, Quezon City, Philippines
3. School of Statistics, University of the Philippines, T. M. Kalaw Street, Diliman, 1101, Quezon City, Philippines
4. Gas Policy Development Project, UP Statistical Center Research Foundation, Inc., School of Statistics, University of the Philippines, Quirino Avenue Kalaw Street, Diliman, 1101, Quezon City, Philippines

Corresponding author

Majah-Leah Ravago (mravago@ateneo.edu)

	SECTION	DESCRIPTION	PAGE
	—	About the survey; statement on data privacy and accuracy; general instructions; overview of sections	1 - 3
**I**	**General Information**	Ecozone, firm, and primary respondent information; personnel; book value	4 - 8
**II**	**Production Schedule and Operation**	Production sales; peak and low month schedule and operation	9 - 18
**III**	**Utilities**	Electricity sources, requirements, uses, and considerations; electricity and water consumption and expenditure; energy conservation	19 - 32
**IV**	**Fuels Used in Production**	Importance, use, consumption, and expenditure on different types of fuel (biodiesel, bunker, coal, diesel, gasoline, kerosene, LPG, natural gas, propane, other fuels) in main production processes	33 - 126
**V**	**Aptitude on Alternative Fuels and Primary Energies**	Knowledge, considerations, and opinions on alternative fuels and primary energies (natural gas, solar, wind), and experiences in using them	127 - 145
**VI**	**Profile of Other Respondents**	Information on other respondents	146

Survey for Philippine Special Economic Zones

About the survey

On average, this survey will take **30 minutes** to finish.

This research survey is being undertaken by the UP Statistical Center Research Foundation, Inc. - Gas Policy Development Project (UPSCRFI-GPDP), the Philippine Economic Zone Authority (PEZA), and the Department of Energy (DOE) to establish baseline information, and gather market profile of Philippine Special Economic Zones.

Data privacy and accuracy

Your responses are very important to ensure the accuracy of the research, and will be treated confidentially. Data gathered will only be used by the GPDP, PEZA, and/or DOE, and would be reported in averages.

If you have any question or encounter any problem during the survey, please contact **infogpdp.ph@gmail.com**.

Instructions

This survey will ask for details on **production processes and finances** which may need the assistance of an official or personnel knowledgeable on it. This survey may also be answered by **more than one (1) official** of the company if needed. Best persons to answer are **operations and finance managers/officers**.

Kindly prepare your **water and electricity bills for the past 3 months**, and use them as references for the questions on utility consumption and expenditure.

A **calculator** may also be needed to convert values for questions on fuel consumption and expenditure.

Questions will generally ask for the **average values** only.

This survey may only be answered **once from the same device**. You may change your answers, and go back to particular pages before clicking the 'Submit' button at the end of the survey.

Sections

I. General information
II. Production schedule and operation
III. Utilities
IV. Fuels used in production
V. Aptitude on alternative fuels and primary energies
VI. Profile of other respondents

I. General Information

In this section, you will be asked about the company information and primary respondent details.

I. General Information

* 1. What is the name of your ecozone?

- ◯ Calamba Premiere International Park
- ◯ First Philippine Industry Park
- ◯ Laguna Technopark
- ◯ Light Industry and Science Park I
- ◯ Lima Technology Center
- ◯ Other (please specify)

* 2. Company information

Name of company

Street address

City or municipality

Province

ZIP code

Contact person or name of primary respondent

Position

Telephone number

Email address

* 3. How many personnel does your company have?

	1 to 250	251 to 500	501 to 750	751 to 1,000	1,001 and above	Not applicable
Administrative staff (e.g. secretaries, clerks, etc.)	◯	◯	◯	◯	◯	◯
Production staff	◯	◯	◯	◯	◯	◯
Technical staff (e.g. engineers, accountants, etc.)	◯	◯	◯	◯	◯	◯
Other personnel	◯	◯	◯	◯	◯	◯

I. General Information

* 4. How much is your total expense (**IN PESOS**) for the salary of all the staff? Disaggregate your answers per type of staff.

	1 to 250,000	250,001 to 500,000	500,001 to 750,000	750,001 to 1,000,000	1,000,001 and above
Administrative staff (e.g. secretaries, clerks, etc.)	◯	◯	◯	◯	◯
Production staff	◯	◯	◯	◯	◯
Technical staff (e.g. engineers, accountants, etc.)	◯	◯	◯	◯	◯
Other personnel	◯	◯	◯	◯	◯

I. General Information

* 5. What is the estimated book value (**IN PESOS**) of your tangible fixed assets as of December 2018?

Tangible fixed assets *refer to physical assets required and for use of the company, and is expected to have a productive life of more than one year. They include land; buildings, other structure and land improvements; transport equipment such as cars, trucks, aircrafts, and ships; machinery and equipment; valuables such as paintings and sculptures; and other tangible fixed assets such as fixtures and furnitures.*

Book value *refers to the initial or acquisition cost of tangible fixed assets less accumulated depreciation charges.*

- ◯ 1 to 200,000,000
- ◯ 200,000,001 to 400,000,000
- ◯ 400,000,001 to 600,000,000
- ◯ 600,000,001 to 800,000,000
- ◯ 800,000,001 to 1,000,000,000
- ◯ 1,000,000,001 and above

II. Production Schedule and Operation

In this section, you will be asked about the production schedules and operations.

II. Production Schedule and Operation

* 6. Are your products sold domestically or exported?

- ◯ Sold domestically
- ◯ Exported
- ◯ Both

* 7. How much was your annual production sales (**IN PESOS**) in **2018**?

- ◯ 1 to 200,000,000
- ◯ 200,000,001 to 400,000,000
- ◯ 400,000,001 to 600,000,000
- ◯ 600,000,001 to 1,000,000,000
- ◯ 1,000,000,001 and above

II. Production Schedule and Operation

* 8. When was your **PEAK MONTH** (i.e. month when production was highest) in **2018?**

- ◯ January
- ◯ February
- ◯ March
- ◯ April
- ◯ May
- ◯ June
- ◯ July
- ◯ August
- ◯ September
- ◯ October
- ◯ November
- ◯ December

* 9. During your **PEAK MONTH**, how many **DAYS** did your **MAIN PRODUCTION** equipment and facilities operate?

- ◯ Less than 20
- ◯ 20 to 23
- ◯ 24 to 27
- ◯ 28 to 31

* 10. During your **PEAK MONTH**, how many **HOURS PER DAY** did your **MAIN PRODUCTION** facilities and equipment operate?

- ◯ Less than 8
- ◯ 8 to 9
- ◯ 10 to 11
- ◯ 12 to 13
- ◯ More than 13

II. Production Schedule and Operation

* 11. During your **PEAK MONTH**, how many **DAYS** did your **AUXILIARY** equipment and facilities (e.g. temperature/climate controllers, air conditioners, refrigerators, etc.) operate?

- ◯ Less than 20
- ◯ 20 to 23
- ◯ 24 to 27
- ◯ 28 to 31
- ◯ We do not have any auxiliary equipment and facilities.

II. Production Schedule and Operation

* 12. During your **PEAK MONTH**, how many **HOURS PER DAY** did your **AUXILIARY** equipment and facilities (e.g. temperature/climate controllers, air conditioners, refrigerators, etc.) operate?

- ◯ Less than 8
- ◯ 8 to 9
- ◯ 10 to 11
- ◯ 12 to 13
- ◯ More than 13

II. Production Schedule and Operation

* 13. When was your **LOW MONTH** (i.e. month when production was lowest) in **2018?**

- ◯ January
- ◯ February
- ◯ March
- ◯ April
- ◯ May
- ◯ June
- ◯ July
- ◯ August
- ◯ September
- ◯ October
- ◯ November
- ◯ December

* 14. During you **LOW MONTH**, how many **DAYS** did your **MAIN PRODUCTION** equipment and facilities operate?

- ◯ Less than 15
- ◯ 15 to 18
- ◯ 19 to 22
- ◯ 23 to 26
- ◯ More than 26

* 15. During your **LOW MONTH**, how many **HOURS PER DAY** did your **MAIN PRODUCTION** facilities and equipment operate?

- ◯ Less than 5
- ◯ 5 to 6
- ◯ 7 to 8
- ◯ 9 to 10
- ◯ More than 10

II. Production Schedule and Operation

* 16. During your **LOW MONTH**, how many **DAYS** did your **AUXILIARY** equipment and facilities (e.g. temperature/climate controllers, air conditioners, refrigerators, etc.) operate?

- ◯ Less than 15
- ◯ 15 to 18
- ◯ 19 to 22
- ◯ 23 to 26
- ◯ More than 26
- ◯ We do not have any auxiliary equipment and facilities.

II. Production Schedule and Operation

* 17. During your **LOW MONTH**, how many **HOURS PER DAY** did your **AUXILIARY** equipment and facilities (e.g. temperature/climate controllers, air conditioners, refrigerators, etc.) operate?

- ◯ Less than 5
- ◯ 5 to 6
- ◯ 7 to 8
- ◯ 9 to 10
- ◯ More than 10

III. Utilities

In this section, you will be asked about your electricity sources, requirements, uses, and conservation measures. You will also be asked about your water and electricity consumption and expenditure for the past 3 months.

III. Utilities

* 18. What are your **MAIN SOURCES** of electricity? Please check all that apply.

- [ ] Power plant inside ecozone
- [ ] Meralco or electric cooperative
- [ ] Retail electricity supplier
- [ ] Direct from NGCP
- [ ] Self-generation
- [ ] Other (please specify)

III. Utilities

* 19. How much of your electricity requirement (**IN %**) is supplied by each source? Sum must be equal to 100.

Power plant inside ecozone

Meralco or electric cooperative

Retail electricity supplier

Direct from NGCP

Self-generation

[Insert text from Other]

III. Utilities

20. If you chose 'self-generation' in the previous question, which fuel or primary energy do you use? Please check all that apply.

- [ ] Biodiesel
- [ ] Bunker
- [ ] Coal
- [ ] Diesel
- [ ] Gasoline
- [ ] Kerosene
- [ ] LPG
- [ ] Natural gas
- [ ] Propane
- [ ] Solar
- [ ] Wind
- [ ] Other (please specify)

III. Utilities

* 21. Do you have back-up generation unit/s?

○ Yes

○ No

III. Utilities

* 22. Which fuel or primary energy do you use for back-up power generation? Please check all that apply.

- [ ] Biodiesel
- [ ] Bunker
- [ ] Coal
- [ ] Diesel
- [ ] Gasoline
- [ ] Kerosene
- [ ] LPG
- [ ] Natural gas
- [ ] Propane
- [ ] Solar
- [ ] Wind
- [ ] Other (please specify)

III. Utilities

* 23. Which processes involved in your main production use **ELECTRICITY**? Please check all that apply.

- [ ] Air conditioning
- [ ] Air/gas mixing (e.g. Selas mixing, etc.)
- [ ] Baking
- [ ] Boiler operation (e.g. for steam generation, etc.)
- [ ] Burning
- [ ] Curing (e.g. oven curing, powder paint curing, etc.)
- [ ] Die casting
- [ ] Drying or annealing (e.g. oven drying, mold drying, core drying, air handling, etc.)
- [ ] Engine loading or preparation
- [ ] Fabrication
- [ ] Forklift operation
- [ ] Heat treatment
- [ ] Ice making
- [ ] Impregnation
- [ ] Machine injection
- [ ] Melting or pre-melting
- [ ] Metal treatment or pre-treatment
- [ ] Painting
- [ ] Power generation

- [ ] Smelting
- [ ] Standby or back-up power generation
- [ ] Steel cutting
- [ ] Thermal oxidation
- [ ] Transportation and logistics (e.g. trucking, distribution, delivery, etc.)
- [ ] Other (please specify)

III. Utilities

For the next set of questions, kindly refer to your water and electricity bills.

* 24. For the past 3 months, what was your average **MONTHLY ELECTRICITY** consumption in **KILOWATT-HOURS?**

Conversion:
1 megawatt-hour = 1 000 kilowatt-hours

- ◯ 1 to 10,000
- ◯ 10,001 to 20,000
- ◯ 20,001 to 30,000
- ◯ 30,001 to 40,000
- ◯ 40,001 to 50,000
- ◯ 50,001 and above

* 25. For the past 3 months, what was your average **MONTHLY WATER** consumption in **CUBIC METERS?**

- ◯ 1 to 1,000
- ◯ 1,001 to 2,000
- ◯ 2,001 to 3,000
- ◯ 3,001 to 4,000
- ◯ 4,001 to 5,000
- ◯ 5,001 and above

* 26. For the past 3 months, how much did you spend for **MONTHLY ELECTRICITY in PESOS?**

- ◯ 1 to 100,000
- ◯ 100,001 to 200,000
- ◯ 200,001 to 300,000
- ◯ 300,001 to 400,000
- ◯ 400,001 to 500,000
- ◯ 500,001 and above

* 27. For the past 3 months, how much did you spend for **MONTHLY WATER in PESOS?**

- ◯ 1 to 75,000
- ◯ 75,001 to 150,000
- ◯ 150,001 to 225,000
- ◯ 225,001 to 300,000
- ◯ 300,001 to 375,000
- ◯ 375,001 and above

III. Utilities

* 28. If you can switch to another electricity provider, what are your considerations? Please check all that apply.

- [ ] Price
- [ ] Supply stability and reliability
- [ ] Safety and security
- [ ] Environmental concerns
- [ ] Other (please specify)

III. Utilities

* 29. Have you participated in any PEZA-organized energy efficiency initiatives designed to improve operational efficiency and increase competitiveness?

- ○ Yes
- ○ No

* 30. Are you aware of any of the following?

	Yes	No
Energy management system (EnMS) standards (compliant with ISO 50001)	○	○
System optimization (SO) for steam, compressed air, and pumps	○	○
Financial opportunities for energy efficiency investments	○	○

* 31. Has your company implemented any measures to reduce energy consumption and cost?

- ○ Yes
- ○ No

III. Utilities

* 32. Which of the following measures has your company implemented to reduce energy consumption and cost? Please check all that apply.

- [ ] Installing solar panels
- [ ] Using inverter-type airconditioners
- [ ] Installing LED lighting
- [ ] Other (please specify)

III. Utilities

* 33. With 1 being the most important, rank the following reasons for not implementing or adopting any energy efficiency and conservation measures for operational efficiency.

You may choose a number from the dropdown, or drag and drop to reorder.

- Lack of understanding of the management on the advantages of adopting energy efficiency measures
- Lack of technical knowledge on how to implement energy efficient measures
- Lack of resources to switch to energy efficient equipment
- Energy efficiency not a company priority
- No perceived substantial effect in adopting energy efficient measures

IV. Fuels Used in Production

In this section, you will be asked about the importance, uses, consumption, and expenditure on different types of fuel (excluding electricity) in your main production processes. The different fuels are the following:

1. Biodiesel
2. Bunker
3. Coal
4. Diesel
5. Gasoline
6. Kerosene
7. LPG
8. Natural gas
9. Propane
10. Other fuels

IV. Fuels Used in Production - Biodiesel

* 34. Do you use **BIODIESEL** in your main production processes?

- ◯ Yes
- ◯ No

IV. Fuels Used in Production - Biodiesel

* 35. How important is **BIODIESEL** in your main production processes?

◯ Not important

◯ Slightly important

◯ Important

◯ Fairly important

◯ Very important

IV. Fuels Used in Production - Biodiesel

* 36. Which processes involved in your main production use **BIODIESEL**? Please check all that apply.

- [ ] Air conditioning
- [ ] Air/gas mixing (e.g. Selas mixing, etc.)
- [ ] Baking
- [ ] Boiler operation (e.g. for steam generation, etc.)
- [ ] Burning
- [ ] Curing (e.g. oven curing, powder paint curing, etc.)
- [ ] Die casting
- [ ] Drying or annealing (e.g. oven drying, mold drying, core drying, air handling, etc.)
- [ ] Engine loading or preparation
- [ ] Fabrication
- [ ] Forklift operation
- [ ] Heat treatment
- [ ] Ice making
- [ ] Impregnation
- [ ] Machine injection
- [ ] Melting or pre-melting
- [ ] Metal treatment or pre-treatment
- [ ] Painting
- [ ] Power generation

- [ ] Smelting
- [ ] Standby or back-up power generation
- [ ] Steel cutting
- [ ] Thermal oxidation
- [ ] Transportation and logistics (e.g. trucking, distribution, delivery, etc.)
- [ ] Other (please specify)

IV. Fuels Used in Production - Biodiesel

* 37. How much **BIODIESEL** do you consume (**IN LITERS**) for each process **PER DAY?**

Conversion:
1 cubic meter = 1,000 liters
1 US liquid gallon = 3.785 liters

	1 to 200	201 to 400	401 to 600	601 to 800	801 to 1,000	1,001 and above
Air conditioning						
Air/gas mixing (e.g. Selas mixing, etc.)						
Baking						
Boiler operation (e.g. for steam generation, etc.)						
Burning						
Curing (e.g. oven curing, powder paint curing, etc.)						
Die casting						
Drying or annealing (e.g. oven drying, mold drying, core drying, air handling, etc.)						
Engine loading or preparation						
Fabrication						
Forklift operation						
Heat treatment						

	1 to 200	201 to 400	401 to 600	601 to 800	801 to 1,000	1,001 and above
Ice making	◯	◯	◯	◯	◯	◯
Impregnation	◯	◯	◯	◯	◯	◯
Machine injection	◯	◯	◯	◯	◯	◯
Melting or pre-melting	◯	◯	◯	◯	◯	◯
Metal treatment or pre-treatment	◯	◯	◯	◯	◯	◯
Painting	◯	◯	◯	◯	◯	◯
Power generation	◯	◯	◯	◯	◯	◯
Smelting	◯	◯	◯	◯	◯	◯
Standby or back-up power generation	◯	◯	◯	◯	◯	◯
Steel cutting	◯	◯	◯	◯	◯	◯
Thermal oxidation	◯	◯	◯	◯	◯	◯
Transportation and logistics (e.g. trucking, distribution, delivery, etc.)	◯	◯	◯	◯	◯	◯
[Insert text from Other]	◯	◯	◯	◯	◯	◯

* 38. How much do you spend for your **BIODIESEL** consumption (**IN PESOS**) for each process **PER MONTH**?

	1 to 270,000	270,001 to 540,000	540,001 to 810,000	810,001 to 1,080,000	1,080,001 to 1,350,000	1,350,001 and above
Air conditioning	◯	◯	◯	◯	◯	◯
Air/gas mixing (e.g. Selas mixing, etc.)	◯	◯	◯	◯	◯	◯
Baking	◯	◯	◯	◯	◯	◯
Boiler operation (e.g. for steam generation, etc.)	◯	◯	◯	◯	◯	◯

	1 to 270,000	270,001 to 540,000	540,001 to 810,000	810,001 to 1,080,000	1,080,001 to 1,350,000	1,350,001 and above
Burning	◯	◯	◯	◯	◯	◯
Curing (e.g. oven curing, powder paint curing, etc.)	◯	◯	◯	◯	◯	◯
Die casting	◯	◯	◯	◯	◯	◯
Drying or annealing (e.g. oven drying, mold drying, core drying, air handling, etc.)	◯	◯	◯	◯	◯	◯
Engine loading or preparation	◯	◯	◯	◯	◯	◯
Fabrication	◯	◯	◯	◯	◯	◯
Forklift operation	◯	◯	◯	◯	◯	◯
Heat treatment	◯	◯	◯	◯	◯	◯
Ice making	◯	◯	◯	◯	◯	◯
Impregnation	◯	◯	◯	◯	◯	◯
Machine injection	◯	◯	◯	◯	◯	◯
Melting or pre-melting	◯	◯	◯	◯	◯	◯
Metal treatment or pre-treatment	◯	◯	◯	◯	◯	◯
Painting	◯	◯	◯	◯	◯	◯
Power generation	◯	◯	◯	◯	◯	◯
Smelting	◯	◯	◯	◯	◯	◯
Standby or back-up power generation	◯	◯	◯	◯	◯	◯
Steel cutting	◯	◯	◯	◯	◯	◯
Thermal oxidation	◯	◯	◯	◯	◯	◯

	1 to 270,000	270,001 to 540,000	540,001 to 810,000	810,001 to 1,080,000	1,080,001 to 1,350,000	1,350,001 and above
Transportation and logistics (e.g. trucking, distribution, delivery, etc.)	◯	◯	◯	◯	◯	◯
[Insert text from Other]	◯	◯	◯	◯	◯	◯

IV. Fuels Used in Production - Bunker

* 39. Do you use **BUNKER** in your main production processes?

◯ Yes

◯ No

IV. Fuels Used in Production - Bunker

* 40. How important is **BUNKER** in your main production processes?

- Not important
- Slightly important
- Important
- Fairly important
- Very important

IV. Fuels Used in Production - Bunker

* 41. Which processes involved in your main production use **BUNKER**? Please check all that apply.

- [ ] Air conditioning
- [ ] Air/gas mixing (e.g. Selas mixing, etc.)
- [ ] Baking
- [ ] Boiler operation (e.g. for steam generation, etc.)
- [ ] Burning
- [ ] Curing (e.g. oven curing, powder paint curing, etc.)
- [ ] Die casting
- [ ] Drying or annealing (e.g. oven drying, mold drying, core drying, air handling, etc.)
- [ ] Engine loading or preparation
- [ ] Fabrication
- [ ] Forklift operation
- [ ] Heat treatment
- [ ] Ice making
- [ ] Impregnation
- [ ] Machine injection
- [ ] Melting or pre-melting
- [ ] Metal treatment or pre-treatment
- [ ] Painting
- [ ] Power generation

- [ ] Smelting
- [ ] Standby or back-up power generation
- [ ] Steel cutting
- [ ] Thermal oxidation
- [ ] Transportation and logistics (e.g. trucking, distribution, delivery, etc.)
- [ ] Other (please specify)

IV. Fuels Used in Production - Bunker

* 42. How much **BUNKER** do you consume (**IN LITERS**) for each process **PER DAY?**

Conversion:
1 cubic meter = 1,000 liters
1 US liquid gallon = 3.785 liters

	1 to 200	201 to 400	401 to 600	601 to 800	801 to 1,000	1,001 and above
Air conditioning	◯	◯	◯	◯	◯	◯
Air/gas mixing (e.g. Selas mixing, etc.)	◯	◯	◯	◯	◯	◯
Baking	◯	◯	◯	◯	◯	◯
Boiler operation (e.g. for steam generation, etc.)	◯	◯	◯	◯	◯	◯
Burning	◯	◯	◯	◯	◯	◯
Curing (e.g. oven curing, powder paint curing, etc.)	◯	◯	◯	◯	◯	◯
Die casting	◯	◯	◯	◯	◯	◯
Drying or annealing (e.g. oven drying, mold drying, core drying, air handling, etc.)	◯	◯	◯	◯	◯	◯
Engine loading or preparation	◯	◯	◯	◯	◯	◯
Fabrication	◯	◯	◯	◯	◯	◯
Forklift operation	◯	◯	◯	◯	◯	◯
Heat treatment	◯	◯	◯	◯	◯	◯

	1 to 200	201 to 400	401 to 600	601 to 800	801 to 1,000	1,001 and above
Ice making	◯	◯	◯	◯	◯	◯
Impregnation	◯	◯	◯	◯	◯	◯
Machine injection	◯	◯	◯	◯	◯	◯
Melting or pre-melting	◯	◯	◯	◯	◯	◯
Metal treatment or pre-treatment	◯	◯	◯	◯	◯	◯
Painting	◯	◯	◯	◯	◯	◯
Power generation	◯	◯	◯	◯	◯	◯
Smelting	◯	◯	◯	◯	◯	◯
Standby or back-up power generation	◯	◯	◯	◯	◯	◯
Steel cutting	◯	◯	◯	◯	◯	◯
Thermal oxidation	◯	◯	◯	◯	◯	◯
Transportation and logistics (e.g. trucking, distribution, delivery, etc.)	◯	◯	◯	◯	◯	◯
[Insert text from Other]	◯	◯	◯	◯	◯	◯

* 43. How much do you spend for your **BUNKER** consumption (**IN PESOS**) for each process **PER MONTH**?

	1 to 270,000	270,001 to 540,000	540,001 to 810,000	810,001 to 1,080,000	1,080,001 to 1,350,000	1,350,001 and above
Air conditioning	◯	◯	◯	◯	◯	◯
Air/gas mixing (e.g. Selas mixing, etc.)	◯	◯	◯	◯	◯	◯
Baking	◯	◯	◯	◯	◯	◯
Boiler operation (e.g. for steam generation, etc.)	◯	◯	◯	◯	◯	◯
Burning	◯	◯	◯	◯	◯	◯

	1 to 270,000	270,001 to 540,000	540,001 to 810,000	810,001 to 1,080,000	1,080,001 to 1,350,000	1,350,001 and above
Curing (e.g. oven curing, powder paint curing, etc.)	◯	◯	◯	◯	◯	◯
Die casting	◯	◯	◯	◯	◯	◯
Drying or annealing (e.g. oven drying, mold drying, core drying, air handling, etc.)	◯	◯	◯	◯	◯	◯
Engine loading or preparation	◯	◯	◯	◯	◯	◯
Fabrication	◯	◯	◯	◯	◯	◯
Forklift operation	◯	◯	◯	◯	◯	◯
Heat treatment	◯	◯	◯	◯	◯	◯
Ice making	◯	◯	◯	◯	◯	◯
Impregnation	◯	◯	◯	◯	◯	◯
Machine injection	◯	◯	◯	◯	◯	◯
Melting or pre-melting	◯	◯	◯	◯	◯	◯
Metal treatment or pre-treatment	◯	◯	◯	◯	◯	◯
Painting	◯	◯	◯	◯	◯	◯
Power generation	◯	◯	◯	◯	◯	◯
Smelting	◯	◯	◯	◯	◯	◯
Standby or back-up power generation	◯	◯	◯	◯	◯	◯
Steel cutting	◯	◯	◯	◯	◯	◯
Thermal oxidation	◯	◯	◯	◯	◯	◯
Transportation and logistics (e.g. trucking, distribution, delivery, etc.)	◯	◯	◯	◯	◯	◯

	1 to 270,000	270,001 to 540,000	540,001 to 810,000	810,001 to 1,080,000	1,080,001 to 1,350,000	1,350,001 and above
[Insert text from Other]	○	○	○	○	○	○

IV. Fuels Used in Production - Coal

* 44. Do you use **COAL** in your main production processes?

- ◯ Yes
- ◯ No

IV. Fuels Used in Production - Coal

* 45. How important is **COAL** in your main production processes?

- ◯ Not important
- ◯ Slightly important
- ◯ Important
- ◯ Fairly important
- ◯ Very important

IV. Fuels Used in Production - Coal

* 46. Which processes involved in your main production use **COAL**? Please check all that apply.

- ☐ Air conditioning
- ☐ Air/gas mixing (e.g. Selas mixing, etc.)
- ☐ Baking
- ☐ Boiler operation (e.g. for steam generation, etc.)
- ☐ Burning
- ☐ Curing (e.g. oven curing, powder paint curing, etc.)
- ☐ Die casting
- ☐ Drying or annealing (e.g. oven drying, mold drying, core drying, air handling, etc.)
- ☐ Engine loading or preparation
- ☐ Fabrication
- ☐ Forklift operation
- ☐ Heat treatment
- ☐ Ice making
- ☐ Impregnation
- ☐ Machine injection
- ☐ Melting or pre-melting
- ☐ Metal treatment or pre-treatment
- ☐ Painting
- ☐ Power generation

- [ ] Smelting
- [ ] Standby or back-up power generation
- [ ] Steel cutting
- [ ] Thermal oxidation
- [ ] Transportation and logistics (e.g. trucking, distribution, delivery, etc.)
- [ ] Other (please specify)

IV. Fuels Used in Production - Coal

* 47. How much **COAL** do you consume (**IN METRIC TONS**) for each process **PER DAY?**

Conversion:
1 kg = 0.001 metric ton (tonne)
1 US ton = 0.907 metric ton (tonne)

	1 to 20	21 to 40	41 to 60	61 to 80	81 to 100	101 and above
Air conditioning	○	○	○	○	○	○
Air/gas mixing (e.g. Selas mixing, etc.)	○	○	○	○	○	○
Baking	○	○	○	○	○	○
Boiler operation (e.g. for steam generation, etc.)	○	○	○	○	○	○
Burning	○	○	○	○	○	○
Curing (e.g. oven curing, powder paint curing, etc.)	○	○	○	○	○	○
Die casting	○	○	○	○	○	○
Drying or annealing (e.g. oven drying, mold drying, core drying, air handling, etc.)	○	○	○	○	○	○
Engine loading or preparation	○	○	○	○	○	○
Fabrication	○	○	○	○	○	○
Forklift operation	○	○	○	○	○	○
Heat treatment	○	○	○	○	○	○

	1 to 20	21 to 40	41 to 60	61 to 80	81 to 100	101 and above
Ice making	◯	◯	◯	◯	◯	◯
Impregnation	◯	◯	◯	◯	◯	◯
Machine injection	◯	◯	◯	◯	◯	◯
Melting or pre-melting	◯	◯	◯	◯	◯	◯
Metal treatment or pre-treatment	◯	◯	◯	◯	◯	◯
Painting	◯	◯	◯	◯	◯	◯
Power generation	◯	◯	◯	◯	◯	◯
Smelting	◯	◯	◯	◯	◯	◯
Standby or back-up power generation	◯	◯	◯	◯	◯	◯
Steel cutting	◯	◯	◯	◯	◯	◯
Thermal oxidation	◯	◯	◯	◯	◯	◯
Transportation and logistics (e.g. trucking, distribution, delivery, etc.)	◯	◯	◯	◯	◯	◯
[Insert text from Other]	◯	◯	◯	◯	◯	◯

* 48. How much do you spend for your **COAL** consumption (**IN PESOS**) for each process **PER MONTH**?

	1 to 3,000,000	3,000,001 to 6,000,000	6,000,001 to 9,000,000	9,000,001 to 12,000,000	12,000,001 to 15,000,000	15,000,001 and above
Air conditioning	◯	◯	◯	◯	◯	◯
Air/gas mixing (e.g. Selas mixing, etc.)	◯	◯	◯	◯	◯	◯
Baking	◯	◯	◯	◯	◯	◯
Boiler operation (e.g. for steam generation, etc.)	◯	◯	◯	◯	◯	◯
Burning	◯	◯	◯	◯	◯	◯

	1 to 3,000,000	3,000,001 to 6,000,000	6,000,001 to 9,000,000	9,000,001 to 12,000,000	12,000,001 to 15,000,000	15,000,001 and above
Curing (e.g. oven curing, powder paint curing, etc.)	◯	◯	◯	◯	◯	◯
Die casting	◯	◯	◯	◯	◯	◯
Drying or annealing (e.g. oven drying, mold drying, core drying, air handling, etc.)	◯	◯	◯	◯	◯	◯
Engine loading or preparation	◯	◯	◯	◯	◯	◯
Fabrication	◯	◯	◯	◯	◯	◯
Forklift operation	◯	◯	◯	◯	◯	◯
Heat treatment	◯	◯	◯	◯	◯	◯
Ice making	◯	◯	◯	◯	◯	◯
Impregnation	◯	◯	◯	◯	◯	◯
Machine injection	◯	◯	◯	◯	◯	◯
Melting or pre-melting	◯	◯	◯	◯	◯	◯
Metal treatment or pre-treatment	◯	◯	◯	◯	◯	◯
Painting	◯	◯	◯	◯	◯	◯
Power generation	◯	◯	◯	◯	◯	◯
Smelting	◯	◯	◯	◯	◯	◯
Standby or back-up power generation	◯	◯	◯	◯	◯	◯
Steel cutting	◯	◯	◯	◯	◯	◯
Thermal oxidation	◯	◯	◯	◯	◯	◯

	1 to 3,000,000	3,000,001 to 6,000,000	6,000,001 to 9,000,000	9,000,001 to 12,000,000	12,000,001 to 15,000,000	15,000,001 and above
Transportation and logistics (e.g. trucking, distribution, delivery, etc.)	○	○	○	○	○	○
[Insert text from Other]	○	○	○	○	○	○

IV. Fuels Used in Production - Diesel

* 49. Do you use **DIESEL** in your main production processes?

- ◯ Yes
- ◯ No

IV. Fuels Used in Production - Diesel

* 50. How important is **DIESEL** in your main production processes?

- ◯ Not important
- ◯ Slightly important
- ◯ Important
- ◯ Fairly important
- ◯ Very important

IV. Fuels Used in Production - Diesel

* 51. Which processes involved in your main production use **DIESEL**? Please check all that apply.

- ☐ Air conditioning
- ☐ Air/gas mixing (e.g. Selas mixing, etc.)
- ☐ Baking
- ☐ Boiler operation (e.g. for steam generation, etc.)
- ☐ Burning
- ☐ Curing (e.g. oven curing, powder paint curing, etc.)
- ☐ Die casting
- ☐ Drying or annealing (e.g. oven drying, mold drying, core drying, air handling, etc.)
- ☐ Engine loading or preparation
- ☐ Fabrication
- ☐ Forklift operation
- ☐ Heat treatment
- ☐ Ice making
- ☐ Impregnation
- ☐ Machine injection
- ☐ Melting or pre-melting
- ☐ Metal treatment or pre-treatment
- ☐ Painting
- ☐ Power generation

- [ ] Smelting
- [ ] Standby or back-up power generation
- [ ] Steel cutting
- [ ] Thermal oxidation
- [ ] Transportation and logistics (e.g. trucking, distribution, delivery, etc.)
- [ ] Other (please specify)

IV. Fuels Used in Production - Diesel

* 52. How much **DIESEL** do you consume (**IN LITERS**) for each process **PER DAY?**

Conversion:
1 cubic meter = 1,000 liters
1 US liquid gallon = 3.785 liters

	1 to 200	201 to 400	401 to 600	601 to 800	801 to 1,000	1,001 and above
Air conditioning	○	○	○	○	○	○
Air/gas mixing (e.g. Selas mixing, etc.)	○	○	○	○	○	○
Baking	○	○	○	○	○	○
Boiler operation (e.g. for steam generation, etc.)	○	○	○	○	○	○
Burning	○	○	○	○	○	○
Curing (e.g. oven curing, powder paint curing, etc.)	○	○	○	○	○	○
Die casting	○	○	○	○	○	○
Drying or annealing (e.g. oven drying, mold drying, core drying, air handling, etc.)	○	○	○	○	○	○
Engine loading or preparation	○	○	○	○	○	○
Fabrication	○	○	○	○	○	○
Forklift operation	○	○	○	○	○	○
Heat treatment	○	○	○	○	○	○

	1 to 200	201 to 400	401 to 600	601 to 800	801 to 1,000	1,001 and above
Ice making	◯	◯	◯	◯	◯	◯
Impregnation	◯	◯	◯	◯	◯	◯
Machine injection	◯	◯	◯	◯	◯	◯
Melting or pre-melting	◯	◯	◯	◯	◯	◯
Metal treatment or pre-treatment	◯	◯	◯	◯	◯	◯
Painting	◯	◯	◯	◯	◯	◯
Power generation	◯	◯	◯	◯	◯	◯
Smelting	◯	◯	◯	◯	◯	◯
Standby or back-up power generation	◯	◯	◯	◯	◯	◯
Steel cutting	◯	◯	◯	◯	◯	◯
Thermal oxidation	◯	◯	◯	◯	◯	◯
Transportation and logistics (e.g. trucking, distribution, delivery, etc.)	◯	◯	◯	◯	◯	◯
[Insert text from Other]	◯	◯	◯	◯	◯	◯

* 53. How much do you spend for your **DIESEL** consumption (**IN PESOS**) for each process **PER MONTH**?

	1 to 270,000	270,001 to 540,000	540,001 to 810,000	810,001 to 1,080,000	1,080,001 to 1,350,000	1,350,001 and above
Air conditioning	◯	◯	◯	◯	◯	◯
Air/gas mixing (e.g. Selas mixing, etc.)	◯	◯	◯	◯	◯	◯
Baking	◯	◯	◯	◯	◯	◯
Boiler operation (e.g. for steam generation, etc.)	◯	◯	◯	◯	◯	◯
Burning	◯	◯	◯	◯	◯	◯

	1 to 270,000	270,001 to 540,000	540,001 to 810,000	810,001 to 1,080,000	1,080,001 to 1,350,000	1,350,001 and above
Curing (e.g. oven curing, powder paint curing, etc.)	○	○	○	○	○	○
Die casting	○	○	○	○	○	○
Drying or annealing (e.g. oven drying, mold drying, core drying, air handling, etc.)	○	○	○	○	○	○
Engine loading or preparation	○	○	○	○	○	○
Fabrication	○	○	○	○	○	○
Forklift operation	○	○	○	○	○	○
Heat treatment	○	○	○	○	○	○
Ice making	○	○	○	○	○	○
Impregnation	○	○	○	○	○	○
Machine injection	○	○	○	○	○	○
Melting or pre-melting	○	○	○	○	○	○
Metal treatment or pre-treatment	○	○	○	○	○	○
Painting	○	○	○	○	○	○
Power generation	○	○	○	○	○	○
Smelting	○	○	○	○	○	○
Standby or back-up power generation	○	○	○	○	○	○
Steel cutting	○	○	○	○	○	○
Thermal oxidation	○	○	○	○	○	○
Transportation and logistics (e.g. trucking, distribution, delivery, etc.)	○	○	○	○	○	○

	1 to 270,000	270,001 to 540,000	540,001 to 810,000	810,001 to 1,080,000	1,080,001 to 1,350,000	1,350,001 and above
[Insert text from Other]	○	○	○	○	○	○

IV. Fuels Used in Production - Gasoline

* 54. Do you use **GASOLINE** in your main production processes?

◯ Yes

◯ No

IV. Fuels Used in Production - Gasoline

* 55. How important is **GASOLINE** in your main production processes?

- ◯ Not important
- ◯ Slightly important
- ◯ Important
- ◯ Fairly important
- ◯ Very important

IV. Fuels Used in Production - Gasoline

* 56. Which processes involved in your main production use **GASOLINE?** Please check all that apply.

- [ ] Air conditioning
- [ ] Air/gas mixing (e.g. Selas mixing, etc.)
- [ ] Baking
- [ ] Boiler operation (e.g. for steam generation, etc.)
- [ ] Burning
- [ ] Curing (e.g. oven curing, powder paint curing, etc.)
- [ ] Die casting
- [ ] Drying or annealing (e.g. oven drying, mold drying, core drying, air handling, etc.)
- [ ] Engine loading or preparation
- [ ] Fabrication
- [ ] Forklift operation
- [ ] Heat treatment
- [ ] Ice making
- [ ] Impregnation
- [ ] Machine injection
- [ ] Melting or pre-melting
- [ ] Metal treatment or pre-treatment
- [ ] Painting
- [ ] Power generation

- [ ] Smelting
- [ ] Standby or back-up power generation
- [ ] Steel cutting
- [ ] Thermal oxidation
- [ ] Transportation and logistics (e.g. trucking, distribution, delivery, etc.)
- [ ] Other (please specify)

IV. Fuels Used in Production - Gasoline

* 57. How much **GASOLINE** do you consume (**IN LITERS**) for each process **PER DAY?**

Conversion:
1 cubic meter = 1,000 liters
1 US liquid gallon = 3.785 liters

	1 to 200	201 to 400	401 to 600	601 to 800	801 to 1,000	1,001 and above
Air conditioning	○	○	○	○	○	○
Air/gas mixing (e.g. Selas mixing, etc.)	○	○	○	○	○	○
Baking	○	○	○	○	○	○
Boiler operation (e.g. for steam generation, etc.)	○	○	○	○	○	○
Burning	○	○	○	○	○	○
Curing (e.g. oven curing, powder paint curing, etc.)	○	○	○	○	○	○
Die casting	○	○	○	○	○	○
Drying or annealing (e.g. oven drying, mold drying, core drying, air handling, etc.)	○	○	○	○	○	○
Engine loading or preparation	○	○	○	○	○	○
Fabrication	○	○	○	○	○	○
Forklift operation	○	○	○	○	○	○
Heat treatment	○	○	○	○	○	○

	1 to 200	201 to 400	401 to 600	601 to 800	801 to 1,000	1,001 and above
Ice making						
Impregnation						
Machine injection						
Melting or pre-melting						
Metal treatment or pre-treatment						
Painting						
Power generation						
Smelting						
Standby or back-up power generation						
Steel cutting						
Thermal oxidation						
Transportation and logistics (e.g. trucking, distribution, delivery, etc.)						
[Insert text from Other]						

* 58. How much do you spend for your **GASOLINE** consumption (**IN PESOS**) for each process **PER MONTH?**

	1 to 360,000	360,001 to 720,000	720,001 to 1,080,000	1,080,001 to 1,440,000	1,440,001 to 1,800,000	1,800,001 and above
Air conditioning						
Air/gas mixing (e.g. Selas mixing, etc.)						
Baking						
Boiler operation (e.g. for steam generation, etc.)						
Burning						

	1 to 360,000	360,001 to 720,000	720,001 to 1,080,000	1,080,001 to 1,440,000	1,440,001 to 1,800,000	1,800,001 and above
Curing (e.g. oven curing, powder paint curing, etc.)	○	○	○	○	○	○
Die casting	○	○	○	○	○	○
Drying or annealing (e.g. oven drying, mold drying, core drying, air handling, etc.)	○	○	○	○	○	○
Engine loading or preparation	○	○	○	○	○	○
Fabrication	○	○	○	○	○	○
Forklift operation	○	○	○	○	○	○
Heat treatment	○	○	○	○	○	○
Ice making	○	○	○	○	○	○
Impregnation	○	○	○	○	○	○
Machine injection	○	○	○	○	○	○
Melting or pre-melting	○	○	○	○	○	○
Metal treatment or pre-treatment	○	○	○	○	○	○
Painting	○	○	○	○	○	○
Power generation	○	○	○	○	○	○
Smelting	○	○	○	○	○	○
Standby or back-up power generation	○	○	○	○	○	○
Steel cutting	○	○	○	○	○	○
Thermal oxidation	○	○	○	○	○	○
Transportation and logistics (e.g. trucking, distribution, delivery, etc.)	○	○	○	○	○	○

	1 to 360,000	360,001 to 720,000	720,001 to 1,080,000	1,080,001 to 1,440,000	1,440,001 to 1,800,000	1,800,001 and above
[Insert text from Other]	○	○	○	○	○	○

IV. Fuels Used in Production - Kerosene

* 59. Do you use **KEROSENE** in your main production processes?

◯ Yes

◯ No

IV. Fuels Used in Production - Kerosene

* 60. How important is **KEROSENE** in your main production processes?

- ◯ Not important
- ◯ Slightly important
- ◯ Important
- ◯ Fairly important
- ◯ Very important

IV. Fuels Used in Production - Kerosene

* 61. Which processes involved in your main production use **KEROSENE**? Please check all that apply.

- [ ] Air conditioning
- [ ] Air/gas mixing (e.g. Selas mixing, etc.)
- [ ] Baking
- [ ] Boiler operation (e.g. for steam generation, etc.)
- [ ] Burning
- [ ] Curing (e.g. oven curing, powder paint curing, etc.)
- [ ] Die casting
- [ ] Drying or annealing (e.g. oven drying, mold drying, core drying, air handling, etc.)
- [ ] Engine loading or preparation
- [ ] Fabrication
- [ ] Forklift operation
- [ ] Heat treatment
- [ ] Ice making
- [ ] Impregnation
- [ ] Machine injection
- [ ] Melting or pre-melting
- [ ] Metal treatment or pre-treatment
- [ ] Painting
- [ ] Power generation

- [ ] Smelting
- [ ] Standby or back-up power generation
- [ ] Steel cutting
- [ ] Thermal oxidation
- [ ] Transportation and logistics (e.g. trucking, distribution, delivery, etc.)
- [ ] Other (please specify)

IV. Fuels Used in Production - Kerosene

* 62. How much **KEROSENE** do you consume (**IN LITERS**) for each process **PER DAY**?

Conversion:
1 cubic meter = 1,000 liters
1 US liquid gallon = 3.785 liters

	1 to 200	201 to 400	401 to 600	601 to 800	801 to 1,000	1,001 and above
Air conditioning	◯	◯	◯	◯	◯	◯
Air/gas mixing (e.g. Selas mixing, etc.)	◯	◯	◯	◯	◯	◯
Baking	◯	◯	◯	◯	◯	◯
Boiler operation (e.g. for steam generation, etc.)	◯	◯	◯	◯	◯	◯
Burning	◯	◯	◯	◯	◯	◯
Curing (e.g. oven curing, powder paint curing, etc.)	◯	◯	◯	◯	◯	◯
Die casting	◯	◯	◯	◯	◯	◯
Drying or annealing (e.g. oven drying, mold drying, core drying, air handling, etc.)	◯	◯	◯	◯	◯	◯
Engine loading or preparation	◯	◯	◯	◯	◯	◯
Fabrication	◯	◯	◯	◯	◯	◯
Forklift operation	◯	◯	◯	◯	◯	◯
Heat treatment	◯	◯	◯	◯	◯	◯

	1 to 200	201 to 400	401 to 600	601 to 800	801 to 1,000	1,001 and above
Ice making	○	○	○	○	○	○
Impregnation	○	○	○	○	○	○
Machine injection	○	○	○	○	○	○
Melting or pre-melting	○	○	○	○	○	○
Metal treatment or pre-treatment	○	○	○	○	○	○
Painting	○	○	○	○	○	○
Power generation	○	○	○	○	○	○
Smelting	○	○	○	○	○	○
Standby or back-up power generation	○	○	○	○	○	○
Steel cutting	○	○	○	○	○	○
Thermal oxidation	○	○	○	○	○	○
Transportation and logistics (e.g. trucking, distribution, delivery, etc.)	○	○	○	○	○	○
[Insert text from Other]	○	○	○	○	○	○

* 63. How much do you spend for your **KEROSENE** consumption (**IN PESOS**) for each process **PER MONTH?**

	1 to 300,000	300,001 to 600,000	600,001 to 900,000	900,001 to 1,200,000	1,200,001 to 1,500,000	1,500,001 and above
Air conditioning	○	○	○	○	○	○
Air/gas mixing (e.g. Selas mixing, etc.)	○	○	○	○	○	○
Baking	○	○	○	○	○	○
Boiler operation (e.g. for steam generation, etc.)	○	○	○	○	○	○
Burning	○	○	○	○	○	○

	1 to 300,000	300,001 to 600,000	600,001 to 900,000	900,001 to 1,200,000	1,200,001 to 1,500,000	1,500,001 and above
Curing (e.g. oven curing, powder paint curing, etc.)	◯	◯	◯	◯	◯	◯
Die casting	◯	◯	◯	◯	◯	◯
Drying or annealing (e.g. oven drying, mold drying, core drying, air handling, etc.)	◯	◯	◯	◯	◯	◯
Engine loading or preparation	◯	◯	◯	◯	◯	◯
Fabrication	◯	◯	◯	◯	◯	◯
Forklift operation	◯	◯	◯	◯	◯	◯
Heat treatment	◯	◯	◯	◯	◯	◯
Ice making	◯	◯	◯	◯	◯	◯
Impregnation	◯	◯	◯	◯	◯	◯
Machine injection	◯	◯	◯	◯	◯	◯
Melting or pre-melting	◯	◯	◯	◯	◯	◯
Metal treatment or pre-treatment	◯	◯	◯	◯	◯	◯
Painting	◯	◯	◯	◯	◯	◯
Power generation	◯	◯	◯	◯	◯	◯
Smelting	◯	◯	◯	◯	◯	◯
Standby or back-up power generation	◯	◯	◯	◯	◯	◯
Steel cutting	◯	◯	◯	◯	◯	◯
Thermal oxidation	◯	◯	◯	◯	◯	◯
Transportation and logistics (e.g. trucking, distribution, delivery, etc.)	◯	◯	◯	◯	◯	◯

	1 to 300,000	300,001 to 600,000	600,001 to 900,000	900,001 to 1,200,000	1,200,001 to 1,500,000	1,500,001 and above
[Insert text from Other]	◯	◯	◯	◯	◯	◯

IV. Fuels Used in Production - LPG

* 64. Do you use **LPG** in your main production processes?

- ◯ Yes
- ◯ No

IV. Fuels Used in Production - LPG

* 65. How important is **LPG** in your main production processes?

- ◯ Not important
- ◯ Slightly important
- ◯ Important
- ◯ Fairly important
- ◯ Very important

IV. Fuels Used in Production - LPG

* 66. Which processes involved in your main production use **LPG**? Please check all that apply.

- ☐ Air conditioning
- ☐ Air/gas mixing (e.g. Selas mixing, etc.)
- ☐ Baking
- ☐ Boiler operation (e.g. for steam generation, etc.)
- ☐ Burning
- ☐ Curing (e.g. oven curing, powder paint curing, etc.)
- ☐ Die casting
- ☐ Drying or annealing (e.g. oven drying, mold drying, core drying, air handling, etc.)
- ☐ Engine loading or preparation
- ☐ Fabrication
- ☐ Forklift operation
- ☐ Heat treatment
- ☐ Ice making
- ☐ Impregnation
- ☐ Machine injection
- ☐ Melting or pre-melting
- ☐ Metal treatment or pre-treatment
- ☐ Painting
- ☐ Power generation

- [ ] Smelting
- [ ] Standby or back-up power generation
- [ ] Steel cutting
- [ ] Thermal oxidation
- [ ] Transportation and logistics (e.g. trucking, distribution, delivery, etc.)
- [ ] Other (please specify)

IV. Fuels Used in Production - LPG

* 67. How much **LPG** do you consume (**IN KILOGRAMS**) for each process **PER DAY?**

Conversion:
1 metric ton (tonne) = 1,000 kilograms
1 US ton = 907 kilograms

	1 to 200	201 to 400	401 to 600	601 to 800	801 to 1,000	1,001 and above
Air conditioning	○	○	○	○	○	○
Air/gas mixing (e.g. Selas mixing, etc.)	○	○	○	○	○	○
Baking	○	○	○	○	○	○
Boiler operation (e.g. for steam generation, etc.)	○	○	○	○	○	○
Burning	○	○	○	○	○	○
Curing (e.g. oven curing, powder paint curing, etc.)	○	○	○	○	○	○
Die casting	○	○	○	○	○	○
Drying or annealing (e.g. oven drying, mold drying, core drying, air handling, etc.)	○	○	○	○	○	○
Engine loading or preparation	○	○	○	○	○	○
Fabrication	○	○	○	○	○	○
Forklift operation	○	○	○	○	○	○
Heat treatment	○	○	○	○	○	○

	1 to 200	201 to 400	401 to 600	601 to 800	801 to 1,000	1,001 and above
Ice making	◯	◯	◯	◯	◯	◯
Impregnation	◯	◯	◯	◯	◯	◯
Machine injection	◯	◯	◯	◯	◯	◯
Melting or pre-melting	◯	◯	◯	◯	◯	◯
Metal treatment or pre-treatment	◯	◯	◯	◯	◯	◯
Painting	◯	◯	◯	◯	◯	◯
Power generation	◯	◯	◯	◯	◯	◯
Smelting	◯	◯	◯	◯	◯	◯
Standby or back-up power generation	◯	◯	◯	◯	◯	◯
Steel cutting	◯	◯	◯	◯	◯	◯
Thermal oxidation	◯	◯	◯	◯	◯	◯
Transportation and logistics (e.g. trucking, distribution, delivery, etc.)	◯	◯	◯	◯	◯	◯
[Insert text from Other]	◯	◯	◯	◯	◯	◯

* 68. How much do you spend for your **LPG** consumption (**IN PESOS**) for each process **PER MONTH?**

	1 to 360,000	360,001 to 720,000	720,001 to 1,080,000	1,080,001 to 1,440,000	1,440,001 to 1,800,000	1,800,001 and above
Air conditioning	◯	◯	◯	◯	◯	◯
Air/gas mixing (e.g. Selas mixing, etc.)	◯	◯	◯	◯	◯	◯
Baking	◯	◯	◯	◯	◯	◯

	1 to 360,000	360,001 to 720,000	720,001 to 1,080,000	1,080,001 to 1,440,000	1,440,001 to 1,800,000	1,800,001 and above
Boiler operation (e.g. for steam generation, etc.)	○	○	○	○	○	○
Burning	○	○	○	○	○	○
Curing (e.g. oven curing, powder paint curing, etc.)	○	○	○	○	○	○
Die casting	○	○	○	○	○	○
Drying or annealing (e.g. oven drying, mold drying, core drying, air handling, etc.)	○	○	○	○	○	○
Engine loading or preparation	○	○	○	○	○	○
Fabrication	○	○	○	○	○	○
Forklift operation	○	○	○	○	○	○
Heat treatment	○	○	○	○	○	○
Ice making	○	○	○	○	○	○
Impregnation	○	○	○	○	○	○
Machine injection	○	○	○	○	○	○
Melting or pre-melting	○	○	○	○	○	○
Metal treatment or pre-treatment	○	○	○	○	○	○
Painting	○	○	○	○	○	○
Power generation	○	○	○	○	○	○
Smelting	○	○	○	○	○	○
Standby or back-up power generation	○	○	○	○	○	○
Steel cutting	○	○	○	○	○	○

	1 to 360,000	360,001 to 720,000	720,001 to 1,080,000	1,080,001 to 1,440,000	1,440,001 to 1,800,000	1,800,001 and above
Thermal oxidation	○	○	○	○	○	○
Transportation and logistics (e.g. trucking, distribution, delivery, etc.)	○	○	○	○	○	○
[Insert text from Other]	○	○	○	○	○	○

IV. Fuels Used in Production - Natural Gas

* 69. Do you use **NATURAL GAS** in your main production processes?

◯ Yes

◯ No

IV. Fuels Used in Production - Natural Gas

* 70. How important is **NATURAL GAS** in your main production processes?

- ◯ Not important
- ◯ Slightly important
- ◯ Important
- ◯ Fairly important
- ◯ Very important

IV. Fuels Used in Production - Natural Gas

* 71. Which processes involved in your main production use **NATURAL GAS**? Please check all that apply.

- [ ] Air conditioning
- [ ] Air/gas mixing (e.g. Selas mixing, etc.)
- [ ] Baking
- [ ] Boiler operation (e.g. for steam generation, etc.)
- [ ] Burning
- [ ] Curing (e.g. oven curing, powder paint curing, etc.)
- [ ] Die casting
- [ ] Drying or annealing (e.g. oven drying, mold drying, core drying, air handling, etc.)
- [ ] Engine loading or preparation
- [ ] Fabrication
- [ ] Forklift operation
- [ ] Heat treatment
- [ ] Ice making
- [ ] Impregnation
- [ ] Machine injection
- [ ] Melting or pre-melting
- [ ] Metal treatment or pre-treatment
- [ ] Painting
- [ ] Power generation

- [ ] Smelting
- [ ] Standby or back-up power generation
- [ ] Steel cutting
- [ ] Thermal oxidation
- [ ] Transportation and logistics (e.g. trucking, distribution, delivery, etc.)
- [ ] Other (please specify)

IV. Fuels Used in Production - Natural Gas

* 72. How much **NATURAL GAS** do you consume (**IN MILLION STANDARD CUBIC FEET PER DAY or MMSCFD**) for each process **PER DAY**?

	1 to 20	21 to 40	41 to 60	61 to 80	81 to 100	101 and above
Air conditioning	○	○	○	○	○	○
Air/gas mixing (e.g. Selas mixing, etc.)	○	○	○	○	○	○
Baking	○	○	○	○	○	○
Boiler operation (e.g. for steam generation, etc.)	○	○	○	○	○	○
Burning	○	○	○	○	○	○
Curing (e.g. oven curing, powder paint curing, etc.)	○	○	○	○	○	○
Die casting	○	○	○	○	○	○
Drying or annealing (e.g. oven drying, mold drying, core drying, air handling, etc.)	○	○	○	○	○	○
Engine loading or preparation	○	○	○	○	○	○
Fabrication	○	○	○	○	○	○
Forklift operation	○	○	○	○	○	○
Heat treatment	○	○	○	○	○	○
Ice making	○	○	○	○	○	○
Impregnation	○	○	○	○	○	○

	1 to 20	21 to 40	41 to 60	61 to 80	81 to 100	101 and above
Machine injection	◯	◯	◯	◯	◯	◯
Melting or pre-melting	◯	◯	◯	◯	◯	◯
Metal treatment or pre-treatment	◯	◯	◯	◯	◯	◯
Painting	◯	◯	◯	◯	◯	◯
Power generation	◯	◯	◯	◯	◯	◯
Smelting	◯	◯	◯	◯	◯	◯
Standby or back-up power generation	◯	◯	◯	◯	◯	◯
Steel cutting	◯	◯	◯	◯	◯	◯
Thermal oxidation	◯	◯	◯	◯	◯	◯
Transportation and logistics (e.g. trucking, distribution, delivery, etc.)	◯	◯	◯	◯	◯	◯
[Insert text from Other]	◯	◯	◯	◯	◯	◯

* 73. How much do you spend for your **NATURAL GAS** consumption (**IN PESOS**) for each process **PER MONTH?**

	1 to 360,000	360,001 to 720,000	720,001 to 1,080,000	1,080,001 to 1,440,000	1,440,001 to 1,800,000	1,800,001 and above
Air conditioning	◯	◯	◯	◯	◯	◯
Air/gas mixing (e.g. Selas mixing, etc.)	◯	◯	◯	◯	◯	◯
Baking	◯	◯	◯	◯	◯	◯
Boiler operation (e.g. for steam generation, etc.)	◯	◯	◯	◯	◯	◯
Burning	◯	◯	◯	◯	◯	◯

	1 to 360,000	360,001 to 720,000	720,001 to 1,080,000	1,080,001 to 1,440,000	1,440,001 to 1,800,000	1,800,001 and above
Curing (e.g. oven curing, powder paint curing, etc.)	◯	◯	◯	◯	◯	◯
Die casting	◯	◯	◯	◯	◯	◯
Drying or annealing (e.g. oven drying, mold drying, core drying, air handling, etc.)	◯	◯	◯	◯	◯	◯
Engine loading or preparation	◯	◯	◯	◯	◯	◯
Fabrication	◯	◯	◯	◯	◯	◯
Forklift operation	◯	◯	◯	◯	◯	◯
Heat treatment	◯	◯	◯	◯	◯	◯
Ice making	◯	◯	◯	◯	◯	◯
Impregnation	◯	◯	◯	◯	◯	◯
Machine injection	◯	◯	◯	◯	◯	◯
Melting or pre-melting	◯	◯	◯	◯	◯	◯
Metal treatment or pre-treatment	◯	◯	◯	◯	◯	◯
Painting	◯	◯	◯	◯	◯	◯
Power generation	◯	◯	◯	◯	◯	◯
Smelting	◯	◯	◯	◯	◯	◯
Standby or back-up power generation	◯	◯	◯	◯	◯	◯
Steel cutting	◯	◯	◯	◯	◯	◯
Thermal oxidation	◯	◯	◯	◯	◯	◯

	1 to 360,000	360,001 to 720,000	720,001 to 1,080,000	1,080,001 to 1,440,000	1,440,001 to 1,800,000	1,800,001 and above
Transportation and logistics (e.g. trucking, distribution, delivery, etc.)	◯	◯	◯	◯	◯	◯
[Insert text from Other]	◯	◯	◯	◯	◯	◯

IV. Fuels Used in Production - Propane

* 74. Do you use **PROPANE** in your main production processes?

- Yes
- No

IV. Fuels Used in Production - Propane

* 75. How important is **PROPANE** in your main production processes?

- ◯ Not important
- ◯ Slightly important
- ◯ Important
- ◯ Fairly important
- ◯ Very important

IV. Fuels Used in Production - Propane

* 76. Which processes involved in your main production use **PROPANE**? Please check all that apply.

- [ ] Air conditioning
- [ ] Air/gas mixing (e.g. Selas mixing, etc.)
- [ ] Baking
- [ ] Boiler operation (e.g. for steam generation, etc.)
- [ ] Burning
- [ ] Curing (e.g. oven curing, powder paint curing, etc.)
- [ ] Die casting
- [ ] Drying or annealing (e.g. oven drying, mold drying, core drying, air handling, etc.)
- [ ] Engine loading or preparation
- [ ] Fabrication
- [ ] Forklift operation
- [ ] Heat treatment
- [ ] Ice making
- [ ] Impregnation
- [ ] Machine injection
- [ ] Melting or pre-melting
- [ ] Metal treatment or pre-treatment
- [ ] Painting
- [ ] Power generation

- [ ] Smelting
- [ ] Standby or back-up power generation
- [ ] Steel cutting
- [ ] Thermal oxidation
- [ ] Transportation and logistics (e.g. trucking, distribution, delivery, etc.)
- [ ] Other (please specify)

IV. Fuels Used in Production - Propane

* 77. How much **PROPANE** do you consume (**IN KILOGRAMS**) for each process **PER DAY?**

Conversion:
1 metric ton (tonne) = 1,000 kilograms
1 US ton = 907 kilograms

	1 to 200	201 to 400	401 to 600	601 to 800	801 to 1,000	1,001 and above
Air conditioning	○	○	○	○	○	○
Air/gas mixing (e.g. Selas mixing, etc.)	○	○	○	○	○	○
Baking	○	○	○	○	○	○
Boiler operation (e.g. for steam generation, etc.)	○	○	○	○	○	○
Burning	○	○	○	○	○	○
Curing (e.g. oven curing, powder paint curing, etc.)	○	○	○	○	○	○
Die casting	○	○	○	○	○	○
Drying or annealing (e.g. oven drying, mold drying, core drying, air handling, etc.)	○	○	○	○	○	○
Engine loading or preparation	○	○	○	○	○	○
Fabrication	○	○	○	○	○	○
Forklift operation	○	○	○	○	○	○
Heat treatment	○	○	○	○	○	○

	1 to 200	201 to 400	401 to 600	601 to 800	801 to 1,000	1,001 and above
Ice making						
Impregnation						
Machine injection						
Melting or pre-melting						
Metal treatment or pre-treatment						
Painting						
Power generation						
Smelting						
Standby or back-up power generation						
Steel cutting						
Thermal oxidation						
Transportation and logistics (e.g. trucking, distribution, delivery, etc.)						
[Insert text from Other]						

* 78. How much do you spend for your **PROPANE** consumption (**IN PESOS**) for each process **PER MONTH?**

	1 to 360,000	360,001 to 720,000	720,001 to 1,080,000	1,080,001 to 1,440,000	1,440,001 to 1,800,000	1,8[illegible]
Air conditioning						
Air/gas mixing (e.g. Selas mixing, etc.)						
Baking						

	1 to 360,000	360,001 to 720,000	720,001 to 1,080,000	1,080,001 to 1,440,000	1,440,001 to 1,800,000	1,8[illegible]
Boiler operation (e.g. for steam generation, etc.)	○	○	○	○	○	
Burning	○	○	○	○	○	
Curing (e.g. oven curing, powder paint curing, etc.)	○	○	○	○	○	
Die casting	○	○	○	○	○	
Drying or annealing (e.g. oven drying, mold drying, core drying, air handling, etc.)	○	○	○	○	○	
Engine loading or preparation	○	○	○	○	○	
Fabrication	○	○	○	○	○	
Forklift operation	○	○	○	○	○	
Heat treatment	○	○	○	○	○	
Ice making	○	○	○	○	○	
Impregnation	○	○	○	○	○	
Machine injection	○	○	○	○	○	
Melting or pre-melting	○	○	○	○	○	
Metal treatment or pre-treatment	○	○	○	○	○	
Painting	○	○	○	○	○	
Power generation	○	○	○	○	○	
Smelting	○	○	○	○	○	
Standby or back-up power generation	○	○	○	○	○	

	1 to 360,000	360,001 to 720,000	720,001 to 1,080,000	1,080,001 to 1,440,000	1,440,001 to 1,800,000	1,8[illegible]
Steel cutting	○	○	○	○	○	
Thermal oxidation	○	○	○	○	○	
Transportation and logistics (e.g. trucking, distribution, delivery, etc.)	○	○	○	○	○	
[Insert text from Other]	○	○	○	○	○	

IV. Fuels Used in Production - Other Fuel (1)

For the succeeding questions, you will be asked about other fuels that you use in your production processes, but were not mentioned previously.

* 79. Do you use **OTHER FUEL (1)** in your main production processes that was not mentioned in the previous questions?

◯ Yes

◯ No

If yes, which fuel?

IV. Fuels Used in Production - Other Fuel (1)

* 80. How important is **OTHER FUEL (1)** in your main production processes?

- ◯ Not important
- ◯ Slightly important
- ◯ Important
- ◯ Fairly important
- ◯ Very important

IV. Fuels Used in Production - Other Fuel (1)

* 81. Which processes involved in your main production use **OTHER FUEL (1)**? Please check all that apply.

- ☐ Air conditioning
- ☐ Air/gas mixing (e.g. Selas mixing, etc.)
- ☐ Baking
- ☐ Boiler operation (e.g. for steam generation, etc.)
- ☐ Burning
- ☐ Curing (e.g. oven curing, powder paint curing, etc.)
- ☐ Die casting
- ☐ Drying or annealing (e.g. oven drying, mold drying, core drying, air handling, etc.)
- ☐ Engine loading or preparation
- ☐ Fabrication
- ☐ Forklift operation
- ☐ Heat treatment
- ☐ Ice making
- ☐ Impregnation
- ☐ Machine injection
- ☐ Melting or pre-melting
- ☐ Metal treatment or pre-treatment
- ☐ Painting
- ☐ Power generation

- [ ] Smelting
- [ ] Standby or back-up power generation
- [ ] Steel cutting
- [ ] Thermal oxidation
- [ ] Transportation and logistics (e.g. trucking, distribution, delivery, etc.)
- [ ] Other (please specify)

IV. Fuels Used in Production - Other Fuel (1)

* 82. How much **OTHER FUEL (1)** do you consume for each process **PER DAY**? Please include quantity and unit.

Air conditioning	
Air/gas mixing (e.g. Selas mixing, etc.)	
Baking	
Boiler operation (e.g. for steam generation, etc.)	
Burning	
Curing (e.g. oven curing, powder paint curing, etc.)	
Die casting	
Drying or annealing (e.g. oven drying, mold drying, core drying, air handling, etc.)	
Engine loading or preparation	
Fabrication	
Forklift operation	
Heat treatment	
Ice making	
Impregnation	
Machine injection	

Melting or pre-melting

Metal treatment or pre-treatment

Painting

Power generation

Smelting

Standby or back-up power generation

Steel cutting

Thermal oxidation

Transportation and logistics (e.g. trucking, distribution, delivery, etc.)

[Insert text from Other]

* 83. How much do you spend for your **OTHER FUEL (1)** consumption (**IN PESOS**) for each process **PER MONTH?** Write only the amount.

Air conditioning

Air/gas mixing (e.g. Selas mixing, etc.)

Baking

Boiler operation (e.g. for steam generation, etc.)

Burning

Curing (e.g. oven curing, powder paint curing, etc.)

Die casting

Drying or annealing (e.g. oven drying, mold drying, core drying, air handling, etc.)	
Engine loading or preparation	
Fabrication	
Forklift operation	
Heat treatment	
Ice making	
Impregnation	
Machine injection	
Melting or pre-melting	
Metal treatment or pre-treatment	
Painting	
Power generation	
Smelting	
Standby or back-up power generation	
Steel cutting	
Thermal oxidation	
Transportation and logistics (e.g. trucking, distribution, delivery, etc.)	
[Insert text from Other]	

IV. Fuels Used in Production - Other Fuel (2)

* 84. Do you use **OTHER FUEL (2)** in your main production processes that was not mentioned in the previous questions?

- ◯ Yes
- ◯ No

If yes, which fuel?

IV. Fuels Used in Production - Other Fuel (2)

* 85. How important is **OTHER FUEL (2)** in your main production processes?

- ◯ Not important
- ◯ Slightly important
- ◯ Important
- ◯ Fairly important
- ◯ Very important

IV. Fuels Used in Production - Other Fuel (2)

* 86. Which processes involved in your main production use **OTHER FUEL (2)**? Please check all that apply.

- [ ] Air conditioning
- [ ] Air/gas mixing (e.g. Selas mixing, etc.)
- [ ] Baking
- [ ] Boiler operation (e.g. for steam generation, etc.)
- [ ] Burning
- [ ] Curing (e.g. oven curing, powder paint curing, etc.)
- [ ] Die casting
- [ ] Drying or annealing (e.g. oven drying, mold drying, core drying, air handling, etc.)
- [ ] Engine loading or preparation
- [ ] Fabrication
- [ ] Forklift operation
- [ ] Heat treatment
- [ ] Ice making
- [ ] Impregnation
- [ ] Machine injection
- [ ] Melting or pre-melting
- [ ] Metal treatment or pre-treatment
- [ ] Painting
- [ ] Power generation

- [ ] Smelting
- [ ] Standby or back-up power generation
- [ ] Steel cutting
- [ ] Thermal oxidation
- [ ] Transportation and logistics (e.g. trucking, distribution, delivery, etc.)
- [ ] Other (please specify)

IV. Fuels Used in Production - Other Fuel (2)

* 87. How much **OTHER FUEL (2)** do you consume for each process **PER DAY**? Please include quantity and unit.

Air conditioning	
Air/gas mixing (e.g. Selas mixing, etc.)	
Baking	
Boiler operation (e.g. for steam generation, etc.)	
Burning	
Curing (e.g. oven curing, powder paint curing, etc.)	
Die casting	
Drying or annealing (e.g. oven drying, mold drying, core drying, air handling, etc.)	
Engine loading or preparation	
Fabrication	
Forklift operation	
Heat treatment	
Ice making	
Impregnation	
Machine injection	

Melting or pre-melting

Metal treatment or pre-treatment

Painting

Power generation

Smelting

Standby or back-up power generation

Steel cutting

Thermal oxidation

Transportation and logistics (e.g. trucking, distribution, delivery, etc.)

[Insert text from Other]

* 88. How much do you spend for your **OTHER FUEL (2)** consumption (**IN PESOS**) for each process **PER MONTH?** Write only the amount.

Air conditioning

Air/gas mixing (e.g. Selas mixing, etc.)

Baking

Boiler operation (e.g. for steam generation, etc.)

Burning

Curing (e.g. oven curing, powder paint curing, etc.)

Die casting

Drying or annealing (e.g. oven drying, mold drying, core drying, air handling, etc.)	
Engine loading or preparation	
Fabrication	
Forklift operation	
Heat treatment	
Ice making	
Impregnation	
Machine injection	
Melting or pre-melting	
Metal treatment or pre-treatment	
Painting	
Power generation	
Smelting	
Standby or back-up power generation	
Steel cutting	
Thermal oxidation	
Transportation and logistics (e.g. trucking, distribution, delivery, etc.)	
[Insert text from Other]	

IV. Fuels Used in Production - Other Fuel (3)

* 89. Do you use **OTHER FUEL (3)** in your main production processes that was not mentioned in the previous questions?

- ◯ Yes
- ◯ No

If yes, which fuel?

____

IV. Fuels Used in Production - Other Fuel (3)

* 90. How important is **OTHER FUEL (3)** in your main production processes?

- ◯ Not important
- ◯ Slightly important
- ◯ Important
- ◯ Fairly important
- ◯ Very important

IV. Fuels Used in Production - Other Fuel (3)

* 91. Which processes involved in your main production use **OTHER FUEL (3)**? Please check all that apply.

- [ ] Air conditioning
- [ ] Air/gas mixing (e.g. Selas mixing, etc.)
- [ ] Baking
- [ ] Boiler operation (e.g. for steam generation, etc.)
- [ ] Burning
- [ ] Curing (e.g. oven curing, powder paint curing, etc.)
- [ ] Die casting
- [ ] Drying or annealing (e.g. oven drying, mold drying, core drying, air handling, etc.)
- [ ] Engine loading or preparation
- [ ] Fabrication
- [ ] Forklift operation
- [ ] Heat treatment
- [ ] Ice making
- [ ] Impregnation
- [ ] Machine injection
- [ ] Melting or pre-melting
- [ ] Metal treatment or pre-treatment
- [ ] Painting
- [ ] Power generation

- [ ] Smelting
- [ ] Standby or back-up power generation
- [ ] Steel cutting
- [ ] Thermal oxidation
- [ ] Transportation and logistics (e.g. trucking, distribution, delivery, etc.)
- [ ] Other (please specify)

IV. Fuels Used in Production - Other Fuel (3)

* 92. How much **OTHER FUEL (3)** do you consume for each process **PER DAY**? Please include quantity and unit.

Air conditioning	
Air/gas mixing (e.g. Selas mixing, etc.)	
Baking	
Boiler operation (e.g. for steam generation, etc.)	
Burning	
Curing (e.g. oven curing, powder paint curing, etc.)	
Die casting	
Drying or annealing (e.g. oven drying, mold drying, core drying, air handling, etc.)	
Engine loading or preparation	
Fabrication	
Forklift operation	
Heat treatment	
Ice making	
Impregnation	
Machine injection	

Melting or pre-melting

Metal treatment or pre-treatment

Painting

Power generation

Smelting

Standby or back-up power generation

Steel cutting

Thermal oxidation

Transportation and logistics (e.g. trucking, distribution, delivery, etc.)

[Insert text from Other]

* 93. How much do you spend for your **OTHER FUEL (3)** consumption (**IN PESOS**) for each process **PER MONTH?** Write only the amount.

Air conditioning

Air/gas mixing (e.g. Selas mixing, etc.)

Baking

Boiler operation (e.g. for steam generation, etc.)

Burning

Curing (e.g. oven curing, powder paint curing, etc.)

Die casting

Drying or annealing (e.g. oven drying, mold drying, core drying, air handling, etc.)	
Engine loading or preparation	
Fabrication	
Forklift operation	
Heat treatment	
Ice making	
Impregnation	
Machine injection	
Melting or pre-melting	
Metal treatment or pre-treatment	
Painting	
Power generation	
Smelting	
Standby or back-up power generation	
Steel cutting	
Thermal oxidation	
Transportation and logistics (e.g. trucking, distribution, delivery, etc.)	
[Insert text from Other]	

V. Aptitude on Alternative Fuels and Primary Energies

In this section, you will be asked about your knowledge, considerations, and opinions on alternative fuels and primary energies as well as your experiences in using them.

V. Aptitude on Alternative Fuels and Primary Energies - Natural Gas

Natural gas is different from LPG that is typically used for cooking.

* 94. With 1 being limited, and 5 being advanced, what is the extent of your knowledge on **NATURAL GAS** as fuel?

1	2	3	4	5
◯	◯	◯	◯	◯

* 95. Do you think the **NATURAL GAS** is safe to utilize as fuel in your production process?

- ◯ Yes
- ◯ No

* 96. Do you think **NATURAL GAS** is cost-competitive relative to the fuels and primary energies you are currently using?

- ◯ Yes
- ◯ No

* 97. How much (**IN PESOS**) do you think can you save if you use **NATURAL GAS?**

- ◯ 1 to 200,000
- ◯ 200,001 to 400,000
- ◯ 400,001 to 600,000
- ◯ 600,001 to 800,000
- ◯ 800,001 to 1,000,000
- ◯ 1,000,001 and above

* 98. In case **NATURAL GAS** would be made available to you, what would be your considerations in using it in your production processes? Please check all that apply.

- ☐ Price
- ☐ Supply stability and reliability
- ☐ Safety and security
- ☐ Environmental concerns
- ☐ Compatibility of machines and equipment
- ☐ Retrofitting costs of equipment
- ☐ Others (please specify)

99. If you chose "retrofitting costs of equipment" in the previous question, how much (**IN PESOS**) do you think will it cost?

- ◯ 1 to 200,000
- ◯ 200,001 to 400,000
- ◯ 400,001 to 600,000
- ◯ 600,001 to 800,000
- ◯ 800,001 to 1,000,000
- ◯ 1,000,001 and above

* 100. Are you open to switching to **NATURAL GAS** in you production processes, self-generation and back-up generation of power?

- ◯ Yes
- ◯ No

V. Aptitude on Alternative Fuels and Primary Energies - Natural Gas

* 101. In case you decide to switch to **NATURAL GAS** in your **PRODUCTION PROCESSES**, which among the following fuels would you most likely replace it with? Please check all that apply.

- ☐ Biodiesel
- ☐ Bunker
- ☐ Coal
- ☐ Diesel
- ☐ Gasoline
- ☐ Kerosene
- ☐ LPG
- ☐ Propane
- ☐ Other (please specify)

* 102. In case you decide to switch to **NATURAL GAS** in **SELF-GENERATION OF POWER**, which among the following fuels and primary energies would you most likely replace it with? Please check all that apply.

- [ ] Biodiesel
- [ ] Bunker
- [ ] Coal
- [ ] Diesel
- [ ] Gasoline
- [ ] Kerosene
- [ ] LPG
- [ ] Propane
- [ ] Solar
- [ ] Wind
- [ ] Other (please specify)

* 103. In case you decide to switch to **NATURAL GAS** in **BACK-UP POWER GENERATION**, which among the following fuels and primary energies would you most likely replace it with? Please check all that apply.

- [ ] Biodiesel
- [ ] Bunker
- [ ] Coal
- [ ] Diesel
- [ ] Gasoline
- [ ] Kerosene
- [ ] LPG
- [ ] Propane
- [ ] Solar
- [ ] Wind
- [ ] Other (please specify)

* 104. In case you decide to switch to **NATURAL GAS**, what would be your requirement **IN MILLION STANDARD CUBIC FEET PER DAY (MMSCFD)?**

- ◯ 1 to 20
- ◯ 21 to 40
- ◯ 41 to 60
- ◯ 61 to 80
- ◯ 81 to 100
- ◯ 101 and above

V. Aptitude on Alternative Fuels and Primary Energies - Natural Gas

* 105. Do you have a parent or partner company elsewhere that has any experiences using **NATURAL GAS** as fuel?

- ◯ Yes
- ◯ No

V. Aptitude on Alternative Fuels and Primary Energies - Solar

In the next questions, solar is defined as energy used to generate electricity through photovoltaic solar panels.

* 106. With 1 being limited, and 5 being advanced, what is the extent of your knowledge on **SOLAR** as primary energy?

1	2	3	4	5
○	○	○	○	○

* 107. Do you think the **SOLAR** is safe to utilize as primary energy in your production process?

- ○ Yes
- ○ No

* 108. Do you think **SOLAR** is cost-competitive relative to the fuels and primary energies you are currently using?

- ○ Yes
- ○ No

* 109. How much (**IN PESOS**) do you think can you save if you use **SOLAR**?

- ○ 1 to 200,000
- ○ 200,001 to 400,000
- ○ 400,001 to 600,000
- ○ 600,001 to 800,000
- ○ 800,001 to 1,000,000
- ○ 1,000,001

* 110. In case **SOLAR** would be made available to you, what would be your considerations in using it in your production processes? Please check all that apply.

- [ ] Price
- [ ] Supply stability and reliability
- [ ] Safety and security
- [ ] Environmental concerns
- [ ] Compatibility of machines and equipment
- [ ] Retrofitting costs of equipment
- [ ] Others (please specify)

111. If you chose "retrofitting costs of equipment" in the previous question, how much (**IN PESOS**) do you think will it cost?

- ◯ 1 to 200,000
- ◯ 200,001 to 400,000
- ◯ 400,001 to 600,000
- ◯ 600,001 to 800,000
- ◯ 800,001 to 1,000,000
- ◯ 1,000,001 and above

* 112. Are you open to switching to **SOLAR** in you production processes, self-generation and back-up generation of power?

- ◯ Yes
- ◯ No

V. Aptitude on Alternative Fuels and Primary Energies - Solar

* 113. In case you decide to switch to **SOLAR** in your **PRODUCTION PROCESSES**, which among the following fuels would you most likely replace it with? Please check all that apply.

- [ ] Biodiesel
- [ ] Bunker
- [ ] Coal
- [ ] Diesel
- [ ] Gasoline
- [ ] Kerosene
- [ ] LPG
- [ ] Propane
- [ ] Other (please specify)

* 114. In case you decide to switch to **SOLAR** in **SELF-GENERATION OF POWER**, which among the following fuels and primary energies would you most likely replace it with? Please check all that apply.

- [ ] Biodiesel
- [ ] Bunker
- [ ] Coal
- [ ] Diesel
- [ ] Gasoline
- [ ] Kerosene
- [ ] LPG
- [ ] Natural gas
- [ ] Propane
- [ ] Wind
- [ ] Other (please specify)

* 115. In case you decide to switch to **SOLAR** in **BACK-UP POWER GENERATION**, which among the following fuels and primary energies would you most likely replace it with? Please check all that apply.

- ☐ Biodiesel
- ☐ Bunker
- ☐ Coal
- ☐ Diesel
- ☐ Gasoline
- ☐ Kerosene
- ☐ LPG
- ☐ Natural gas
- ☐ Propane
- ☐ Wind
- ☐ Other (please specify)

* 116. In case you decide to switch to **SOLAR**, what would be your requirement **IN KILOWATT-HOURS?**

Conversion:
1 megawatt-hour = 1,000 kilowatt-hours

- ○ 1 to 10,000
- ○ 10,001 to 20,000
- ○ 20,001 to 30,000
- ○ 30,001 to 40,000
- ○ 40,001 to 50,000
- ○ 50,001 and above

V. Aptitude on Alternative Fuels and Primary Energies - Solar

* 117. Do you have a parent or partner company elsewhere that has any experiences using **SOLAR** as primary energy?

- ◯ Yes
- ◯ No

V. Aptitude on Alternative Fuels and Primary Energies - Wind

In the next questions, wind is defined as energy used to generate electricity through wind turbines or wind energy converters.

* 118. With 1 being limited, and 5 being advanced, what is the extent of your knowledge on **WIND** as primary energy?

1	2	3	4	5
◯	◯	◯	◯	◯

* 119. Do you think the **WIND** is safe to utilize as primary energy in your production process?

◯ Yes

◯ No

* 120. Do you think **WIND** is cost-competitive relative to the fuels and primary energies you are currently using?

◯ Yes

◯ No

* 121. How much (**IN PESOS**) do you think can you save if you use **WIND?**

◯ 1 to 200,000

◯ 200,001 to 400,000

◯ 400,001 to 600,000

◯ 600,001 to 800,000

◯ 800,001 to 1,000,000

◯ 1,000,001 and above

* 122. In case **WIND** would be made available to you, what would be your considerations in using it in your production processes? Please check all that apply.

- ☐ Price
- ☐ Supply stability and reliability
- ☐ Safety and security
- ☐ Environmental concerns
- ☐ Compatibility of machines and equipment
- ☐ Retrofitting costs of equipment
- ☐ Others (please specify)

123. If you chose "retrofitting costs of equipment" in the previous question, how much (**IN PESOS**) do you think will it cost?

- ◯ 1 to 200,000
- ◯ 200,001 to 400,000
- ◯ 400,001 to 600,000
- ◯ 600,001 to 800,000
- ◯ 800,001 to 1,000,000
- ◯ 1,000,001 and above

* 124. Are you open to switching to **WIND** in you production processes, self-generation and back-up generation of power?

- ◯ Yes
- ◯ No

V. Aptitude on Alternative Fuels and Primary Energies - Wind

* 125. In case you decide to switch to **WIND** in your **PRODUCTION PROCESSES**, which among the following fuels would you most likely replace it with? Please check all that apply.

- ☐ Biodiesel
- ☐ Bunker
- ☐ Coal
- ☐ Diesel
- ☐ Gasoline
- ☐ Kerosene
- ☐ LPG
- ☐ Propane
- ☐ Other (please specify)

* 126. In case you decide to switch to **WIND** in **SELF-GENERATION OF POWER**, which among the following fuels and primary energies would you most likely replace it with? Please check all that apply.

- ☐ Biodiesel
- ☐ Bunker
- ☐ Coal
- ☐ Diesel
- ☐ Gasoline
- ☐ Kerosene
- ☐ LPG
- ☐ Natural gas
- ☐ Propane
- ☐ Solar
- ☐ Other (please specify)

* 127. In case you decide to switch to **WIND** in **BACK-UP POWER GENERATION**, which among the following fuels and primary energies would you most likely replace it with? Please check all that apply.

- [ ] Biodiesel
- [ ] Bunker
- [ ] Coal
- [ ] Diesel
- [ ] Gasoline
- [ ] Kerosene
- [ ] LPG
- [ ] Natural gas
- [ ] Propane
- [ ] Solar
- [ ] Other (please specify)

____

* 128. In case you decide to switch to **WIND**, what would be your requirement **IN KILOWATT-HOURS?**

Conversion:
1 megawatt-hour = 1,000 kilowatt-hours

- ◯ 1 to 10,000
- ◯ 10,001 to 20,000
- ◯ 20,001 to 30,000
- ◯ 30,001 to 40,000
- ◯ 40,001 to 50,000
- ◯ 50,001 and above

V. Aptitude on Alternative Fuels and Primary Energies - Wind

* 129. Do you have a parent or partner company elsewhere that has any experiences using **WIND** as primary energy?

- ◯ Yes
- ◯ No

VI. Profile of Other Respondents

130. Are there any other person/s who helped you in answering this survey? If yes, please answer below.

Name of other respondent (1)	
Position of other respondent (1)	
Name of other respondent (2)	
Position of other respondent (2)	
Name of other respondent (3)	
Position of other respondent (3)	
