# Supplementary material for: Survey data on energy and fuel use of firms in economic zones in the Philippines
Source: Data Brief. 2021 Nov 26;39:107637. doi: 10.1016/j.dib.2021.107637 (PMC8645435; doi:10.1016/j.dib.2021.107637)
Supplement: Supplementary file 1 — Appendix 1 Anonymized raw data (DIB Energy Ravago et al 2021_Data.csv) Appendix 2 Data dictionary (DIB Energy Ravago et al 2021_Dictionary.txt) Appendix 3 FGD Survey questionnaire (DIB Energy Ravago et al 2021_FGD questionnaire.pdf) Appendix 4 Survey questionnaire (DIB Energy Ravago et al 2021_Survey questionaire.pdf) Appendix 5 General results from the survey (DIB Energy Ravago et al 2021_General results.pdf) [file mmc1.zip › Supplementary Appendix 4 DIB Energy Ravago et al 2021_ Survey Questionnaire.pdf]

Survey Data on Energy and Fuel Use of Firms in Economic Zones in the Philippines
Supplementary Appendix 4: Survey questionnaire

Authors

Majah-Leah Ravago[1], Raul Fabella[2], Karl Robert Jandoc[2], Renzi Frias[3,4], J. Kathleen Magadia[4]

Affiliations

1. Department of Economics, Ateneo de Manila University, Room 409 Leong Hall, Katipunan Avenue, Loyola Heights, 1108, Quezon City, Philippines
2. School of Economics, University of the Philippines, Guerrero corner Osmeña Streets, Diliman, 1101, Quezon City, Philippines
3. School of Statistics, University of the Philippines, T. M. Kalaw Street, Diliman, 1101, Quezon City, Philippines
4. Gas Policy Development Project, UP Statistical Center Research Foundation, Inc., School of Statistics, University of the Philippines, Quirino Avenue Kalaw Street, Diliman, 1101, Quezon City, Philippines

Corresponding author

Majah-Leah Ravago (mravago@ateneo.edu)

	SECTION	DESCRIPTION	PAGE
	—	About the survey; personal data; administrator contact information; privacy notice including statements on personal data collection, methods of processing, purposes of collection, information on personal information controller, data sharing and disclosure to third parties, confidentiality, and contact information; general instructions; overview of sections	1 - 5
I	**General Information**	Ecozone and firm's information; personnel; book value	6 - 11
II	**Production Schedule and Operation**	Production sales; peak and low month schedule and operation	12 - 23
III	**Utilities**	Electricity sources, requirements, uses, and considerations; electricity and water consumption and expenditure; energy conservation	24 - 51
IV	**Fuels Used in Production**	Production fuel mix; importance, use, consumption, and expenditure on different types of fuel (biodiesel, bunker, coal, diesel, gasoline, kerosene, LPG, natural gas, propane, other fuels) in main production processes	52 - 146
V	**Aptitude on Alternative Fuels and Primary Energies**	Knowledge, considerations, and opinions on alternative fuels and primary energies (natural gas, solar, wind), and experiences in using them	147 - 165
VI	**Other Questions**	Business expansion considerations	166
VII	**Respondent Information**	Primary and secondary respondent information	167 - 169
	—	Project information	170

Survey for Philippine Special Economic Zones

About the survey

On average, this survey will take **30 minutes** to finish.

This research survey is being undertaken by the UP Statistical Center Research Foundation, Inc. - Gas Policy Development Project (UPSCRFI-GPDP), the Philippine Economic Zone Authority (PEZA), and the Department of Energy (DOE) to establish baseline information and gather market profile of manufacturing and agro-industrial Philippine Special Economic Zones.

Respondent companies were chosen based on the nature of the economic zone where the company is in. Participation in this survey is **free and optional/voluntary**, and completion will not entail any monetary incentive.

Personal data

This survey will ask for the name and position of the primary respondent, work telephone number, and work email address. These personal data will only be used to contact you for any clarification two weeks after the survey. After validation, these information will be permanently removed by deleting the corresponding questions in the survey database and the associated responses. Deleting these questions will delete all responses associated with them.

Note: Providing us these personal data is optional/voluntary.

Administrator contact information

If you have questions or concerns regarding the survey and your participation in it, you may contact survey administrators **Mr. Renzi Frias** or **Ms. J.Kathleen Magadia** at **981-8500 local 3509** or **infogpdp.ph@gmail.com**.

If the survey administrators cannot be reached or you want to talk to someone other than those working on the study, you may contact the **University Research Ethics Office, Ateneo de Manila University** at **(632) 426-4001 local 4030** for any question, concern, or complaint about your rights as a research subject.

Privacy Notice

The UP Statistical Center Research Foundation, Inc. – Gas Policy Development Project (“UPSCRFI-GPDP”) values and protects your data privacy rights under the Data Privacy Act of 2012 (“DPA”) and its implementing rules and regulations (“IRR”).

This Privacy Notice states how UPSCRFI-GPDP processes your Personal Data as defined below. UPSCRFI-GPDP reserves the right to update or amend this Privacy Notice at any time.

What Personal Data we collect

“Personal Data” refers to personal information, sensitive personal information and privileged information which we collect from you, including but not limited to your:

1. Name
2. Position in the company
3. Work/office telephone number
4. Work/office e-mail address

You are responsible for ensuring that all Personal Data which you provide UPSCRFI-GPDP is complete, accurate, and up to date.

Collection of Personal Data

UPSCRFI-GPDP will collect Personal Data when you participate in any of the research projects conducted by UPSCRFI-GPDP by itself or those with partners.

UPSCRFI-GPDP does not knowingly collect information which are unnecessary for the purposes herein set forth.

Methods of processing

Your Personal Data may be processed both by way of computer media and on paper, in compliance with the rules in relation to personal data protection, therein including those relating to data security.

Our purposes for collecting and processing Personal Data

We collect and may process your Personal Data for the following purposes:

a. To use as the basis for the research project;
b. To contact you for clarification or verification of the information provided;

The information on Personal Data will be detachable for ease of purge once survey responses are validated to be complete and clear. After validation, these Personal Data will be permanently removed from the survey database.

Note: Providing us these Personal Data is optional/voluntary.

Personal Information Controller

Majah-Leah Ravago, Raul Fabella, and Karl Robert Jandoc are the Personal Information Controllers (PICs) under the DPA, which means that they determine what purposes personal information they hold will be used for.

UPSCRFI-GPDP is the Personal Information Processor, which means that the PICs are part of a project that process Personal Data.

Data sharing and disclosure to third parties

UPSCRFI-GPDP may share and/or disclose your Personal Data for the purposes indicated above with the Government and its agencies;

As a “data subject” under the DPA and its IRR, you have the following rights:

1. **Right to be Informed** - the right to know that your Personal Data has been processed, is being processed, and will be processed;
2. **Right to Object** - the right to object to the processing of your Personal Data;
3. **Right to Access** - the right to be given reasonable access to your Personal Data that has been processed and the manner by which it is processed;
4. **Right to Rectification** - the right to have Personal Data updated or any errors in your Personal Data corrected;
5. **Right to Erasure or Blocking** - the right to ask for suspension, withdrawal, blocking, or removal of your Personal Data from our records, unless this is required for your participation in the research project or is mandated by law to be retained for a lawful period of time;
6. **Right to Damages** - the right to damages if it is proven that your rights under the DPA and its IRR have been violated; and
7. **Right to File a Complaint** - the right to complain the National Privacy Commission if your rights under the DPA have been violated.

Confidentiality of Personal Data

Your Personal Data will be kept strictly confidential, and we have implemented organizational, physical, and technical security measures, policies and procedures intended to reduce the risks of accidental destruction or loss, or the unauthorized disclosure or access to Personal Data which we collect and store in our systems. UPSCRFI-GPDP nor its research partners will not identify you or your organization in any reports, publications or presentations. You understand that other researchers/third party may have access to this data only if they agree to preserve the confidentiality of the data and abide by UPSCRFI-GPDP's or the research partner’s data privacy policy.

Contact us

If you have any questions regarding the processing of your Personal Data or wish to exercise any of your rights as a data subject, you may contact the UPSCRFI-GPDP at **981-8500 local 3509** or **infogpdp.ph@gmail.com**.

By clicking '**Next**', you agree to participate in the survey.

Instructions

1. This survey will ask for details on **company/production processes and finances** which may need the assistance of officials knowledgeable on them. It is recommended that one or more of the following officials (or similar officials) answer the survey **together in one seating**.

- Production engineer/s or officer/s
- Finance manager/s or officer/s
- Human resource officer/s
- Pollution control officer/s
- Environment, safety, and health head or officer/s
- Facilities/equipment head or officer/s
- Utility head or officer/s

2. This survey may only be answered **once from the same computer or device**. You may change your answers and go back to particular pages before clicking the 'Submit' button at the end of the survey.

3. Kindly prepare your water and electricity bills for the **past 3 months** and use them as references for the questions on utility consumption and expenditure.

Sections

I. General information
II. Production schedule and operation
III. Utilities
IV. Fuels used in production
V. Aptitude on alternative fuels and primary energies
VI. Other questions
VII. Respondent information

I. General Information

In this section, you will be asked about basic company information.

I. General Information

* 1. What is the name of your ecozone?

* 2. Company information

Name of company

Street address

City or municipality

Province

ZIP code

I. General Information - Personnel

* 3. How many personnel does your company have?

* 4. How much (in **PERCENT**) of the total number of personnel is each category? Sum must be equal to 100.

Administrative and support

Production and technical

* 5. How much is your **monthly** total expense (in **PESOS**) for the salary of **all personnel**? Do not include peso sign, decimal point, and decimal numbers.

* 6. How much (in **PERCENT**) of the **monthly** total expense for the salary of **all personnel** is allotted per each category? Sum must be equal to 100.

Administrative and support

Production and technical

I. General Information - Book Value

* 7. What is the estimated book value (in **PESOS**) of your tangible fixed assets as of **December 2018**?

Tangible fixed assets *refer to physical assets required and for use of the company and is expected to have a productive life of more than one year. They include land, buildings, other structure and land improvements, transport equipment such as cars, trucks, aircrafts, and ships, machinery and equipment, valuables such as paintings and sculptures, and other tangible fixed assets such as fixtures and furnitures.*

Book value *refers to the initial or acquisition cost of tangible fixed assets less accumulated depreciation charges.*

- ◯ 1 billion and below
- ◯ Above 1 billion

I. General Information - Book Value

* 8. More specifically, in which of the following intervals does the estimated book value (in **PESOS**) of your tangible fixed assets belong?

- ◯ 200 million and below
- ◯ 200,000,001 to 400 million
- ◯ 400,000,001 to 600 million
- ◯ 600,000,001 to 800 million
- ◯ 800,000,001 to 1 billion

I. General Information - Book Value

* 9. More specifically, in which of the following intervals does the estimated book value (in **PESOS**) of your tangible fixed assets belong?

- ◯ 20 billion and below
- ◯ 20,000,000,001 to 40 billion
- ◯ 40,000,000,001 to 60 billion
- ◯ 60,000,000,001 to 80 billion
- ◯ 80,000,000,001 to 100 billion
- ◯ 100,000,000,001 and above

II. Production Schedule and Operation

In this section, you will be asked about production sales, schedules, and operations.

II. Production Schedule and Operation - Sales

* 10. Are your products sold domestically or exported?

- ◯ Sold domestically
- ◯ Exported
- ◯ Both

* 11. How much was your annual production sales (in **PESOS**) in **2018**?

- ◯ 1 billion and below
- ◯ Above 1 billion

II. Production Schedule and Operation - Sales

* 12. More specifically, in which of the following intervals do your 2018 annual production sales (in **PESOS**) belong?

- ◯ 200 million and below
- ◯ 200,000,001 to 400 million
- ◯ 400,000,001 to 600 million
- ◯ 600,000,001 to 800 million
- ◯ 800,000,001 to 1 billion

II. Production Schedule and Operation - Sales

* 13. More specifically, in which of the following intervals do your 2018 annual production sales (in **PESOS**) belong?

- ◯ 20 billion and below
- ◯ 20,000,000,001 to 40 billion
- ◯ 40,000,000,001 to 60 billion
- ◯ 60,000,000,001 to 80 billion
- ◯ 80,000,000,001 to 100 billion
- ◯ 100,000,000,001 and above

II. Production Schedule and Operation - Peak Month

* 14. When was your **peak month** (i.e. month when production was highest) in **2018**?

- ◯ January
- ◯ February
- ◯ March
- ◯ April
- ◯ May
- ◯ June
- ◯ July
- ◯ August
- ◯ September
- ◯ October
- ◯ November
- ◯ December

* 15. During your **peak month**, how many **DAYS** did your **main production** equipment and facilities operate?

* 16. During your **peak month**, how many **HOURS PER DAY** did your **main production** facilities and equipment operate?

II. Production Schedule and Operation - Peak Month

* 17. During your **peak month**, how many **DAYS** did your **auxiliary** equipment and facilities (e.g. temperature/climate controllers, air conditioners, refrigerators, etc.) operate?

* 18. During your **peak month**, how many **HOURS PER DAY** did your **auxiliary** equipment and facilities (e.g. temperature/climate controllers, air conditioners, refrigerators, etc.) operate?

II. Production Schedule and Operation - Peak Month

* 19. During your **peak month**, did you spend for the distribution and delivery of your products?

◯ Yes

◯ No

II. Production Schedule and Operation - Peak Month

* 20. During your **peak month**, how much (in **PESOS**) did you spend for the distribution and delivery of your products?

- 50 million and below
- 50,000,001 to 100 million
- 100,000,001 to 150 million
- 150,000,001 to 200 million
- 200,000,001 to 250 million
- 250,000,001 and above

II. Production Schedule and Operation - Low Month

* 21. When was your **low month** (i.e. month when production was lowest) in **2018**?

- ◯ January
- ◯ February
- ◯ March
- ◯ April
- ◯ May
- ◯ June
- ◯ July
- ◯ August
- ◯ September
- ◯ October
- ◯ November
- ◯ December

* 22. During your **low month**, how many **DAYS** did your **main production** equipment and facilities operate?

* 23. During your **low month**, how many **HOURS PER DAY** did your **main production** facilities and equipment operate?

II. Production Schedule and Operation - Low Month

* 24. During your **low month**, how many **DAYS** did your **auxiliary** equipment and facilities (e.g. temperature/climate controllers, air conditioners, refrigerators, etc.) operate?

* 25. During your **low month**, how many **HOURS PER DAY** did your **auxiliary** equipment and facilities (e.g. temperature/climate controllers, air conditioners, refrigerators, etc.) operate?

II. Production Schedule and Operation - Low Month

* 26. During your **low month**, did you spend for the distribution and delivery of your products?

- ◯ Yes
- ◯ No

II. Production Schedule and Operation - Low Month

* 27. During your **low month**, how much (in **PESOS**) did you spend for the distribution and delivery of your products?

- 50 million and below
- 50,000,001 to 100 million
- 100,000,001 to 150 million
- 150,000,001 to 200 million
- 200,000,001 to 250 million
- 250,000,001 and above

III. Utilities

In this section, you will be asked about your electricity sources, requirements, uses, and conservation measures. You will also be asked about your water and electricity consumption and expenditure for the past 3 months.

III. Utilities - Electricity Sources

* 28. What are your **main sources** of electricity? Please check all that apply.

- [ ] Power plant inside ecozone
- [ ] Meralco or electric cooperative
- [ ] Retail electricity supplier (RES)
- [ ] Direct from generation company *(directly connected to NGCP)*
- [ ] Self-generation
- [ ] Other (please specify)

III. Utilities - Electricity Requirement

* 29. How much of your electricity requirement (in **PERCENT**) is supplied by each source? Sum must be equal to 100.

Power plant inside ecozone

Meralco or electric cooperative

Retail electricity supplier (RES)

Direct from generation company *(directly connected to NGCP)*

Self-generation

[Insert text from Other]

III. Utilities - Electricity Requirement

30. If you chose 'retail electricity supplier (RES)' in the question on electricity sources, where did you get your electricity supply before switching to RES?

* 31. After shifting to RES, have you switched retailers?

- ◯ Yes
- ◯ No

III. Utilities - Electricity Requirement

* 32. How many times have you switched?

III. Utilities - Self-generation

33. If you chose 'self-generation' in the question on electricity sources, which of the following fuels or primary energies do you use? Please check all that apply.

- [ ] Biodiesel
- [ ] Bunker
- [ ] Coal
- [ ] Diesel
- [ ] Gasoline
- [ ] Kerosene
- [ ] LPG
- [ ] Natural gas
- [ ] Propane
- [ ] Solar
- [ ] Wind
- [ ] Other (please specify)

III. Utilities - Back-up Generation

* 34. Do you have back-up generation unit/s?

- ◯ Yes
- ◯ No

III. Utilities - Back-up Generation

* 35. Which of the following fuels or primary energies do you use for back-up power generation? Please check all that apply.

- [ ] Biodiesel
- [ ] Bunker
- [ ] Coal
- [ ] Diesel
- [ ] Gasoline
- [ ] Kerosene
- [ ] LPG
- [ ] Natural gas
- [ ] Propane
- [ ] Solar
- [ ] Wind
- [ ] Other (please specify)

III. Utilities - Production Process using Electricity

* 36. Which of the following processes involved in your main production use **ELECTRICITY**? Please check all that apply.

- [ ] Air conditioning
- [ ] Air/gas mixing (e.g. Selas mixing, etc.)
- [ ] Baking
- [ ] Boiler operation (e.g. for steam generation, etc.)
- [ ] Burning
- [ ] Curing (e.g. oven curing, powder paint curing, etc.)
- [ ] Die casting or wire bonding
- [ ] Drying or annealing (e.g. oven drying, mold drying, core drying, air handling, etc.)
- [ ] Engine loading or preparation
- [ ] Fabrication
- [ ] Forklift operation
- [ ] Heat treatment
- [ ] Ice making
- [ ] Impregnation
- [ ] Machine injection or molding
- [ ] Melting or pre-melting
- [ ] Metal treatment or pre-treatment
- [ ] Painting
- [ ] Power generation
- [ ] Smelting
- [ ] Standby or back-up power generation
- [ ] Steel cutting
- [ ] Thermal oxidation
- [ ] Transportation and logistics (e.g. trucking, distribution, delivery, etc.)
- [ ] Welding
- [ ] Stamping
- [ ] Air compression / vacuuming
- [ ] Other (please specify)

____

III. Utilities - Electricity Consumption

For the next set of questions, kindly refer to your electricity bills.

* 37. For the past 3 months, what was your average **monthly electricity** consumption in **KILOWATT-HOURS**?

- ◯ 100,000 and below
- ◯ Above 100,000

III. Utilities - Electricity Consumption

* 38. More specifically, in which of the following intervals does your average **monthly electricity** consumption in **KILOWATT-HOURS** belong?

- ◯ 20,000 and below
- ◯ 20,001 to 40,000
- ◯ 40,001 to 60,000
- ◯ 60,001 to 80,000
- ◯ 80,001 to 100,000

III. Utilities - Electricity Consumption

* 39. More specifically, in which of the intervals does your average **monthly electricity** consumption in **KILOWATT-HOURS** belong?

- ◯ 200,000 and below
- ◯ 200,001 to 400,000
- ◯ 400,001 to 600,000
- ◯ 600,001 to 800,000
- ◯ 800,001 to 1 million
- ◯ 1,000,001 and above

III. Utilities - Electricity Expenditure

* 40. For the past 3 months, how much did you spend for **monthly electricity** in **PESOS**?

◯ 5 million and below

◯ Above 5 million

III. Utilities - Electricity Expenditure

* 41. More specifically, in which interval does your average **monthly electricity** expenditure in **PESOS** belong?

- ◯ 1 million and below
- ◯ 1,000,001 to 2 million
- ◯ 2,000,001 to 3 million
- ◯ 3,000,001 to 4 million
- ◯ 4,000,001 to 5 million

III. Utilities - Electricity Expenditure

* 42. More specifically, in which of the following intervals does your average **monthly electricity** expenditure in **PESOS** belong?

- ◯ 10 million and below
- ◯ 10,000,001 to 20 million
- ◯ 20,000,001 to 30 million
- ◯ 30,000,001 to 40 million
- ◯ 40,000,001 to 50 million
- ◯ 50,000,001 and above

III. Utilities - Electricity Expenditure

* 43. How much (in **PESOS**) do you pay **per kilowatt-hour** of electricity?

- ○ Less than 2.00
- ○ 2.01 to 4.00
- ○ 4.01 to 6.00
- ○ 6.01 to 8.00
- ○ 8.01 to 10.00
- ○ 10.01 to 12.00
- ○ 12.01 to 14.00
- ○ More than 14.00

III. Utilities - Water Consumption

For the next set of questions, kindly refer to your water bills.

* 44. For the past 3 months, what was your average **monthly water** consumption in **CUBIC METERS**?

○ 50,000 and below

○ Above 50,000

III. Utilities - Water Consumption

* 45. More specifically, in which of the following intervals does your average **monthly water** consumption in **CUBIC METERS** belong?

- ◯ 10,000 and below
- ◯ 10,001 to 20,000
- ◯ 20,001 to 30,000
- ◯ 30,001 to 40,000
- ◯ 40,001 to 50,000

III. Utilities - Water Consumption

* 46. More specifically, in which of the following intervals does your average **monthly water** consumption in **CUBIC METERS** belong?

- 100,000 and below
- 100,001 to 200,000
- 200,001 to 300,000
- 300,001 to 400,000
- 400,001 to 500,000
- 500,001 and above

III. Utilities - Water Expenditure

* 47. For the past 3 months, how much did you spend for **monthly water** in **PESOS**?

- ◯ 500,000 and below
- ◯ Above 500,000

III. Utilities - Water Expenditure

* 48. More specifically, in which of the following intervals does your average **monthly water** expenditure in **PESOS** belong?

- ◯ 100,000 and below
- ◯ 100,001 to 200,000
- ◯ 200,001 to 300,000
- ◯ 300,001 to 400,000
- ◯ 400,001 to 500,000

III. Utilities - Water Expenditure

* 49. More specifically, in which of the following intervals does your average **monthly water** expenditure in **PESOS** belong?

- ○ 1 million and below
- ○ 1,000,001 to 2 million
- ○ 2,000,001 to 3 million
- ○ 3,000,001 to 4 million
- ○ 4,000,001 to 5 million
- ○ 5,000,001 and above

III. Utilities - Water Expenditure

* 50. How much (in **PESOS**) do you pay **per cubic meter** of water?

- Less than 40.00
- 40.01 to 50.00
- 50.01 to 60.00
- 60.01 to 70.00
- 70.01 to 80.00
- 80.01 to 90.00
- 90.01 to 100.00
- More than 100.00

III. Utilities - Electricity Considerations

* 51. If you can switch to another electricity provider, what are your considerations? With 1 being the most important, rank the following.

You may choose a number from the dropdown, or drag and drop to reorder.

- Price
- Supply stability and reliability
- Safety and security
- Environmental concerns

III. Utilities - Energy Efficiency and Conservation

* 52. Have you participated in any PEZA-organized energy efficiency initiatives designed to improve operational efficiency and increase competitiveness?

- ◯ Yes
- ◯ No

* 53. Are you aware of any of the following?

	Yes	No
Energy management system (EnMS) standards (compliant with ISO 50001)	◯	◯
System optimization (SO) for steam, compressed air, and pumps	◯	◯
Financial opportunities for energy efficiency investments	◯	◯

* 54. Has your company implemented any measures to reduce energy consumption and cost?

- ◯ Yes
- ◯ No

III. Utilities - Energy Efficiency and Conservation

* 55. Which of the following measures has your company implemented to reduce energy consumption and cost? Please check all that apply.

- [ ] Installing solar panels
- [ ] Using inverter-type airconditioners
- [ ] Installing LED lighting
- [ ] Other (please specify)

III. Utilities - Energy Efficiency and Conservation

* 56. With 1 being the most important, rank the following reasons for not implementing or adopting any energy efficiency and conservation measures for operational efficiency.

You may choose a number from the dropdown, or drag and drop to reorder.

- Lack of understanding of the management on the advantages of adopting energy efficiency measures
- Lack of technical knowledge on how to implement energy efficient measures
- Lack of resources to switch to energy efficient equipment
- Energy efficiency not a company priority
- No perceived substantial effect in adopting energy efficient measures

IV. Fuels Used in Production

* 57. Do you use at least one of the following fuels in your production?

- Biodiesel
- Bunker
- Coal
- Diesel
- Gasoline
- Kerosene
- LPG
- Natural gas
- Propane
- Other fuels

◯ Yes

◯ No

* 58. What is your estimated production fuel mix? Write only the number. Input '0' if you do not use the fuel. Sum must be equal to 100.

Biodiesel	
Bunker	
Coal	
Diesel	
Electricity	
Gasoline	
Kerosene	
LPG	
Natural gas	
Propane	
Other fuel (1)	
Other fuel (2)	
Other fuel (3)	

IV. Fuels Used in Production

In this section, you will be asked about the importance, uses, consumption, and expenditure on different types of fuel (excluding electricity) in your main production processes. The different fuels are the following:

1. Biodiesel
2. Bunker
3. Coal
4. Diesel
5. Gasoline
6. Kerosene
7. LPG
8. Natural gas
9. Propane
10. Other fuels

IV. Fuels Used in Production - Biodiesel

* 59. Do you use **biodiesel** in your main production processes?

- ○ Yes
- ○ No

IV. Fuels Used in Production - Biodiesel

* 60. How important is **biodiesel** in your main production processes?

- ◯ Not important
- ◯ Slightly important
- ◯ Important
- ◯ Fairly important
- ◯ Very important

IV. Fuels Used in Production - Biodiesel

* 61. Which of the following processes involved in your main production use **biodiesel**? Please check all that apply.

- [ ] Air/gas mixing (e.g. Selas mixing, etc.)
- [ ] Baking
- [ ] Boiler operation (e.g. for steam generation, etc.)
- [ ] Burning
- [ ] Curing (e.g. oven curing, powder paint curing, etc.)
- [ ] Die casting or wire bonding
- [ ] Drying or annealing (e.g. oven drying, mold drying, core drying, air handling, etc.)
- [ ] Engine loading or preparation
- [ ] Fabrication
- [ ] Forklift operation
- [ ] Heat treatment
- [ ] Ice making
- [ ] Impregnation
- [ ] Machine injection or molding
- [ ] Melting or pre-melting
- [ ] Metal treatment or pre-treatment
- [ ] Painting
- [ ] Smelting
- [ ] Steel cutting
- [ ] Thermal oxidation
- [ ] Transportation and logistics (e.g. trucking, distribution, delivery, etc.)
- [ ] Welding
- [ ] Stamping
- [ ] Air compression/vacuuming
- [ ] Other (please specify)

IV. Fuels Used in Production - Biodiesel

* 62. How much **biodiesel** do you consume (in **LITERS**) for each process **PER DAY**?

	1 - 200	201 - 400	401 - 600	601 - 800	801 - 1,000	1,001 & up
Air/gas mixing (e.g. Selas mixing, etc.)	○	○	○	○	○	○
Baking	○	○	○	○	○	○
Boiler operation (e.g. for steam generation, etc.)	○	○	○	○	○	○
Burning	○	○	○	○	○	○
Curing (e.g. oven curing, powder paint curing, etc.)	○	○	○	○	○	○
Die casting or wire bonding	○	○	○	○	○	○
Drying or annealing (e.g. oven drying, mold drying, core drying, air handling, etc.)	○	○	○	○	○	○
Engine loading or preparation	○	○	○	○	○	○
Fabrication	○	○	○	○	○	○
Forklift operation	○	○	○	○	○	○
Heat treatment	○	○	○	○	○	○
Ice making	○	○	○	○	○	○
Impregnation	○	○	○	○	○	○
Machine injection or molding	○	○	○	○	○	○
Melting or pre-melting	○	○	○	○	○	○
Metal treatment or pre-treatment	○	○	○	○	○	○
Painting	○	○	○	○	○	○
Smelting	○	○	○	○	○	○
Welding	○	○	○	○	○	○
Stamping	○	○	○	○	○	○
Air compression/vacuuming	○	○	○	○	○	○
Steel cutting	○	○	○	○	○	○
Thermal oxidation	○	○	○	○	○	○
Transportation and logistics (e.g. trucking, distribution, delivery, etc.)	○	○	○	○	○	○
[Insert text from Other]	○	○	○	○	○	○

* 63. How much do you spend for your **biodiesel** consumption (in **PESOS**) for each process **PER MONTH**?

	1 - 270,000	270,001 - 540,000	540,001 - 810,000	810,001 - 1,080,000	1,080,001 - 1,350,000	1,350,001 & up
Air/gas mixing (e.g. Selas mixing, etc.)	◯	◯	◯	◯	◯	◯
Baking	◯	◯	◯	◯	◯	◯
Boiler operation (e.g. for steam generation, etc.)	◯	◯	◯	◯	◯	◯
Burning	◯	◯	◯	◯	◯	◯
Curing (e.g. oven curing, powder paint curing, etc.)	◯	◯	◯	◯	◯	◯
Die casting or wire bonding	◯	◯	◯	◯	◯	◯
Drying or annealing (e.g. oven drying, mold drying, core drying, air handling, etc.)	◯	◯	◯	◯	◯	◯
Engine loading or preparation	◯	◯	◯	◯	◯	◯
Fabrication	◯	◯	◯	◯	◯	◯
Forklift operation	◯	◯	◯	◯	◯	◯
Heat treatment	◯	◯	◯	◯	◯	◯
Ice making	◯	◯	◯	◯	◯	◯
Impregnation	◯	◯	◯	◯	◯	◯
Machine injection or molding	◯	◯	◯	◯	◯	◯
Melting or pre-melting	◯	◯	◯	◯	◯	◯
Metal treatment or pre-treatment	◯	◯	◯	◯	◯	◯
Painting	◯	◯	◯	◯	◯	◯
Smelting	◯	◯	◯	◯	◯	◯
Welding	◯	◯	◯	◯	◯	◯
Stamping	◯	◯	◯	◯	◯	◯
Air compression/vacuuming	◯	◯	◯	◯	◯	◯
Steel cutting	◯	◯	◯	◯	◯	◯
Thermal oxidation	◯	◯	◯	◯	◯	◯
Transportation and logistics (e.g. trucking, distribution, delivery, etc.)	◯	◯	◯	◯	◯	◯
[Insert text from Other]	◯	◯	◯	◯	◯	◯

* 64. How do you procure your **biodiesel**?

- Contracted
- Buying as needed
- Other (please specify)

IV. Fuels Used in Production - Bunker

* 65. Do you use **bunker** in your main production processes?

◯ Yes

◯ No

IV. Fuels Used in Production - Bunker

* 66. How important is **bunker** in your main production processes?

- ◯ Not important
- ◯ Slightly important
- ◯ Important
- ◯ Fairly important
- ◯ Very important

IV. Fuels Used in Production - Bunker

* 67. Which of the following processes involved in your main production use **bunker**? Please check all that apply.

- [ ] Air/gas mixing (e.g. Selas mixing, etc.)
- [ ] Baking
- [ ] Boiler operation (e.g. for steam generation, etc.)
- [ ] Burning
- [ ] Curing (e.g. oven curing, powder paint curing, etc.)
- [ ] Die casting or wire bonding
- [ ] Drying or annealing (e.g. oven drying, mold drying, core drying, air handling, etc.)
- [ ] Engine loading or preparation
- [ ] Fabrication
- [ ] Forklift operation
- [ ] Heat treatment
- [ ] Ice making
- [ ] Impregnation
- [ ] Machine injection or molding
- [ ] Melting or pre-melting
- [ ] Metal treatment or pre-treatment
- [ ] Painting
- [ ] Smelting
- [ ] Steel cutting
- [ ] Thermal oxidation
- [ ] Transportation and logistics (e.g. trucking, distribution, delivery, etc.)
- [ ] Welding
- [ ] Stamping
- [ ] Air compression/vacuuming
- [ ] Other (please specify)

IV. Fuels Used in Production - Bunker

* 68. How much **bunker** do you consume (in **LITERS**) for each process **PER DAY**?

	1 - 200	201 - 400	401 - 600	601 - 800	801 - 1,000	1,001 & up
Air/gas mixing (e.g. Selas mixing, etc.)						
Baking						
Boiler operation (e.g. for steam generation, etc.)						
Burning						
Curing (e.g. oven curing, powder paint curing, etc.)						
Die casting or wire bonding						
Drying or annealing (e.g. oven drying, mold drying, core drying, air handling, etc.)						
Engine loading or preparation						
Fabrication						
Forklift operation						
Heat treatment						
Ice making						
Impregnation						
Machine injection or molding						
Melting or pre-melting						
Metal treatment or pre-treatment						
Painting						
Smelting						
Welding						
Stamping						
Air compression/vacuuming						
Steel cutting						
Thermal oxidation						
Transportation and logistics (e.g. trucking, distribution, delivery, etc.)						
[Insert text from Other]						

* 69. How much do you spend for your **bunker** consumption (in **PESOS**) for each process **PER MONTH**?

	1 - 270,000	270,001 - 540,000	540,001 - 810,000	810,001 - 1,080,000	1,080,001 - 1,350,000	1,350,001 & up
Air/gas mixing (e.g. Selas mixing, etc.)	○	○	○	○	○	○
Baking	○	○	○	○	○	○
Boiler operation (e.g. for steam generation, etc.)	○	○	○	○	○	○
Burning	○	○	○	○	○	○
Curing (e.g. oven curing, powder paint curing, etc.)	○	○	○	○	○	○
Die casting or wire bonding	○	○	○	○	○	○
Drying or annealing (e.g. oven drying, mold drying, core drying, air handling, etc.)	○	○	○	○	○	○
Engine loading or preparation	○	○	○	○	○	○
Fabrication	○	○	○	○	○	○
Forklift operation	○	○	○	○	○	○
Heat treatment	○	○	○	○	○	○
Ice making	○	○	○	○	○	○
Impregnation	○	○	○	○	○	○
Machine injection or molding	○	○	○	○	○	○
Melting or pre-melting	○	○	○	○	○	○
Metal treatment or pre-treatment	○	○	○	○	○	○
Painting	○	○	○	○	○	○
Smelting	○	○	○	○	○	○
Welding	○	○	○	○	○	○
Stamping	○	○	○	○	○	○
Air compression/vacuuming	○	○	○	○	○	○
Steel cutting	○	○	○	○	○	○
Thermal oxidation	○	○	○	○	○	○
Transportation and logistics (e.g. trucking, distribution, delivery, etc.)	○	○	○	○	○	○
[Insert text from Other]	○	○	○	○	○	○

* 70. How do you procure your **bunker**?

- ○ Contracted
- ○ Buying as needed
- ○ Other (please specify)

IV. Fuels Used in Production - Coal

* 71. Do you use **coal** in your main production processes?

◯ Yes

◯ No

IV. Fuels Used in Production - Coal

* 72. How important is **coal** in your main production processes?

- Not important
- Slightly important
- Important
- Fairly important
- Very important

IV. Fuels Used in Production - Coal

* 73. Which of the following processes involved in your main production use **coal**? Please check all that apply.

- [ ] Air/gas mixing (e.g. Selas mixing, etc.)
- [ ] Baking
- [ ] Boiler operation (e.g. for steam generation, etc.)
- [ ] Burning
- [ ] Curing (e.g. oven curing, powder paint curing, etc.)
- [ ] Die casting or wire bonding
- [ ] Drying or annealing (e.g. oven drying, mold drying, core drying, air handling, etc.)
- [ ] Engine loading or preparation
- [ ] Fabrication
- [ ] Forklift operation
- [ ] Heat treatment
- [ ] Ice making
- [ ] Impregnation
- [ ] Machine injection or molding
- [ ] Melting or pre-melting
- [ ] Metal treatment or pre-treatment
- [ ] Painting
- [ ] Smelting
- [ ] Steel cutting
- [ ] Thermal oxidation
- [ ] Transportation and logistics (e.g. trucking, distribution, delivery, etc.)
- [ ] Welding
- [ ] Stamping
- [ ] Air compression/vacuuming
- [ ] Other (please specify)

IV. Fuels Used in Production - Coal

* 74. How much **coal** do you consume (in **METRIC TONS**) for each process **PER DAY**?

	1 - 20	21 - 40	41 - 60	61 - 80	81 - 100	101 & up
Air/gas mixing (e.g. Selas mixing, etc.)	○	○	○	○	○	○
Baking	○	○	○	○	○	○
Boiler operation (e.g. for steam generation, etc.)	○	○	○	○	○	○
Burning	○	○	○	○	○	○
Curing (e.g. oven curing, powder paint curing, etc.)	○	○	○	○	○	○
Die casting or wire bonding	○	○	○	○	○	○
Drying or annealing (e.g. oven drying, mold drying, core drying, air handling, etc.)	○	○	○	○	○	○
Engine loading or preparation	○	○	○	○	○	○
Fabrication	○	○	○	○	○	○
Forklift operation	○	○	○	○	○	○
Heat treatment	○	○	○	○	○	○
Ice making	○	○	○	○	○	○
Impregnation	○	○	○	○	○	○
Machine injection or molding	○	○	○	○	○	○
Melting or pre-melting	○	○	○	○	○	○
Metal treatment or pre-treatment	○	○	○	○	○	○
Painting	○	○	○	○	○	○
Smelting	○	○	○	○	○	○
Welding	○	○	○	○	○	○
Stamping	○	○	○	○	○	○
Air compression/vacuuming	○	○	○	○	○	○
Steel cutting	○	○	○	○	○	○
Thermal oxidation	○	○	○	○	○	○
Transportation and logistics (e.g. trucking, distribution, delivery, etc.)	○	○	○	○	○	○
[Insert text from Other]	○	○	○	○	○	○

* 75. How much do you spend for your **coal** consumption (in **PESOS**) for each process **PER MONTH**?

	1 - 3,000,000	3,000,001 - 6,000,000	6,000,001 - 9,000,000	9,000,001 - 12,000,000	12,000,001 - 15,000,000	15,000,001 & up
Air/gas mixing (e.g. Selas mixing, etc.)	○	○	○	○	○	○
Baking	○	○	○	○	○	○
Boiler operation (e.g. for steam generation, etc.)	○	○	○	○	○	○
Burning	○	○	○	○	○	○
Curing (e.g. oven curing, powder paint curing, etc.)	○	○	○	○	○	○
Die casting or wire bonding	○	○	○	○	○	○
Drying or annealing (e.g. oven drying, mold drying, core drying, air handling, etc.)	○	○	○	○	○	○
Engine loading or preparation	○	○	○	○	○	○
Fabrication	○	○	○	○	○	○
Forklift operation	○	○	○	○	○	○
Heat treatment	○	○	○	○	○	○
Ice making	○	○	○	○	○	○
Impregnation	○	○	○	○	○	○
Machine injection or molding	○	○	○	○	○	○
Melting or pre-melting	○	○	○	○	○	○
Metal treatment or pre-treatment	○	○	○	○	○	○
Painting	○	○	○	○	○	○
Smelting	○	○	○	○	○	○
Welding	○	○	○	○	○	○
Stamping	○	○	○	○	○	○
Air compression/vacuuming	○	○	○	○	○	○
Steel cutting	○	○	○	○	○	○
Thermal oxidation	○	○	○	○	○	○
Transportation and logistics (e.g. trucking, distribution, delivery, etc.)	○	○	○	○	○	○
[Insert text from Other]	○	○	○	○	○	○

* 76. How do you procure your **coal**?

- ◯ Contracted
- ◯ Buying as needed
- ◯ Other (please specify)

IV. Fuels Used in Production - Diesel

* 77. Do you use **diesel** in your main production processes?

◯ Yes

◯ No

IV. Fuels Used in Production - Diesel

* 78. How important is **diesel** in your main production processes?

- ◯ Not important
- ◯ Slightly important
- ◯ Important
- ◯ Fairly important
- ◯ Very important

IV. Fuels Used in Production - Diesel

* 79. Which of the following processes involved in your main production use **diesel**? Please check all that apply.

- [ ] Air/gas mixing (e.g. Selas mixing, etc.)
- [ ] Baking
- [ ] Boiler operation (e.g. for steam generation, etc.)
- [ ] Burning
- [ ] Curing (e.g. oven curing, powder paint curing, etc.)
- [ ] Die casting or wire bonding
- [ ] Drying or annealing (e.g. oven drying, mold drying, core drying, air handling, etc.)
- [ ] Engine loading or preparation
- [ ] Fabrication
- [ ] Forklift operation
- [ ] Heat treatment
- [ ] Ice making
- [ ] Impregnation
- [ ] Machine injection or molding
- [ ] Melting or pre-melting
- [ ] Metal treatment or pre-treatment
- [ ] Painting
- [ ] Smelting
- [ ] Steel cutting
- [ ] Thermal oxidation
- [ ] Transportation and logistics (e.g. trucking, distribution, delivery, etc.)
- [ ] Welding
- [ ] Stamping
- [ ] Air compression/vacuuming
- [ ] Other (please specify)

IV. Fuels Used in Production - Diesel

* 80. How much **diesel** do you consume (in **LITERS**) for each process **PER DAY**?

	1 - 200	201 - 400	401 - 600	601 - 800	801 - 1,000	1,001 & up
Air/gas mixing (e.g. Selas mixing, etc.)	○	○	○	○	○	○
Baking	○	○	○	○	○	○
Boiler operation (e.g. for steam generation, etc.)	○	○	○	○	○	○
Burning	○	○	○	○	○	○
Curing (e.g. oven curing, powder paint curing, etc.)	○	○	○	○	○	○
Die casting or wire bonding	○	○	○	○	○	○
Drying or annealing (e.g. oven drying, mold drying, core drying, air handling, etc.)	○	○	○	○	○	○
Engine loading or preparation	○	○	○	○	○	○
Fabrication	○	○	○	○	○	○
Forklift operation	○	○	○	○	○	○
Heat treatment	○	○	○	○	○	○
Ice making	○	○	○	○	○	○
Impregnation	○	○	○	○	○	○
Machine injection or molding	○	○	○	○	○	○
Melting or pre-melting	○	○	○	○	○	○
Metal treatment or pre-treatment	○	○	○	○	○	○
Painting	○	○	○	○	○	○
Smelting	○	○	○	○	○	○
Welding	○	○	○	○	○	○
Stamping	○	○	○	○	○	○
Air compression/vacuuming	○	○	○	○	○	○
Steel cutting	○	○	○	○	○	○
Thermal oxidation	○	○	○	○	○	○
Transportation and logistics (e.g. trucking, distribution, delivery, etc.)	○	○	○	○	○	○
[Insert text from Other]	○	○	○	○	○	○

* 81. How much do you spend for your **diesel** consumption (in **PESOS**) for each process **PER MONTH**?

	1 - 270,000	270,001 - 540,000	540,001 - 810,000	810,001 - 1,080,000	1,080,001 - 1,350,000	1,350,001 & up
Air/gas mixing (e.g. Selas mixing, etc.)	○	○	○	○	○	○
Baking	○	○	○	○	○	○
Boiler operation (e.g. for steam generation, etc.)	○	○	○	○	○	○
Burning	○	○	○	○	○	○
Curing (e.g. oven curing, powder paint curing, etc.)	○	○	○	○	○	○
Die casting or wire bonding	○	○	○	○	○	○
Drying or annealing (e.g. oven drying, mold drying, core drying, air handling, etc.)	○	○	○	○	○	○
Engine loading or preparation	○	○	○	○	○	○
Fabrication	○	○	○	○	○	○
Forklift operation	○	○	○	○	○	○
Heat treatment	○	○	○	○	○	○
Ice making	○	○	○	○	○	○
Impregnation	○	○	○	○	○	○
Machine injection or molding	○	○	○	○	○	○
Melting or pre-melting	○	○	○	○	○	○
Metal treatment or pre-treatment	○	○	○	○	○	○
Painting	○	○	○	○	○	○
Smelting	○	○	○	○	○	○
Welding	○	○	○	○	○	○
Stamping	○	○	○	○	○	○
Air compression/vacuuming	○	○	○	○	○	○
Steel cutting	○	○	○	○	○	○
Thermal oxidation	○	○	○	○	○	○
Transportation and logistics (e.g. trucking, distribution, delivery, etc.)	○	○	○	○	○	○
[Insert text from Other]	○	○	○	○	○	○

* 82. How do you procure your **diesel**?

- ◯ Contracted
- ◯ Buying as needed
- ◯ Other (please specify)

IV. Fuels Used in Production - Gasoline

* 83. Do you use **gasoline** in your main production processes?

- ◯ Yes
- ◯ No

IV. Fuels Used in Production - Gasoline

* 84. How important is **gasoline** in your main production processes?

- ◯ Not important
- ◯ Slightly important
- ◯ Important
- ◯ Fairly important
- ◯ Very important

IV. Fuels Used in Production - Gasoline

* 85. Which of the following processes involved in your main production use **gasoline**? Please check all that apply.

- [ ] Air/gas mixing (e.g. Selas mixing, etc.)
- [ ] Baking
- [ ] Boiler operation (e.g. for steam generation, etc.)
- [ ] Burning
- [ ] Curing (e.g. oven curing, powder paint curing, etc.)
- [ ] Die casting or wire bonding
- [ ] Drying or annealing (e.g. oven drying, mold drying, core drying, air handling, etc.)
- [ ] Engine loading or preparation
- [ ] Fabrication
- [ ] Forklift operation
- [ ] Heat treatment
- [ ] Ice making
- [ ] Impregnation
- [ ] Machine injection or molding
- [ ] Melting or pre-melting
- [ ] Metal treatment or pre-treatment
- [ ] Painting
- [ ] Smelting
- [ ] Steel cutting
- [ ] Thermal oxidation
- [ ] Transportation and logistics (e.g. trucking, distribution, delivery, etc.)
- [ ] Welding
- [ ] Stamping
- [ ] Air compression/vacuuming
- [ ] Other (please specify)

IV. Fuels Used in Production - Gasoline

* 86. How much **gasoline** do you consume (in **LITERS**) for each process **PER DAY**?

	1 - 200	201 - 400	401 - 600	601 - 800	801 - 1,000	1,001 & up
Air/gas mixing (e.g. Selas mixing, etc.)	○	○	○	○	○	○
Baking	○	○	○	○	○	○
Boiler operation (e.g. for steam generation, etc.)	○	○	○	○	○	○
Burning	○	○	○	○	○	○
Curing (e.g. oven curing, powder paint curing, etc.)	○	○	○	○	○	○
Die casting or wire bonding	○	○	○	○	○	○
Drying or annealing (e.g. oven drying, mold drying, core drying, air handling, etc.)	○	○	○	○	○	○
Engine loading or preparation	○	○	○	○	○	○
Fabrication	○	○	○	○	○	○
Forklift operation	○	○	○	○	○	○
Heat treatment	○	○	○	○	○	○
Ice making	○	○	○	○	○	○
Impregnation	○	○	○	○	○	○
Machine injection or molding	○	○	○	○	○	○
Melting or pre-melting	○	○	○	○	○	○
Metal treatment or pre-treatment	○	○	○	○	○	○
Painting	○	○	○	○	○	○
Smelting	○	○	○	○	○	○
Welding	○	○	○	○	○	○
Stamping	○	○	○	○	○	○
Air compression/vacuuming	○	○	○	○	○	○
Steel cutting	○	○	○	○	○	○
Thermal oxidation	○	○	○	○	○	○
Transportation and logistics (e.g. trucking, distribution, delivery, etc.)	○	○	○	○	○	○
[Insert text from Other]	○	○	○	○	○	○

* 87. How much do you spend for your **gasoline** consumption (in **PESOS**) for each process **PER MONTH**?

	1 - 360,000	360,001 - 720,000	720,001 - 1,080,000	1,080,001 - 1,440,000	1,440,001 - 1,800,000	1,800,001 & up
Air/gas mixing (e.g. Selas mixing, etc.)	○	○	○	○	○	○
Baking	○	○	○	○	○	○
Boiler operation (e.g. for steam generation, etc.)	○	○	○	○	○	○
Burning	○	○	○	○	○	○
Curing (e.g. oven curing, powder paint curing, etc.)	○	○	○	○	○	○
Die casting or wire bonding	○	○	○	○	○	○
Drying or annealing (e.g. oven drying, mold drying, core drying, air handling, etc.)	○	○	○	○	○	○
Engine loading or preparation	○	○	○	○	○	○
Fabrication	○	○	○	○	○	○
Forklift operation	○	○	○	○	○	○
Heat treatment	○	○	○	○	○	○
Ice making	○	○	○	○	○	○
Impregnation	○	○	○	○	○	○
Machine injection or molding	○	○	○	○	○	○
Melting or pre-melting	○	○	○	○	○	○
Metal treatment or pre-treatment	○	○	○	○	○	○
Painting	○	○	○	○	○	○
Smelting	○	○	○	○	○	○
Welding	○	○	○	○	○	○
Stamping	○	○	○	○	○	○
Air compression/vacuuming	○	○	○	○	○	○
Steel cutting	○	○	○	○	○	○
Thermal oxidation	○	○	○	○	○	○
Transportation and logistics (e.g. trucking, distribution, delivery, etc.)	○	○	○	○	○	○
[Insert text from Other]	○	○	○	○	○	○

* 88. How do you procure your **gasoline**?

- ◯ Contracted
- ◯ Buying as needed
- ◯ Other (please specify)

IV. Fuels Used in Production - Kerosene

* 89. Do you use **kerosene** in your main production processes?

◯ Yes

◯ No

IV. Fuels Used in Production - Kerosene

* 90. How important is **kerosene** in your main production processes?

- ◯ Not important
- ◯ Slightly important
- ◯ Important
- ◯ Fairly important
- ◯ Very important

IV. Fuels Used in Production - Kerosene

* 91. Which of the following processes involved in your main production use **kerosene**? Please check all that apply.

- [ ] Air/gas mixing (e.g. Selas mixing, etc.)
- [ ] Baking
- [ ] Boiler operation (e.g. for steam generation, etc.)
- [ ] Burning
- [ ] Curing (e.g. oven curing, powder paint curing, etc.)
- [ ] Die casting or wire bonding
- [ ] Drying or annealing (e.g. oven drying, mold drying, core drying, air handling, etc.)
- [ ] Engine loading or preparation
- [ ] Fabrication
- [ ] Forklift operation
- [ ] Heat treatment
- [ ] Ice making
- [ ] Impregnation
- [ ] Machine injection or molding
- [ ] Melting or pre-melting
- [ ] Metal treatment or pre-treatment
- [ ] Painting
- [ ] Smelting
- [ ] Steel cutting
- [ ] Thermal oxidation
- [ ] Transportation and logistics (e.g. trucking, distribution, delivery, etc.)
- [ ] Welding
- [ ] Stamping
- [ ] Air compression/vacuuming
- [ ] Other (please specify)

IV. Fuels Used in Production - Kerosene

* 92. How much **kerosene** do you consume (in **LITERS**) for each process **PER DAY**?

	1 - 200	201 - 400	401 - 600	601 - 800	801 - 1,000	1,001 & up
Air/gas mixing (e.g. Selas mixing, etc.)	○	○	○	○	○	○
Baking	○	○	○	○	○	○
Boiler operation (e.g. for steam generation, etc.)	○	○	○	○	○	○
Burning	○	○	○	○	○	○
Curing (e.g. oven curing, powder paint curing, etc.)	○	○	○	○	○	○
Die casting or wire bonding	○	○	○	○	○	○
Drying or annealing (e.g. oven drying, mold drying, core drying, air handling, etc.)	○	○	○	○	○	○
Engine loading or preparation	○	○	○	○	○	○
Fabrication	○	○	○	○	○	○
Forklift operation	○	○	○	○	○	○
Heat treatment	○	○	○	○	○	○
Ice making	○	○	○	○	○	○
Impregnation	○	○	○	○	○	○
Machine injection or molding	○	○	○	○	○	○
Melting or pre-melting	○	○	○	○	○	○
Metal treatment or pre-treatment	○	○	○	○	○	○
Painting	○	○	○	○	○	○
Smelting	○	○	○	○	○	○
Welding	○	○	○	○	○	○
Stamping	○	○	○	○	○	○
Air compression/vacuuming	○	○	○	○	○	○
Steel cutting	○	○	○	○	○	○
Thermal oxidation	○	○	○	○	○	○
Transportation and logistics (e.g. trucking, distribution, delivery, etc.)	○	○	○	○	○	○
[Insert text from Other]	○	○	○	○	○	○

* 93. How much do you spend for your **kerosene** consumption (in **PESOS**) for each process **PER MONTH**?

	1 - 300,000	300,001 - 600,000	600,001 - 900,000	900,001 - 1,200,000	1,200,001 - 1,500,000	1,500,001 & up
Air/gas mixing (e.g. Selas mixing, etc.)	○	○	○	○	○	○
Baking	○	○	○	○	○	○
Boiler operation (e.g. for steam generation, etc.)	○	○	○	○	○	○
Burning	○	○	○	○	○	○
Curing (e.g. oven curing, powder paint curing, etc.)	○	○	○	○	○	○
Die casting or wire bonding	○	○	○	○	○	○
Drying or annealing (e.g. oven drying, mold drying, core drying, air handling, etc.)	○	○	○	○	○	○
Engine loading or preparation	○	○	○	○	○	○
Fabrication	○	○	○	○	○	○
Forklift operation	○	○	○	○	○	○
Heat treatment	○	○	○	○	○	○
Ice making	○	○	○	○	○	○
Impregnation	○	○	○	○	○	○
Machine injection or molding	○	○	○	○	○	○
Melting or pre-melting	○	○	○	○	○	○
Metal treatment or pre-treatment	○	○	○	○	○	○
Painting	○	○	○	○	○	○
Smelting	○	○	○	○	○	○
Welding	○	○	○	○	○	○
Stamping	○	○	○	○	○	○
Air compression/vacuuming	○	○	○	○	○	○
Steel cutting	○	○	○	○	○	○
Thermal oxidation	○	○	○	○	○	○
Transportation and logistics (e.g. trucking, distribution, delivery, etc.)	○	○	○	○	○	○
[Insert text from Other]	○	○	○	○	○	○

* 94. How do you procure your **kerosene**?

- ◯ Contracted
- ◯ Buying as needed
- ◯ Other (please specify)

[ ]

IV. Fuels Used in Production - LPG

* 95. Do you use **LPG** in your main production processes?

◯ Yes

◯ No

IV. Fuels Used in Production - LPG

* 96. How important is **LPG** in your main production processes?

- ◯ Not important
- ◯ Slightly important
- ◯ Important
- ◯ Fairly important
- ◯ Very important

IV. Fuels Used in Production - LPG

* 97. Which of the following processes involved in your main production use **LPG**? Please check all that apply.

- [ ] Air/gas mixing (e.g. Selas mixing, etc.)
- [ ] Baking
- [ ] Boiler operation (e.g. for steam generation, etc.)
- [ ] Burning
- [ ] Curing (e.g. oven curing, powder paint curing, etc.)
- [ ] Die casting or wire bonding
- [ ] Drying or annealing (e.g. oven drying, mold drying, core drying, air handling, etc.)
- [ ] Engine loading or preparation
- [ ] Fabrication
- [ ] Forklift operation
- [ ] Heat treatment
- [ ] Ice making
- [ ] Impregnation
- [ ] Machine injection or molding
- [ ] Melting or pre-melting
- [ ] Metal treatment or pre-treatment
- [ ] Painting
- [ ] Smelting
- [ ] Steel cutting
- [ ] Thermal oxidation
- [ ] Transportation and logistics (e.g. trucking, distribution, delivery, etc.)
- [ ] Welding
- [ ] Stamping
- [ ] Air compression/vacuuming
- [ ] Other (please specify)

IV. Fuels Used in Production - LPG

* 98. How much **LPG** do you consume (in **KILOGRAMS**) for each process **PER MONTH**?

	1 - 20,000	20,001 - 40,000	40,001 - 60,000	60,001 - 80,000	80,001 - 100,000	100,001 & up
Air/gas mixing (e.g. Selas mixing, etc.)	○	○	○	○	○	○
Baking	○	○	○	○	○	○
Boiler operation (e.g. for steam generation, etc.)	○	○	○	○	○	○
Burning	○	○	○	○	○	○
Curing (e.g. oven curing, powder paint curing, etc.)	○	○	○	○	○	○
Die casting or wire bonding	○	○	○	○	○	○
Drying or annealing (e.g. oven drying, mold drying, core drying, air handling, etc.)	○	○	○	○	○	○
Engine loading or preparation	○	○	○	○	○	○
Fabrication	○	○	○	○	○	○
Forklift operation	○	○	○	○	○	○
Heat treatment	○	○	○	○	○	○
Ice making	○	○	○	○	○	○
Impregnation	○	○	○	○	○	○
Machine injection or molding	○	○	○	○	○	○
Melting or pre-melting	○	○	○	○	○	○
Metal treatment or pre-treatment	○	○	○	○	○	○
Painting	○	○	○	○	○	○
Smelting	○	○	○	○	○	○
Welding	○	○	○	○	○	○
Stamping	○	○	○	○	○	○
Air compression/vacuuming	○	○	○	○	○	○
Steel cutting	○	○	○	○	○	○
Thermal oxidation	○	○	○	○	○	○
Transportation and logistics (e.g. trucking, distribution, delivery, etc.)	○	○	○	○	○	○
[Insert text from Other]	○	○	○	○	○	○

* 99. How much do you spend for your **LPG** consumption (in **PESOS**) for each process **PER MONTH**?

	1 - 1 million	1,000,001 - 2 million	2,000,001 - 3 million	3,000,001 - 4 million	4,000,001 - 5 million	5,000,001 & up
Air/gas mixing (e.g. Selas mixing, etc.)	○	○	○	○	○	○
Baking	○	○	○	○	○	○
Boiler operation (e.g. for steam generation, etc.)	○	○	○	○	○	○
Burning	○	○	○	○	○	○
Curing (e.g. oven curing, powder paint curing, etc.)	○	○	○	○	○	○
Die casting or wire bonding	○	○	○	○	○	○
Drying or annealing (e.g. oven drying, mold drying, core drying, air handling, etc.)	○	○	○	○	○	○
Engine loading or preparation	○	○	○	○	○	○
Fabrication	○	○	○	○	○	○
Forklift operation	○	○	○	○	○	○
Heat treatment	○	○	○	○	○	○
Ice making	○	○	○	○	○	○
Impregnation	○	○	○	○	○	○
Machine injection or molding	○	○	○	○	○	○
Melting or pre-melting	○	○	○	○	○	○
Metal treatment or pre-treatment	○	○	○	○	○	○
Painting	○	○	○	○	○	○
Smelting	○	○	○	○	○	○
Welding	○	○	○	○	○	○
Stamping	○	○	○	○	○	○
Air compression/vacuuming	○	○	○	○	○	○
Steel cutting	○	○	○	○	○	○
Thermal oxidation	○	○	○	○	○	○
Transportation and logistics (e.g. trucking, distribution, delivery, etc.)	○	○	○	○	○	○
[Insert text from Other]	○	○	○	○	○	○

* 100. How do you procure your **LPG**?

- ◯ Contracted
- ◯ Buying as needed
- ◯ Other (please specify)

IV. Fuels Used in Production - Natural Gas

* 101. Do you use **natural gas** in your main production processes?

- ◯ Yes
- ◯ No

IV. Fuels Used in Production - Natural Gas

* 102. How important is **natural gas** in your main production processes?

- ◯ Not important
- ◯ Slightly important
- ◯ Important
- ◯ Fairly important
- ◯ Very important

IV. Fuels Used in Production - Natural Gas

* 103. Which of the following processes involved in your main production use **natural gas**? Please check all that apply.

- [ ] Air/gas mixing (e.g. Selas mixing, etc.)
- [ ] Baking
- [ ] Boiler operation (e.g. for steam generation, etc.)
- [ ] Burning
- [ ] Curing (e.g. oven curing, powder paint curing, etc.)
- [ ] Die casting or wire bonding
- [ ] Drying or annealing (e.g. oven drying, mold drying, core drying, air handling, etc.)
- [ ] Engine loading or preparation
- [ ] Fabrication
- [ ] Forklift operation
- [ ] Heat treatment
- [ ] Ice making
- [ ] Impregnation
- [ ] Machine injection or molding
- [ ] Melting or pre-melting
- [ ] Metal treatment or pre-treatment
- [ ] Painting
- [ ] Smelting
- [ ] Steel cutting
- [ ] Thermal oxidation
- [ ] Transportation and logistics (e.g. trucking, distribution, delivery, etc.)
- [ ] Welding
- [ ] Stamping
- [ ] Air compression/vacuuming
- [ ] Other (please specify)

IV. Fuels Used in Production - Natural Gas

* 104. How much **natural gas** do you consume (in **MILLION STANDARD CUBIC FEET PER DAY or MMSCFD**) for each process **PER DAY**?

	1 - 20	21 - 40	41 - 60	61 - 80	81 - 100	101 & up
Air/gas mixing (e.g. Selas mixing, etc.)	○	○	○	○	○	○
Baking	○	○	○	○	○	○
Boiler operation (e.g. for steam generation, etc.)	○	○	○	○	○	○
Burning	○	○	○	○	○	○
Curing (e.g. oven curing, powder paint curing, etc.)	○	○	○	○	○	○
Die casting or wire bonding	○	○	○	○	○	○
Drying or annealing (e.g. oven drying, mold drying, core drying, air handling, etc.)	○	○	○	○	○	○
Engine loading or preparation	○	○	○	○	○	○
Fabrication	○	○	○	○	○	○
Forklift operation	○	○	○	○	○	○
Heat treatment	○	○	○	○	○	○
Ice making	○	○	○	○	○	○
Impregnation	○	○	○	○	○	○
Machine injection or molding	○	○	○	○	○	○
Melting or pre-melting	○	○	○	○	○	○
Metal treatment or pre-treatment	○	○	○	○	○	○
Painting	○	○	○	○	○	○
Smelting	○	○	○	○	○	○
Welding	○	○	○	○	○	○
Stamping	○	○	○	○	○	○
Air compression/vacuuming	○	○	○	○	○	○
Steel cutting	○	○	○	○	○	○
Thermal oxidation	○	○	○	○	○	○
Transportation and logistics (e.g. trucking, distribution, delivery, etc.)	○	○	○	○	○	○
[Insert text from Other]	○	○	○	○	○	○

* 105. How much do you spend for your **natural gas** consumption (in **PESOS**) for each process **PER MONTH**?

	1 - 1 million	1,000,001 - 2 million	2,000,001 - 3 million	3,000,001 - 4 million	4,000,001 - 5 million	5,000,001 & up
Air/gas mixing (e.g. Selas mixing, etc.)	○	○	○	○	○	○
Baking	○	○	○	○	○	○
Boiler operation (e.g. for steam generation, etc.)	○	○	○	○	○	○
Burning	○	○	○	○	○	○
Curing (e.g. oven curing, powder paint curing, etc.)	○	○	○	○	○	○
Die casting or wire bonding	○	○	○	○	○	○
Drying or annealing (e.g. oven drying, mold drying, core drying, air handling, etc.)	○	○	○	○	○	○
Engine loading or preparation	○	○	○	○	○	○
Fabrication	○	○	○	○	○	○
Forklift operation	○	○	○	○	○	○
Heat treatment	○	○	○	○	○	○
Ice making	○	○	○	○	○	○
Impregnation	○	○	○	○	○	○
Machine injection or molding	○	○	○	○	○	○
Melting or pre-melting	○	○	○	○	○	○
Metal treatment or pre-treatment	○	○	○	○	○	○
Painting	○	○	○	○	○	○
Smelting	○	○	○	○	○	○
Welding	○	○	○	○	○	○
Stamping	○	○	○	○	○	○
Air compression/vacuuming	○	○	○	○	○	○
Steel cutting	○	○	○	○	○	○
Thermal oxidation	○	○	○	○	○	○
Transportation and logistics (e.g. trucking, distribution, delivery, etc.)	○	○	○	○	○	○
[Insert text from Other]	○	○	○	○	○	○

* 106. How do you procure your **natural gas**?

- ◯ Contracted
- ◯ Buying as needed
- ◯ Other (please specify)

IV. Fuels Used in Production - Propane

* 107. Do you use **propane** in your main production processes?

◯ Yes

◯ No

IV. Fuels Used in Production - Propane

* 108. How important is **propane** in your main production processes?

- ◯ Not important
- ◯ Slightly important
- ◯ Important
- ◯ Fairly important
- ◯ Very important

IV. Fuels Used in Production - Propane

* 109. Which of the following processes involved in your main production use **propane**? Please check all that apply.

- ☐ Air/gas mixing (e.g. Selas mixing, etc.)
- ☐ Baking
- ☐ Boiler operation (e.g. for steam generation, etc.)
- ☐ Burning
- ☐ Curing (e.g. oven curing, powder paint curing, etc.)
- ☐ Die casting or wire bonding
- ☐ Drying or annealing (e.g. oven drying, mold drying, core drying, air handling, etc.)
- ☐ Engine loading or preparation
- ☐ Fabrication
- ☐ Forklift operation
- ☐ Heat treatment
- ☐ Ice making
- ☐ Impregnation
- ☐ Machine injection or molding
- ☐ Melting or pre-melting
- ☐ Metal treatment or pre-treatment
- ☐ Painting
- ☐ Smelting
- ☐ Steel cutting
- ☐ Thermal oxidation
- ☐ Transportation and logistics (e.g. trucking, distribution, delivery, etc.)
- ☐ Welding
- ☐ Stamping
- ☐ Air compression/vacuuming
- ☐ Other (please specify)

IV. Fuels Used in Production - Propane

* 110. How much **propane** do you consume (in **KILOGRAMS**) for each process **PER MONTH**?

	1 - 20,000	20,001 - 40,000	40,001 - 60,000	60,001 - 80,000	80,001 - 100,000	100,001 & up
Air/gas mixing (e.g. Selas mixing, etc.)	○	○	○	○	○	○
Baking	○	○	○	○	○	○
Boiler operation (e.g. for steam generation, etc.)	○	○	○	○	○	○
Burning	○	○	○	○	○	○
Curing (e.g. oven curing, powder paint curing, etc.)	○	○	○	○	○	○
Die casting or wire bonding	○	○	○	○	○	○
Drying or annealing (e.g. oven drying, mold drying, core drying, air handling, etc.)	○	○	○	○	○	○
Engine loading or preparation	○	○	○	○	○	○
Fabrication	○	○	○	○	○	○
Forklift operation	○	○	○	○	○	○
Heat treatment	○	○	○	○	○	○
Ice making	○	○	○	○	○	○
Impregnation	○	○	○	○	○	○
Machine injection or molding	○	○	○	○	○	○
Melting or pre-melting	○	○	○	○	○	○
Metal treatment or pre-treatment	○	○	○	○	○	○
Painting	○	○	○	○	○	○
Smelting	○	○	○	○	○	○
Welding	○	○	○	○	○	○
Stamping	○	○	○	○	○	○
Air compression/vacuuming	○	○	○	○	○	○
Steel cutting	○	○	○	○	○	○
Thermal oxidation	○	○	○	○	○	○
Transportation and logistics (e.g. trucking, distribution, delivery, etc.)	○	○	○	○	○	○
[Insert text from Other]	○	○	○	○	○	○

* 111. How much do you spend for your **propane** consumption (in **PESOS**) for each process **PER MONTH**?

	1 - 1 million	1,000,001 - 2 million	2,000,001 - 3 million	3,000,001 - 4 million	4,000,001 - 5 million	5,000,001 & up
Air/gas mixing (e.g. Selas mixing, etc.)						
Baking						
Boiler operation (e.g. for steam generation, etc.)						
Burning						
Curing (e.g. oven curing, powder paint curing, etc.)						
Die casting or wire bonding						
Drying or annealing (e.g. oven drying, mold drying, core drying, air handling, etc.)						
Engine loading or preparation						
Fabrication						
Forklift operation						
Heat treatment						
Ice making						
Impregnation						
Machine injection or molding						
Melting or pre-melting						
Metal treatment or pre-treatment						
Painting						
Smelting						
Welding						
Stamping						
Air compression/vacuuming						
Steel cutting						
Thermal oxidation						
Transportation and logistics (e.g. trucking, distribution, delivery, etc.)						
[Insert text from Other]						

* 112. How do you procure your **propane**?

- ◯ Contracted
- ◯ Buying as needed
- ◯ Other (please specify)

IV. Fuels Used in Production - Other Fuel (1)

For the succeeding questions, you will be asked about other fuels that you use in your production processes but were not mentioned previously.

* 113. Do you use **other fuel (1)** in your main production processes that was not mentioned in the previous questions?

○ Yes

○ No

If yes, which fuel?

[ ]

IV. Fuels Used in Production - Other Fuel (1)

* 114. How important is **other fuel (1)** in your main production processes?

- ◯ Not important
- ◯ Slightly important
- ◯ Important
- ◯ Fairly important
- ◯ Very important

IV. Fuels Used in Production - Other Fuel (1)

* 115. Which of the following processes involved in your main production use **other fuel (1)**? Please check all that apply.

- ☐ Air/gas mixing (e.g. Selas mixing, etc.)
- ☐ Baking
- ☐ Boiler operation (e.g. for steam generation, etc.)
- ☐ Burning
- ☐ Curing (e.g. oven curing, powder paint curing, etc.)
- ☐ Die casting or wire bonding
- ☐ Drying or annealing (e.g. oven drying, mold drying, core drying, air handling, etc.)
- ☐ Engine loading or preparation
- ☐ Fabrication
- ☐ Forklift operation
- ☐ Heat treatment
- ☐ Ice making
- ☐ Impregnation
- ☐ Machine injection or molding
- ☐ Melting or pre-melting
- ☐ Metal treatment or pre-treatment
- ☐ Painting
- ☐ Smelting
- ☐ Steel cutting
- ☐ Thermal oxidation
- ☐ Transportation and logistics (e.g. trucking, distribution, delivery, etc.)
- ☐ Welding
- ☐ Stamping
- ☐ Air compression/vacuuming
- ☐ Other (please specify)

IV. Fuels Used in Production - Other Fuel (1)

* 116. How much **other fuel (1)** do you consume for each process **PER DAY**? Please include quantity and unit.

Air/gas mixing (e.g. Selas mixing, etc.)	
Baking	
Boiler operation (e.g. for steam generation, etc.)	
Burning	
Curing (e.g. oven curing, powder paint curing, etc.)	
Die casting or wire bonding	
Drying or annealing (e.g. oven drying, mold drying, core drying, air handling, etc.)	
Engine loading or preparation	
Fabrication	
Forklift operation	
Heat treatment	
Ice making	
Impregnation	
Machine injection or molding	
Melting or pre-melting	
Metal treatment or pre-treatment	
Painting	
Smelting	
Welding	
Stamping	

Air compression/vacuuming

Steel cutting

Thermal oxidation

Transportation and logistics (e.g. trucking, distribution, delivery, etc.)

[Insert text from Other]

* 117. How much do you spend for your **other fuel (1)** consumption (in **PESOS**) for each process **PER MONTH**? Write only the amount.

Air/gas mixing (e.g. Selas mixing, etc.)

Baking

Boiler operation (e.g. for steam generation, etc.)

Burning

Curing (e.g. oven curing, powder paint curing, etc.)

Die casting or wire bonding

Drying or annealing (e.g. oven drying, mold drying, core drying, air handling, etc.)

Engine loading or preparation

Fabrication

Forklift operation

Heat treatment

Ice making

Impregnation

Machine injection or molding

Melting or pre-melting

Metal treatment or pre-treatment

Painting

Smelting

Welding

Stamping

Air compression/vacuuming

Steel cutting

Thermal oxidation

Transportation and logistics (e.g. trucking, distribution, delivery, etc.)

[Insert text from Other]

* 118. How do you procure your **other fuel (1)**?

- Contracted
- Buying as needed
- Other (please specify)

IV. Fuels Used in Production - Other Fuel (2)

* 119. Do you use **other fuel (2)** in your main production processes that was not mentioned in the previous questions?

- ◯ Yes
- ◯ No

If yes, which fuel?

IV. Fuels Used in Production - Other Fuel (2)

* 120. How important is **other fuel (2)** in your main production processes?

- ◯ Not important
- ◯ Slightly important
- ◯ Important
- ◯ Fairly important
- ◯ Very important

IV. Fuels Used in Production - Other Fuel (2)

* 121. Which of the following processes involved in your main production use **other fuel (2)**? Please check all that apply.

- [ ] Air/gas mixing (e.g. Selas mixing, etc.)
- [ ] Baking
- [ ] Boiler operation (e.g. for steam generation, etc.)
- [ ] Burning
- [ ] Curing (e.g. oven curing, powder paint curing, etc.)
- [ ] Die casting or wire bonding
- [ ] Drying or annealing (e.g. oven drying, mold drying, core drying, air handling, etc.)
- [ ] Engine loading or preparation
- [ ] Fabrication
- [ ] Forklift operation
- [ ] Heat treatment
- [ ] Ice making
- [ ] Impregnation
- [ ] Machine injection or molding
- [ ] Melting or pre-melting
- [ ] Metal treatment or pre-treatment
- [ ] Painting
- [ ] Smelting
- [ ] Steel cutting
- [ ] Thermal oxidation
- [ ] Transportation and logistics (e.g. trucking, distribution, delivery, etc.)
- [ ] Welding
- [ ] Stamping
- [ ] Air compression/vacuuming
- [ ] Other (please specify)

IV. Fuels Used in Production - Other Fuel (2)

* 122. How much **other fuel (2)** do you consume for each process **PER DAY**? Please include quantity and unit.

Air/gas mixing (e.g. Selas mixing, etc.)	
Baking	
Boiler operation (e.g. for steam generation, etc.)	
Burning	
Curing (e.g. oven curing, powder paint curing, etc.)	
Die casting or wire bonding	
Drying or annealing (e.g. oven drying, mold drying, core drying, air handling, etc.)	
Engine loading or preparation	
Fabrication	
Forklift operation	
Heat treatment	
Ice making	
Impregnation	
Machine injection or molding	
Melting or pre-melting	
Metal treatment or pre-treatment	
Painting	
Smelting	
Welding	
Stamping	

Air compression/vacuuming

Steel cutting

Thermal oxidation

Transportation and logistics (e.g. trucking, distribution, delivery, etc.)

[Insert text from Other]

* 123. How much do you spend for your **other fuel (2)** consumption (in **PESOS**) for each process **PER MONTH**? Write only the amount.

Air/gas mixing (e.g. Selas mixing, etc.)

Baking

Boiler operation (e.g. for steam generation, etc.)

Burning

Curing (e.g. oven curing, powder paint curing, etc.)

Die casting or wire bonding

Drying or annealing (e.g. oven drying, mold drying, core drying, air handling, etc.)

Engine loading or preparation

Fabrication

Forklift operation

Heat treatment

Ice making

Impregnation

Machine injection or molding

Melting or pre-melting

Metal treatment or pre-treatment

Painting

Smelting

Welding

Stamping

Air compression/vacuuming

Steel cutting

Thermal oxidation

Transportation and logistics (e.g. trucking, distribution, delivery, etc.)

[Insert text from Other]

* 124. How do you procure your **other fuel (2)**?

- ○ Contracted
- ○ Buying as needed
- ○ Other (please specify)

IV. Fuels Used in Production - Other Fuel (3)

* 125. Do you use **other fuel (3)** in your main production processes that was not mentioned in the previous questions?

- ◯ Yes
- ◯ No

If yes, which fuel?

IV. Fuels Used in Production - Other Fuel (3)

* 126. How important is **other fuel (3)** in your main production processes?

- ◯ Not important
- ◯ Slightly important
- ◯ Important
- ◯ Fairly important
- ◯ Very important

IV. Fuels Used in Production - Other Fuel (3)

* 127. Which of the following processes involved in your main production use **other fuel (3)**? Please check all that apply.

- [ ] Air/gas mixing (e.g. Selas mixing, etc.)
- [ ] Baking
- [ ] Boiler operation (e.g. for steam generation, etc.)
- [ ] Burning
- [ ] Curing (e.g. oven curing, powder paint curing, etc.)
- [ ] Die casting or wire bonding
- [ ] Drying or annealing (e.g. oven drying, mold drying, core drying, air handling, etc.)
- [ ] Engine loading or preparation
- [ ] Fabrication
- [ ] Forklift operation
- [ ] Heat treatment
- [ ] Ice making
- [ ] Impregnation
- [ ] Machine injection or molding
- [ ] Melting or pre-melting
- [ ] Metal treatment or pre-treatment
- [ ] Painting
- [ ] Smelting
- [ ] Steel cutting
- [ ] Thermal oxidation
- [ ] Transportation and logistics (e.g. trucking, distribution, delivery, etc.)
- [ ] Welding
- [ ] Stamping
- [ ] Air compression/vacuuming
- [ ] Other (please specify)

IV. Fuels Used in Production - Other Fuel (3)

* 128. How much **other fuel (3)** do you consume for each process **PER DAY**? Please include quantity and unit.

Air/gas mixing (e.g. Selas mixing, etc.)

Baking

Boiler operation (e.g. for steam generation, etc.)

Burning

Curing (e.g. oven curing, powder paint curing, etc.)

Die casting or wire bonding

Drying or annealing (e.g. oven drying, mold drying, core drying, air handling, etc.)

Engine loading or preparation

Fabrication

Forklift operation

Heat treatment

Ice making

Impregnation

Machine injection or molding

Melting or pre-melting

Metal treatment or pre-treatment

Painting

Smelting

Welding

Stamping

Air compression/vacuuming

Steel cutting

Thermal oxidation

Transportation and logistics (e.g. trucking, distribution, delivery, etc.)

[Insert text from Other]

* 129. How much do you spend for your **other fuel (3)** consumption (in **PESOS**) for each process **PER MONTH**? Write only the amount.

Air/gas mixing (e.g. Selas mixing, etc.)

Baking

Boiler operation (e.g. for steam generation, etc.)

Burning

Curing (e.g. oven curing, powder paint curing, etc.)

Die casting or wire bonding

Drying or annealing (e.g. oven drying, mold drying, core drying, air handling, etc.)

Engine loading or preparation

Fabrication

Forklift operation

Heat treatment

Ice making

Impregnation

Machine injection or molding

Melting or pre-melting

Metal treatment or pre-treatment

Painting

Smelting

Welding	
Stamping	
Air compression/vacuuming	
Steel cutting	
Thermal oxidation	
Transportation and logistics (e.g. trucking, distribution, delivery, etc.)	
[Insert text from Other]	

* 130. How do you procure your **other fuel (3)**?

- ○ Contracted
- ○ Buying as needed
- ○ Other (please specify)

V. Aptitude on Alternative Fuels and Primary Energies

In this section, you will be asked about your knowledge, considerations, and opinions on alternative fuels and primary energies as well as your experiences in using them.

V. Aptitude on Alternative Fuels and Primary Energies - Natural Gas

Natural gas is different from LPG that is typically used for cooking.

* 131. With 1 being limited, and 5 being advanced, what is the extent of your knowledge on **natural gas** as fuel?

1	2	3	4	5
◯	◯	◯	◯	◯

* 132. Do you think **natural gas** is safe to utilize as fuel in your production process?

- ◯ Yes
- ◯ No

* 133. Do you think **natural gas** is cost-competitive relative to the fuels and primary energies you are currently using?

- ◯ Yes
- ◯ No

* 134. How much (in **PESOS**) do you think can you save if you use **natural gas**?

- ◯ 1 to 200,000
- ◯ 200,001 to 400,000
- ◯ 400,001 to 600,000
- ◯ 600,001 to 800,000
- ◯ 800,001 to 1,000,000
- ◯ 1,000,001 and above

* 135. In case **natural gas** would be made available to you, what would be your considerations in using it in your production processes? With 1 being the most important, rank the following.

You may choose a number from the dropdown, or drag and drop to reorder.

- Price
- Supply stability and reliability
- Safety and security
- Environmental concerns
- Compatibility of machines and equipment
- Retrofitting costs of equipment

136. How much (in **PESOS**) do you think will the retrofitting of equipment cost?

- 1 to 200,000
- 200,001 to 400,000
- 400,001 to 600,000
- 600,001 to 800,000
- 800,001 to 1,000,000
- 1,000,001 and above

* 137. Are you open to switching to **natural gas** in your production processes, self-generation, and back-up generation of power?

- Yes
- No

V. Aptitude on Alternative Fuels and Primary Energies - Natural Gas

* 138. In case you decide to switch to **natural gas** in your **PRODUCTION PROCESSES**, which among the following fuels would you most likely replace with it? Please check all that apply.

- [ ] Biodiesel
- [ ] Bunker
- [ ] Coal
- [ ] Diesel
- [ ] Gasoline
- [ ] Kerosene
- [ ] LPG
- [ ] Propane
- [ ] Other (please specify)

* 139. In case you decide to switch to **natural gas** in **SELF-GENERATION OF POWER**, which among the following fuels and primary energies would you most likely replace with it? Please check all that apply.

- [ ] Biodiesel
- [ ] Bunker
- [ ] Coal
- [ ] Diesel
- [ ] Gasoline
- [ ] Kerosene
- [ ] LPG
- [ ] Propane
- [ ] Solar
- [ ] Wind
- [ ] Other (please specify)

* 140. In case you decide to switch to **natural gas** in **BACK-UP POWER GENERATION**, which among the following fuels and primary energies would you most likely replace with it? Please check all that apply.

- ☐ Biodiesel
- ☐ Bunker
- ☐ Coal
- ☐ Diesel
- ☐ Gasoline
- ☐ Kerosene
- ☐ LPG
- ☐ Propane
- ☐ Solar
- ☐ Wind
- ☐ Other (please specify)

* 141. In case you decide to switch to **natural gas**, what would be your requirement in **MILLION STANDARD CUBIC FEET PER DAY** (**MMSCFD**)?

- ○ 1 to 20
- ○ 21 to 40
- ○ 41 to 60
- ○ 61 to 80
- ○ 81 to 100
- ○ 101 and above

V. Aptitude on Alternative Fuels and Primary Energies - Natural Gas

* 142. Do you have a subsidiary / parent / partner company in other countries?

◯ Yes

◯ No

V. Aptitude on Alternative Fuels and Primary Energies - Natural Gas

* 143. Does your subsidiary / parent / partner company have any experiences using **natural gas** in their production processes?

- ◯ Yes
- ◯ No

V. Aptitude on Alternative Fuels and Primary Energies - Solar

In the next questions, solar is defined as energy used to generate electricity through photovoltaic solar panels.

* 144. With 1 being limited, and 5 being advanced, what is the extent of your knowledge on **solar** as primary energy?

1	2	3	4	5
○	○	○	○	○

* 145. Do you think **solar** is safe to utilize as primary energy in your production process?

- ○ Yes
- ○ No

* 146. Do you think **solar** is cost-competitive relative to the fuels and primary energies you are currently using?

- ○ Yes
- ○ No

* 147. How much (in **PESOS**) do you think can you save if you use **solar**?

- ○ 1 to 200,000
- ○ 200,001 to 400,000
- ○ 400,001 to 600,000
- ○ 600,001 to 800,000
- ○ 800,001 to 1,000,000
- ○ 1,000,001

* 148. In case **solar** would be made available to you, what would be your considerations in using it in your production processes? With 1 being the most important, rank the following.

You may choose a number from the dropdown, or drag and drop to reorder.

- Price
- Supply stability and reliability
- Safety and security
- Environmental concerns
- Compatibility of machines and equipment
- Retrofitting costs of equipment

149. How much (in **PESOS**) do you think will the retrofitting of equipment cost?

- 1 to 200,000
- 200,001 to 400,000
- 400,001 to 600,000
- 600,001 to 800,000
- 800,001 to 1,000,000
- 1,000,001 and above

* 150. Are you open to switching to **solar** in you production processes, self-generation and back-up generation of power?

- Yes
- No

V. Aptitude on Alternative Fuels and Primary Energies - Solar

* 151. In case you decide to switch to **solar** in your **PRODUCTION PROCESSES**, which among the following fuels would you most likely replace with it? Please check all that apply.

- [ ] Biodiesel
- [ ] Bunker
- [ ] Coal
- [ ] Diesel
- [ ] Gasoline
- [ ] Kerosene
- [ ] LPG
- [ ] Propane
- [ ] Other (please specify)

* 152. In case you decide to switch to **solar** in **SELF-GENERATION OF POWER**, which among the following fuels and primary energies would you most likely replace with it? Please check all that apply.

- [ ] Biodiesel
- [ ] Bunker
- [ ] Coal
- [ ] Diesel
- [ ] Gasoline
- [ ] Kerosene
- [ ] LPG
- [ ] Natural gas
- [ ] Propane
- [ ] Wind
- [ ] Other (please specify)

* 153. In case you decide to switch to **solar** in **BACK-UP POWER GENERATION**, which among the following fuels and primary energies would you most likely replace with it? Please check all that apply.

- [ ] Biodiesel
- [ ] Bunker
- [ ] Coal
- [ ] Diesel
- [ ] Gasoline
- [ ] Kerosene
- [ ] LPG
- [ ] Natural gas
- [ ] Propane
- [ ] Wind
- [ ] Other (please specify)

* 154. In case you decide to switch to **solar**, what would be your requirement in **KILOWATT-HOURS**?

- ○ 1 to 10,000
- ○ 10,001 to 20,000
- ○ 20,001 to 30,000
- ○ 30,001 to 40,000
- ○ 40,001 to 50,000
- ○ 50,001 and above

V. Aptitude on Alternative Fuels and Primary Energies - Solar

* 155. Do you have a subsidiary / parent / partner company in other countries?

- ◯ Yes
- ◯ No

V. Aptitude on Alternative Fuels and Primary Energies - Solar

* 156. Does your subsidiary / parent / partner company have any experiences using **solar** in their production processes?

- Yes
- No

V. Aptitude on Alternative Fuels and Primary Energies - Wind

In the next questions, wind is defined as energy used to generate electricity through wind turbines or wind energy converters.

* 157. With 1 being limited, and 5 being advanced, what is the extent of your knowledge on **wind** as primary energy?

1	2	3	4	5
◯	◯	◯	◯	◯

* 158. Do you think **wind** is safe to utilize as primary energy in your production process?

◯ Yes

◯ No

* 159. Do you think **wind** is cost-competitive relative to the fuels and primary energies you are currently using?

◯ Yes

◯ No

* 160. How much (in **PESOS**) do you think can you save if you use **wind**?

◯ 1 to 200,000

◯ 200,001 to 400,000

◯ 400,001 to 600,000

◯ 600,001 to 800,000

◯ 800,001 to 1,000,000

◯ 1,000,001 and above

* 161. In case **wind** would be made available to you, what would be your considerations in using it in your production processes? With 1 being the most important, rank the following.

You may choose a number from the dropdown, or drag and drop to reorder.

- Price
- Supply stability and reliability
- Safety and security
- Environmental concerns
- Compatibility of machines and equipment
- Retrofitting costs of equipment

162. How much (in **PESOS**) do you think will the retrofitting of equipment cost?

- 1 to 200,000
- 200,001 to 400,000
- 400,001 to 600,000
- 600,001 to 800,000
- 800,001 to 1,000,000
- 1,000,001 and above

* 163. Are you open to switching to **wind** in you production processes, self-generation and back-up generation of power?

- Yes
- No

V. Aptitude on Alternative Fuels and Primary Energies - Wind

* 164. In case you decide to switch to **wind** in your **PRODUCTION PROCESSES**, which among the following fuels would you most likely replace with it? Please check all that apply.

- ☐ Biodiesel
- ☐ Bunker
- ☐ Coal
- ☐ Diesel
- ☐ Gasoline
- ☐ Kerosene
- ☐ LPG
- ☐ Propane
- ☐ Other (please specify)

* 165. In case you decide to switch to **wind** in **SELF-GENERATION OF POWER**, which among the following fuels and primary energies would you most likely replace with it? Please check all that apply.

- ☐ Biodiesel
- ☐ Bunker
- ☐ Coal
- ☐ Diesel
- ☐ Gasoline
- ☐ Kerosene
- ☐ LPG
- ☐ Natural gas
- ☐ Propane
- ☐ Solar
- ☐ Other (please specify)

* 166. In case you decide to switch to **wind** in **BACK-UP POWER GENERATION**, which among the following fuels and primary energies would you most likely replace with it? Please check all that apply.

- [ ] Biodiesel
- [ ] Bunker
- [ ] Coal
- [ ] Diesel
- [ ] Gasoline
- [ ] Kerosene
- [ ] LPG
- [ ] Natural gas
- [ ] Propane
- [ ] Solar
- [ ] Other (please specify)

* 167. In case you decide to switch to **wind**, what would be your requirement in **KILOWATT-HOURS**?

- ( ) 1 to 10,000
- ( ) 10,001 to 20,000
- ( ) 20,001 to 30,000
- ( ) 30,001 to 40,000
- ( ) 40,001 to 50,000
- ( ) 50,001 and above

V. Aptitude on Alternative Fuels and Primary Energies - Wind

* 168. Do you have a subsidiary / parent / partner company in other countries?

- ◯ Yes
- ◯ No

V. Aptitude on Alternative Fuels and Primary Energies - Wind

* 169. Does your subsidiary / parent / partner company have any experiences using **wind** in their production processes?

◯ Yes

◯ No

VI. Other Questions

* 170. When considering a business expansion in the Philippines, what are your considerations? With 1 being the most important, rank the following.

You may choose a number from the dropdown, or drag and drop to reorder.

- Cost of manpower
- Cost of raw materials
- Cost of electricity
- Cost of fuels

VII. Respondent Information

In this section, you will be asked to provide your contact information, so we may contact you in the future for any clarifications. You will also be asked to provide your position in the company and of other respondents for profiling.

VII. Respondent Information

* 171. In case we have to clarify or validate some responses, may we contact you via telephone or email in the future?

- ◯ Yes
- ◯ No

VII. Respondent Information

* 172. Kindly provide the information below, so we can contact you in the future. Provide only your work/office telephone number and email address.

Name of primary respondent

Position of primary respondent

Work telephone number

Work email address

173. Were there any other person/s who contributed in answering this survey? If yes, kindly provide their positions in the company. Enumerate the positions of at most three persons.

Position of other respondent (1)

Position of other respondent (2)

Position of other respondent (3)

Survey for Philippine Special Economic Zones

About the survey

This research is being undertaken by the UP Statistical Center Research Foundation, Inc. - Gas Policy Development Project (UPSCRFI-GPDP), the Philippine Economic Zone Authority (PEZA), and the Department of Energy (DOE) to establish baseline information and gather market profile of Philippine Special Economic Zones.

About the GPDP

The Gas Policy Development Project (GPDP) is a project that aims to provide technical assistance to the Department of Energy in implementing the Philippine Downstream Natural Gas Regulation (PDNGR) Circular DC2017-11-0012.

GPDP is implemented by the U.P. Statistical Center Research Foundation, Inc. (UPSCRFI) and supported by the U.S. Department of State through a cooperative agreement under the U.S. Asia Enhancing Development and Growth Through Energy (EDGE) initiative.

For further inquiries, you may contact the project via **981-8500 local 3509** or **infogpdp.ph@gmail.com**.
