# Supplementary material for: Survey data on energy and fuel use of firms in economic zones in the Philippines
Source: Data Brief. 2021 Nov 26;39:107637. doi: 10.1016/j.dib.2021.107637 (PMC8645435; doi:10.1016/j.dib.2021.107637)
Supplement: Supplementary file 1 — Appendix 1 Anonymized raw data (DIB Energy Ravago et al 2021_Data.csv) Appendix 2 Data dictionary (DIB Energy Ravago et al 2021_Dictionary.txt) Appendix 3 FGD Survey questionnaire (DIB Energy Ravago et al 2021_FGD questionnaire.pdf) Appendix 4 Survey questionnaire (DIB Energy Ravago et al 2021_Survey questionaire.pdf) Appendix 5 General results from the survey (DIB Energy Ravago et al 2021_General results.pdf) [file Supplementary Appendix 5 DIB Energy Ravago et al 2021.pdf › Supplementary Appendix 5 DIB Energy Ravago et al 2021.pdf]

Survey Data on Energy and Fuel Use of Firms in Economic Zones in the Philippines
Supplementary Appendix 5: General Results from the Survey

Authors

Majah-Leah Ravago[1], Raul Fabella[2], Karl Robert Jandoc[2], Renzi Frias[3,4], J. Kathleen Magadia[4]

Affiliations

1. Department of Economics, Ateneo de Manila University, Room 409 Leong Hall, Katipunan Avenue, Loyola Heights, 1108, Quezon City, Philippines
2. School of Economics, University of the Philippines, Guerrero corner Osmeña Streets, Diliman, 1101, Quezon City, Philippines
3. School of Statistics, University of the Philippines, T. M. Kalaw Street, Diliman, 1101, Quezon City, Philippines
4. Gas Policy Development Project, UP Statistical Center Research Foundation, Inc., School of Statistics, University of the Philippines, Quirino Avenue Kalaw Street, Diliman, 1101, Quezon City, Philippines

Corresponding author

Majah-Leah Ravago (mravago@ateneo.edu)

This Supplementary Appendix presents the general results of the survey on energy and fuel usage of locators in manufacturing and agro-industrial economic zones in the Philippines.

This document is organized the same way as the survey questionnaire (see Supplementary Appendix 4). In addition, section labels, section numbers, questions, and question numbers used in this document correspond to the variable names and labels used in Supplementary Appendices 1 and 2 or the survey data set and data dictionary, respectively.

Section VII (Respondent Information) of the survey questionnaire is not included in this document. Section VII contains identifiable personal data of the respondents. Responses recorded in Section VII were permanently deleted in the database for data privacy protection and in compliance with institutional ethics.

List of Tables

Table I-1. Percentage and number of firms, by ecozone and province 5
Table I-2. Summary statistics on personnel count and monthly salary expense 6
Table I-3. Percentage of personnel and monthly salary expense, by category 6
Table I-4. Summary statistics on book value 6
Table II-1. Number of firms by product destination 7
Table II-2. Summary statistics on annual production sales, 2018 7
Table II-3. Percentage and number of firms by peak and low months 7
Table II-4. Summary statistics on operating days of main production facilities, peak and low months 8
Table II-5. Summary statistics on operating hours of main production facilities, peak and low months 8
Table II-6. Summary statistics on operating days of auxiliary facilities, peak and low months 8
Table II-7. Summary statistics on operating hours of auxiliary facilities, peak and low months 9
Table II-8. Percentage and number of firms with distribution and delivery expense, peak and low months 9
Table II-9. Percentage and number of firms by distribution and delivery expense level, peak and low months 9
Table III-1. Percentage and number of firms by electricity source 10
Table III-2. Percentage share of electricity source in supply requirement 10
Table III-3. Percentage and number of firms that source from RES, by previous source 10
Table III-4. Percentage and number of firms that switched RES 11
Table III-5. Percentage and number of firms that switched RES, by frequency of switch 11
Table III-6. Percentage and number of firms with self-generation, by fuel 11
Table III-7. Number of firms with back-up generation, by fuel 12
Table III-8. Summary statistics on monthly electricity consumption and expenditure 12
Table III-9. Number of firms per electricity rate interval and source 13
Table III-10. Summary statistics on water consumption and expenditure 13
Table III-11. Percentage and number of firms per water rate interval 14
Table III-12. Number of firms by consideration in switching electricity provider, by degree of importance 14

Ravago, Majah-Leah; Fabella, Raul; Jandoc, Karl Robert; Frias, Renzi; Magadia, J. Kathleen (2021), "Survey Data on Energy and Fuel Use of Firms in Economic Zones in the Philippines", Data in Brief.

Table III-13. Number of firms implementing energy conservation measures, by measure 16
Table III-14. Number of firms by reason for not implementing energy conservation measures, by degree of importance 16
Table IV-1. Mean share of fuel in production fuel mix 19
Table IV-2. Percentage and number of firms using fuel, by fuel 19
Table IV-3. Percentage and number of firms using fuel, by degree of importance and fuel 20
Table IV-4. Number of firms involving each production process, by fuel 21
Table IV-5. Percentage and number of firms by mode of procurement and fuel 22
Table IV-6. Number of firms using diesel, by production process, consumption level, and expenditure level 23
Table IV-7. Number of firms using gasoline, by production process, consumption level, and expenditure level 24
Table IV-8. Number of firms using kerosene, by production process, consumption level, and expenditure level 25
Table IV-9. Number of firms using LPG, by production process, consumption level, and expenditure level 26
Table IV-10. Number of firms using natural gas, by production process, consumption level, and expenditure level 27
Table IV-11. Number of firms using propane, by production process, consumption level, and expenditure level 27
Table V-1. Summary statistics and percentage of firms on extent of knowledge, by fuel/source 33
Table V-2. Percentage and number of firms on safety of alternative fuels and primary energies, by fuel/source 33
Table V-3. Percentage and number of firms on cost-competitiveness of alternative fuels and primary energies, by fuel/source 34
Table V-4. Percentage and number of firms on savings in alternative fuels and primary energies, by saving interval and fuel/source 34
Table V-5. Number of firms by consideration in using alternative fuels and primary energies in production, by degree of importance and fuel/source 35
Table V-6. Percentage and number of firms on retrofitting cost of equipment, by fuel/source 36
Table V-7. Percentage and number of firms open to switch to alternative fuels and primary energies, by fuel/source 36
Table V-8. Number of firms willing to replace current fuels with alternative fuels and primary energies, by fuel, alternative fuel/source, and purpose 37
Table V-9. Percentage and number of firms willing to switch, by energy requirement and fuel/source 37
Table V-10. Percentage and number of firms with subsidiary/parent/partner company with experience in using alternative fuels and primary energies, by fuel/source 38
Table VI-1. Number of firms by consideration in business expansion, by degree of importance 39

List of Figures

Figure III-1. Percentage and number of firms participating in PEZA energy-related initiatives 14
Figure III-2. Percentage and number of firms aware of PEZA energy-related programs, by program 15
Figure III-3. Percentage and number of firms implementing energy conservation measures 15

Ravago, Majah-Leah; Fabella, Raul; Jandoc, Karl Robert; Frias, Renzi; Magadia, J. Kathleen (2021), “Survey Data on Energy and Fuel Use of Firms in Economic Zones in the Philippines”, Data in Brief.

SECTION I – General Information

This section covers basic ecozone and firm information; personnel; and book value. This section covers Questions 1 to 9 of the survey questionnaire.

However, Question 1 is not included in this section as it contains identifiable ecozone information. In addition, Question 2 only shows the province where the ecozones are located. All other identifiable information of the firms and ecozones were permanently deleted in the database for data privacy protection and in compliance with institutional ethics. Only the basic geographic data (e.g. city or municipality, province, ZIP code) were stored.

Definitions used in this section: *Tangible fixed assets* refer to physical assets required and for use of the company and is expected to have a productive life of more than one year. They include land, buildings, other structure and land improvements, transport equipment such as cars, trucks, aircrafts, and ships, machinery and equipment, valuables such as paintings and sculptures, and other tangible fixed assets such as fixtures and furniture. On the other hand, *book value* refers to the initial or acquisition cost of tangible fixed assets less accumulated depreciation charges.

Table I-1. Percentage and number of firms, by ecozone and province

Q1. What is the name of your ecozone?	%	N
**Batangas**	**10.43**	**12**
Lima Technology Center	6.09	7
First Philippine Industry Park	3.48	4
Keppel Philippines Marine SEZ	0.87	1
**Benguet**	**0.87**	**1**
Baguio City Economic Zone	0.87	1
**Bulacan**	**0.87**	**1**
Victoria Wave Special Economic Zone	0.87	1
**Cavite**	**8.7**	**10**
Golden Mile Business Park	6.09	7
People's Technology Complex	1.74	2
Golden Gate Business Park-Cavite Export Processing Zone	0.87	1
**Cebu**	**7.83**	**9**
Mactan Economic Zone	3.48	4
West Cebu Industrial Park	2.61	3
Cebu Light Industrial Park	1.74	2
**Laguna**	**64.35**	**74**
Laguna Technopark SEZ	19.13	22
Carmelray Industrial Park II	13.91	16
Calamba Premiere International Park	13.04	15
Filinvest Technology Park Calamba	4.35	5
Laguna International Industrial Park	4.35	5
Light Industry & Science Park I	3.48	4
Greenfield Automotive Park	2.61	3

Laguna Technopark Annex	1.74	2
Light Industry & Science Park II	0.87	1
Toyota Sta. Rosa (Laguna) SEZ	0.87	1
**Metro Manila**	**0.87**	**1**
MacroAsia Ecozone	0.87	1
**Pampanga**	**6.09**	**7**
Pampanga Economic Zone	4.35	5
TECO Industrial Park	1.74	2
Total	100.00	115

Table I-2. Summary statistics on personnel count and monthly salary expense

Statistic	Q3. How many personnel does your company have?	Q5. How much is your monthly total expense (in pesos) for the salary of all personnel?
N	115	115
Mean	494	7,427,455
Standard deviation	964	21,700,000
Minimum	4	74,898
Maximum	6000	159,000,000

Table I-3. Percentage of personnel and monthly salary expense, by category

Category	Q4. How much of the total number of personnel is each category? (%)	Q6. How much (in %) of the monthly total expense for the salary of all personnel is allotted per each category? (%)
Administrative and support	27.41	34.82
Production and technical	72.59	65.18
Total	100.00	100.00

Table I-4. Summary statistics on book value

Statistic	Q7-9. What is the estimated book value (in pesos) of your tangible fixed assets as of December 2018?
N	115
Mean	2,520,000,000
Standard deviation	7,820,000,000
Minimum	100,000,000
Maximum	50,000,000,000

Note: Question 7 is a filter question. Depending on the chosen response option in Question 7, the respondent is redirected to either Question 8 or Question 9 where he/she is asked to provide a more specific response.

SECTION II – Production Schedule and Operation

This section covers information on production sales; and peak and low month schedules and operations. This section covers Questions 10 to 27 of the survey questionnaire.

Table II-1. Number of firms by product destination

Q10. Are your products sold domestically or exported?	%	N
Sold domestically	14.78	17
Exported	43.48	50
Both	41.74	48
Total	100.00	115

Table II-2. Summary statistics on annual production sales, 2018

Statistic	Q11-13. How much was your annual production sales (in pesos) in 2018?
N	115
Mean	3,990,000,000
Standard deviation	9,070,000,000
Minimum	100,000,000
Maximum	50,000,000,000

Note: Question 11 is a filter question. Depending on the chosen response option in Question 11, the respondent is redirected to either Question 12 or Question 13 where he/she is asked to provide a more specific response.

Table II-3. Percentage and number of firms by peak and low months

Q14 / 21. When was you peak month (i.e. month when production was highest) / low month (i.e. month when production was lowest) in 2018?

	Peak month		Low month	
Month	%	N	%	N
January	4.35	5	21.74	25
February	0.00	0	10.43	12
March	9.57	11	6.96	8
April	6.09	7	4.35	5
May	8.70	10	4.35	5
June	11.30	13	3.48	4
July	10.43	12	2.61	3
August	6.09	7	2.61	3
September	7.83	9	2.61	3
October	20.87	24	3.48	4
November	7.83	9	5.22	6
December	6.96	8	31.00	37
Total	100.00	115	100.00	115

Table II-4. Summary statistics on operating days of main production facilities, peak and low months

Q15 / 22. During your peak / low month, how many days did your main production equipment and facilities operate?		
Statistic	Peak month	Low month
N	115	115
Mean	24	20
Standard deviation	8	7
Minimum	0	0
Maximum	31	31

Table II-5. Summary statistics on operating hours of main production facilities, peak and low months

Q16 / 23. During your peak / low month, how many hours per day did your main production facilities and equipment operate?		
Statistic	Peak month	Low month
N	115	115
Mean	17	14
Standard deviation	7	7
Minimum	0	0
Maximum	24	24

Table II-6. Summary statistics on operating days of auxiliary facilities, peak and low months

Q17 / 24. During your peak / low month, how many days did your auxiliary equipment and facilities (e.g. temperature/climate controllers, air conditioners, refrigerators, etc.) operate?		
Statistic	Peak month	Low month
N	115	115
Mean	24	20
Standard deviation	8	7
Minimum	0	0
Maximum	31	31

Table II-7. Summary statistics on operating hours of auxiliary facilities, peak and low months

Q18 / 25. During your peak / low month, how many hours per day did your auxiliary equipment and facilities (e.g. temperature/climate controllers, air conditioners, refrigerators, etc.) operate?

Statistic	Peak month	Low month
N	115	115
Mean	16	14
Standard deviation	7	7
Minimum	0	0
Maximum	24	24

Table II-8. Percentage and number of firms with distribution and delivery expense, peak and low months

Q19 / 26. During your peak / low month, did you spend for the distribution and delivery of your products?

	Peak month		Low month	
	%	N	%	N
Yes	91.00	91	85.00	85
No	9.00	9	15.00	15
Total	100.00	100	100.00	100

Note: These questions were only asked in the primary survey, hence the total number of responses is less than 115.

Table II-9. Percentage and number of firms by distribution and delivery expense level, peak and low months

Q20 / 27. During your peak / low month, how much (in pesos) did you spend for the distribution and delivery of your products?

	Peak month		Low month	
Expense level	%	N	%	N
50 million and below	87.91	80	95.29	81
50,000,001 to 100 million	8.79	8	2.35	2
100,000,001 to 150 million	1.10	1	1.18	1
150,000,001 to 200 million	0.00	0	0.00	0
200,000,001 to 250 million	0.00	0	0.00	0
250,000,001 and above	2.20	2	1.18	1
Total	100.00	91	100.00	85

SECTION III – Utilities

This section covers information on electricity sources, requirements, uses, and considerations; electricity and water consumption and expenditure; and energy conservation. This section covers Questions 28 to 56 of the survey questionnaire with an exception for Question 36. Results of Question 36 are presented in Section IV.

Table III-1. Percentage and number of firms by electricity source

Q28. What are your main sources of electricity?		
Source	%	N
Power plant inside ecozone	4.35	5
Meralco or electric cooperative	70.43	81
Retail electricity supplier (RES)	18.26	21
Direct from generation company (directly connected to NGCP)	5.22	6
Self-generation	1.74	2
Other (private electric companies, PEZA, etc.)	4.35	5
Total	100.00	115

Note: PEZA is Philippine Economic Zone Authority.

Table III-2. Percentage share of electricity source in supply requirement

Q29. How much of your electricity requirement (in percent) is supplied by each source?	
Source	%
Power plant inside ecozone	4.17
Meralco or electric cooperative	67.50
Retail electricity supplier (RES)	17.50
Direct from generation company (directly connected to NGCP)	5.00
Self-generation	1.67
Other (private electric companies, PEZA, etc.)	4.17
Total	100.00

Table III-3. Percentage and number of firms that source from RES, by previous source

Q30. If you chose 'retail electricity supplier (RES)' in the question on electricity sources, where did you get your electricity supply before switching to RES?	%	N
Meralco or electric cooperative	73.33	11
None	26.67	4
Total	100.00	15

Note: This question was only asked in the primary survey, hence total number of responses is less than total number of locators that source electricity from RES.

Table III-4. Percentage and number of firms that switched RES

Q31. After shifting to RES, have you switched retailers?	%	N
Yes	20.00	3
No	80.00	12
Total	100.00	15

Note: This question was only asked in the primary survey, hence total number of responses is less than total number of locators that source electricity from RES.

Table III-5. Percentage and number of firms that switched RES, by frequency of switch

Q32. How many times have you switched?	%	N
1	66.67	2
2	33.33	1
Total	100.00	3

Table III-6. Percentage and number of firms with self-generation, by fuel

Q33. If you chose 'self-generation' in the question on electricity sources, which of the following fuels or primary energies do you use?

Fuel	%	N
Biodiesel	0.00	0
Bunker	0.00	0
Coal	0.00	0
Diesel	50.00	1
Gasoline	0.00	0
Kerosene	0.00	0
LPG	0.00	0
Natural gas	0.00	0
Others*	0.00	0
Propane	0.00	0
Solar	50.00	1
Wind	0.00	0
Total	100.00	2

Note: *Fuel provided by lessor or private company; electricity

Table III-7. Number of firms with back-up generation, by fuel

Q34 / 35. Do you have back-up generation unit/s? If yes, which of the following fuels or primary energies do you use for back-up power generation?	
Fuel	N
Biodiesel	1
Bunker	0
Coal	0
Diesel	37
Gasoline	5
Kerosene	0
LPG	0
Natural gas	0
Others*	3
Propane	0
Solar	0
Wind	0
Total	45

Note: Fuel provide by lessor or private company; electricity

Table III-8. Summary statistics on monthly electricity consumption and expenditure

	Q37-39. For the past 3 months, what was your average monthly electricity consumption in kilowatt-hours?	Q40-42. For the past 3 months, how much did you spend for monthly electricity in pesos?
N	115	115
Mean	209,739.6	3,917,392
Standard deviation	319,183.3	7,029,677
Minimum	10,000.5	500,000.5
Maximum	1,100,001	45,000,000

Note: Question 37 is a filter question. Depending on the chosen response option in Question 37, the respondent is redirected to either Question 38 or Question 39 where he/she is asked to provide a more specific response. The same logic applies to Questions 40 to 42.

Table III-9. Number of firms per electricity rate interval and source

Q43. How much (in PESOS) do you pay per kilowatt-hour of electricity?									
Source	Less than 2.00	2.01 to 4.00	4.01 to 6.00	6.01 to 8.00	8.01 to 10.00	10.01 to 12.00	12.01 to 14.00	More than 14.00	Total
Power plant inside ecozone	—	—	3	2	—	—	—	—	5
Meralco or electric cooperative	8	2	16	10	16	8	2	11	73
Retail electricity supplier (RES)	—	3	4	6	1	—	1	—	15
Direct from generation company (directly connected to NGCP)	—	1	1	2	1	1	—	—	6
Self-generation	—	—	1	—	—	—	—	—	1
Others	—	—	—	—	2	—	—	1	3
Total	8	6	25	20	20	9	3	12	103

Note: This question was only asked in the primary survey, hence the total number of firms that responded is only 100.

Table III-10. Summary statistics on water consumption and expenditure

Statistic	Q44-46. For the past 3 months, what was your average monthly water consumption in cubic meters?	Q47-49. For the past 3 months, how much did you spend for monthly water in pesos?
N	115	115
Mean	13,478.76	191,304.8
Standard deviation	30,277.76	585,432.1
Minimum	5,000.5	50,000.5
Maximum	50,000.5	5,500,001

Note: Question 44 is a filter question. Depending on the chosen response option in Question 44, the respondent is redirected to either Question 45 or Question 46 where he/she is asked to provide a more specific response. The same logic applies to Questions 47 to 49.

Table III-11. Percentage and number of firms per water rate interval

Q50. How much (in pesos) do you pay per cubic meter of water?	%	N
Less than 40.00	2.00	2
40.01 to 50.00	0.00	0
50.01 to 60.00	1.00	1
60.01 to 70.00	0.00	0
70.01 to 80.00	1.00	1
80.01 to 90.00	0.00	0
90.01 to 100.00	0.00	0
More than 100.00	0.00	0
No response	96.00	96
Total	100.00	100

Note: This question was only asked in the primary survey, hence the total number of responses is only 100.

Table III-12. Number of firms by consideration in switching electricity provider, by degree of importance

Q51. If you can switch to another electricity provider, what are your considerations? With 1 being the most important, rank the following.	1	2	3	4	No response	N
Price	42	30	13	25	5	115
Supply stability and reliability	43	42	20	6	4	115
Safety and security	18	26	45	20	6	115
Environmental concerns	9	13	32	56	5	115

Figure III-1. Percentage and number of firms participating in PEZA energy-related initiatives

Q52. Have you participated in any PEZA-organized energy efficiency initiatives designed to improve operational efficiency and increase competitiveness?

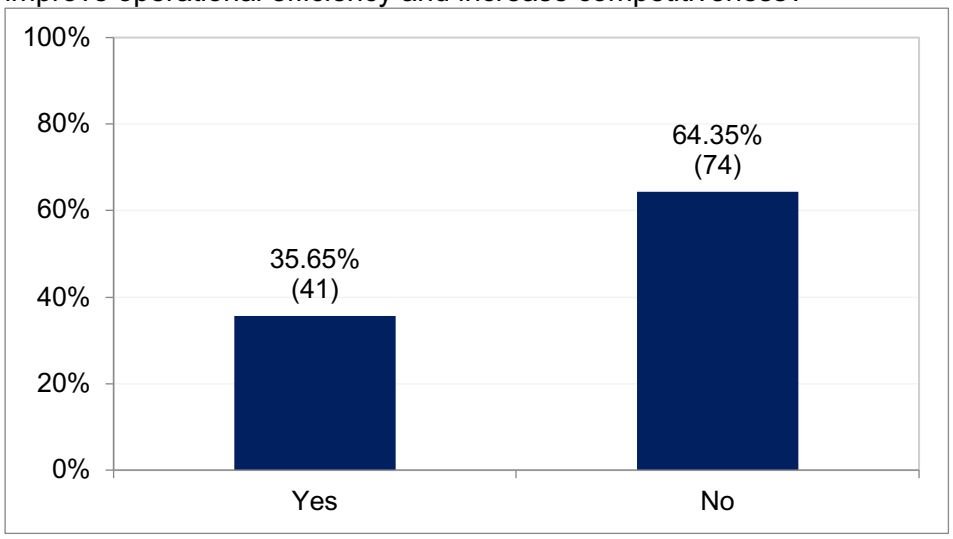

Figure III-2. Percentage and number of firms aware of PEZA energy-related programs, by program

Q53. Are you aware of any of the following?

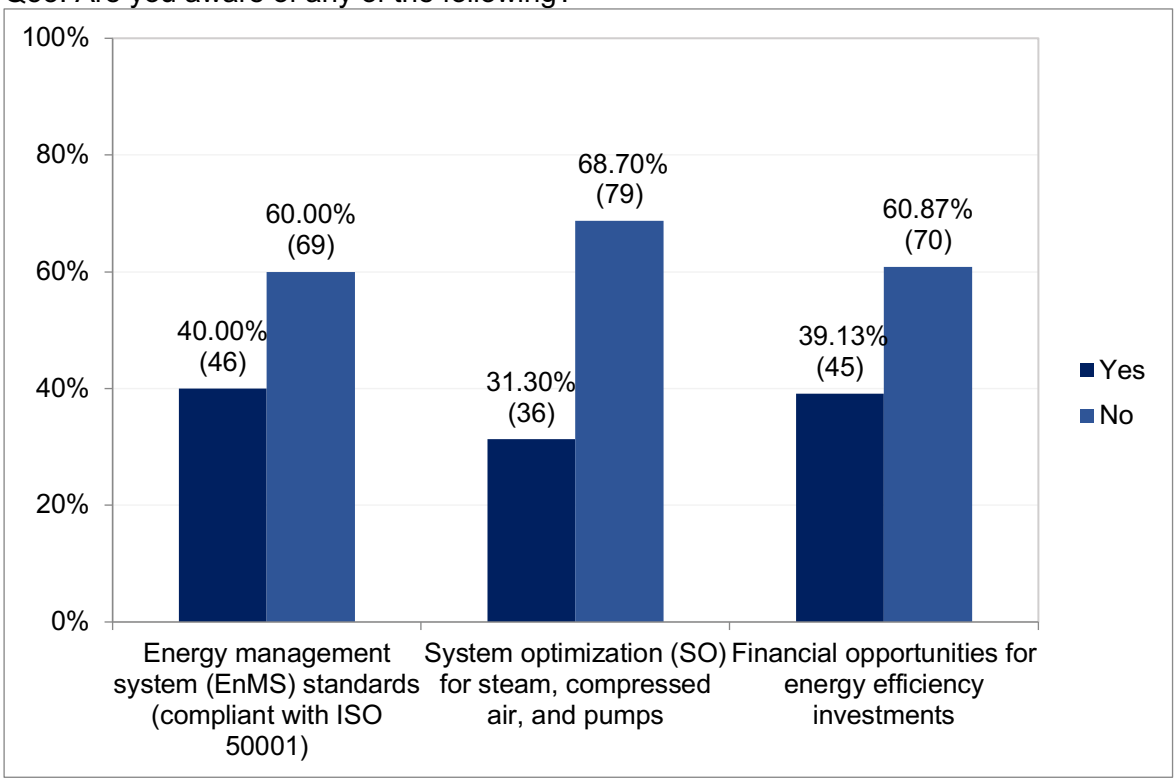

Figure III-3. Percentage and number of firms implementing energy conservation measures

Q54. Has your company implemented any measures to reduce energy consumption and cost?

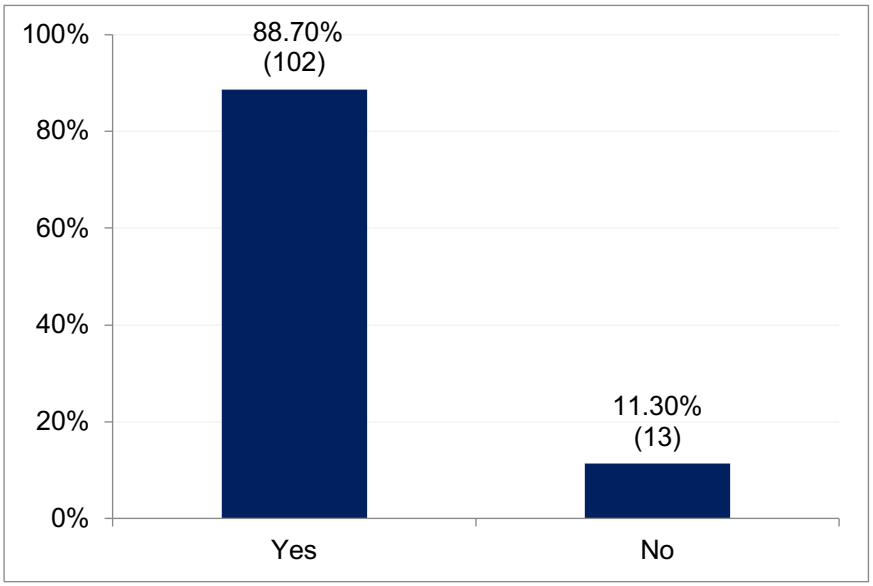

Table III-13. Number of firms implementing energy conservation measures, by measure

Q55. Which of the following measures has your company implemented to reduce energy consumption and cost?	N
Installing solar panels	10
Using inverter-type air conditioners	65
Installing LED lighting	92
Other	25
Total	102

Table III-14. Number of firms by reason for not implementing energy conservation measures, by degree of importance

Q56. With 1 being the most important, rank the following reasons for not implementing or adopting any energy efficiency and conservation measures for operational efficiency.	1	2	3	4	5	N
Lack of understanding of the management on the advantages of adopting energy efficiency measures	4	3	0	3	3	13
Lack of technical knowledge on how to implement energy efficient measures	2	2	5	3	1	13
Lack of resources to switch to energy efficient equipment	3	6	4	0	0	13
Energy efficiency not a company priority	1	2	2	2	6	13
No perceived substantial effect in adopting energy efficient measures	3	0	2	5	3	13

SECTION IV – Fuels Used in Production

This section covers information on production fuel mix; and importance, use, consumption, and expenditure on different types of fuel in main production processes. This section covers Questions 57 to 130 of the survey questionnaire.

However, Question 57, 122, and 128 are not included in this section. Question 57 is a filter question of Section IV. If the respondent answers "Yes" in Question 57, he/she proceeds to the next question. If the respondent answers "No," he/she skips Section IV and is redirected to Section V.

Furthermore, Questions 122 and 128 ask for daily consumption of other fuels. Responses are in different units and cannot be aggregated.

The different fuel types are defined as follows: *Bunker fuel* is the generic term to any fuel used to power ships' engines.[1] *Biodiesel* is an alternative fuel that is produced from vegetable or animal oil and/or waste cooking oil.[2] *Coal* is fuel from the remains of prehistoric vegetation that accumulated in swamps and peat bogs.[3] *Diesel* is fuel refined from crude oil at petroleum refineries and is used in motor vehicles.[4] *Gasoline* is made from crude oil and other petroleum liquids and is mainly used as an engine fuel in vehicles.[5] *Kerosene* is a liquid chemical mixture produced in the distillation of crude oil.[6] *Liquefied petroleum gas (LPG)* is a mixture of flammable hydrocarbon gases such as propane and butane commonly used for home heating, cooking, hot water, and vehicles.[7] *Natural gas* is a fossil energy source that formed deep beneath the earth's surface and is largely composed of methane.[8] *Propane* is a nontoxic, colorless, and odorless compressed gas similar to LPG and stored as liquid used for heating, cooking, and fuel for engine applications.[9]

Each production process is defined as follows: *Air compression (or vacuuming)* is the process of compressing gas to another higher-pressured gas which can be used to do mechanical work. Generally, air compressors are powered by electric motor, diesel, or gas engine.[10] *Air or gas mixing* is the process of mixing gases for industrial and scientific purposes where the composition of the resulting mixture is specified and controlled.[11] *Baking (or baking out)* refers to the process of using high-heat temperature to remove volatile compounds from materials and objects before placing them into situations where the slow release of the same volatile compounds would contaminate the contents of the container or vessel.[12] *Boiler operation* is the process of using a boiler which is a pressurized vessel designed to heat water or produce steam which can be used to provide heating.[13] *Burning or combustion* is a high-temperature chemical reaction between a fuel and an oxidant (e.g. oxygen) that produces oxidized gaseous products (i.e. smoke).[14] *Curing* refers to the process of applying heat to a material thus

[1] http://www.mckinseyenergyinsights.com/resources/refinery-reference-desk/bunker-fuel/
[2] http://www.esru.strath.ac.uk/EandE/Web_sites/02-03/biofuels/what_biodiesel.htm
[3] https://www.worldcoal.org/coal/what-coal
[4] https://www.eia.gov/energyexplained/diesel-fuel/
[5] https://www.eia.gov/energyexplained/gasoline/
[6] https://energyeducation.ca/encyclopedia/Kerosene
[7] https://www.originenergy.com.au/blog/what-is-lpg/
[8] https://www.eia.gov/energyexplained/natural-gas/
[9] https://propane.com/about/about-propane/what-is-propane-gas/
[10] https://www.mechanicalbooster.com/2018/03/what-is-air-compressor-and-its-types.html
[11] https://en.wikipedia.org/wiki/Gas_blending
[12] https://en.wikipedia.org/wiki/Bake-out
[13] https://betterbricks.com/resources/boilers
[14] https://en.wikipedia.org/wiki/Combustion#cite_note-1

creating a chemical reaction.[15] *Die casting* is a metal casting process that utilizes metal mold or dies[16], while *wire bonding* is a connection technique used to electrically connect microchips to terminals of chip packages.[17] *Drying* is the final thermal treatment in a production process that removes remaining solvent on a surface[18], while *annealing* is the heating of an object to a single-phase field followed by equilibrium cooling.[19] *Engine loading (or preparation)* is the process of applying an external resistance called load in which a machine such as a motor or engine acts.[20] *Fabrication (of metal)* is the process of building machines and structures from raw materials.[21] *Forklift operation* refers to the operation of forklift[22] – a vehicle with two bars in the front for moving and lifting heavy goods – in production processes. *Heat treatment* or *metal treatment/pre-treatment* is the combination of timed heating and cooling of metals or alloys to produce desirable properties.[23] *Ice making* is the process of making ice, often part of refrigeration.[24] *Impregnation* refers to the sealing of porosity of workpieces in manufacturing.[25] *Machine injection or molding* involves inserting melted plastic into a mold cavity to form specific shapes.[26] *Melting or pre-melting* is the process of overheating solid materials until they liquefy.[27] *Painting* refers to the process of coating film on a surface of an object for protection and aesthetics.[28] *Smelting* is the production of metal from ore/concentrate.[29] *Stamping* is manufacturing process of converting flat metal sheets into specific shapes.[30] *Steel or metal cutting* refers to the manufacturing process of cutting metal or steel into specific shapes.[31] *Thermal oxidation* is the process of burning organic vapor in an effluent air stream from a metal casting pit before it is released into the atmosphere.[32] *Transportation* is the movement of goods from one place to another, while *logistics* is the planning and organizing of goods to make sure that resources are in the right places. [33] *Welding* is a technique used to join metallic parts through heat application.

[15] https://www.wisoven.com/processes/curing
[16] https://www.sciencedirect.com/topics/materials-science/die-casting
[17] https://www.sciencedirect.com/topics/engineering/bonding-wire
[18] https://www.sciencedirect.com/topics/engineering/drying-process
[19] https://www.sciencedirect.com/topics/materials-science/annealing
[20] https://en.wikipedia.org/wiki/Mechanical_load#cite_note-1
[21] https://www.thefabricator.com/thefabricator/article/shopmanagement/what-is-metal-fabrication-and-where-is-the-industry-headed-
[22] https://dictionary.cambridge.org/dictionary/english/forklift
[23] https://www.sciencedirect.com/topics/engineering/heat-treatment
[24] https://www.collinsdictionary.com/dictionary/english/ice-maker
[25] https://i-e.cz/impregnation
[26] http://www.automaticplastics.com/understanding-the-injection-moulding-process/
[27] https://thermcraftinc.com/how-does-a-melting-furnace-work/
[28] https://www.nite.go.jp/data/000007636.pdf
[29] https://www.sciencedirect.com/topics/earth-and-planetary-sciences/smelting
[30] https://www.esict.com/what-is-metal-stamping/
[31] https://www.sciencedirect.com/topics/engineering/metal-cutting
[32] https://www.sciencedirect.com/topics/engineering/thermal-oxidation
[33] https://dictionary.cambridge.org/us/dictionary/english/transportation

Table IV-1. Mean share of fuel in production fuel mix

Q58. What is your estimated production fuel mix?	Mean (%)
Electricity	82.07
Diesel	14.87
Gasoline	7.35
Kerosene	2.09
LPG	7.00
Other fuel (1)	5.71
Other fuel (2)	0.98
Other fuel (3)	3.36

Note: This question was only asked in the primary survey. Other fuel options include biodiesel, bunker, coal, natural gas, and propane.

Table IV-2. Percentage and number of firms using fuel, by fuel

Q59 / 65 / 71 / 77 / 83 / 89 / 95 / 101 / 107. Do you use biodiesel / bunker / coal / diesel / gasoline / kerosene / LPG / natural gas / propane in your main production processes?

	Biodiesel	Bunker	Coal	Diesel	Gasoline	Kerosene	LPG	Natural gas	Propane
Yes	0.00	0.00	0.00	39.29	17.86	8.93	39.29	5.36	3.57
	0	0	0	22	10	5	22	3	2
No	100.00	100.00	100.00	60.71	82.14	91.07	60.71	94.64	96.43
	56	56	56	34	46	51	34	53	54
Total	100.00	100.00	100.00	100.00	100.00	100.00	100.00	100.00	100.00
	56	56	56	56	56	56	56	56	56

Notes: Question 59 is a filter question. If the respondent answers “Yes” in Question 59, he/she proceeds to the next question. If the respondent answers “No,” he/she skips the follow-up questions (i.e. Questions 60 to 64) and is redirected to Question 65. The same logic applies to Questions 65, 71, 77, 83, 89, 95, 101, and 107; All respondents answered “No” in filter Questions 59, 65, and 71 on the use of biodiesel, bunker, and fuel, respectively. Follow-up Questions 60 to 64, 66 to 70, and 72 to 76 recorded no response.

Table IV-3. Percentage and number of firms using fuel, by degree of importance and fuel

Q78 / 84 / 90 / 96 / 102 / 108. How important is diesel / gasoline / kerosene / LPG / natural gas / propane in your main production processes?

	Diesel	Gasoline	Kerosene	LPG	Natural gas	Propane
Slightly important	13.64	10.00	20.00	4.55	33.33	0.00
	3	1	1	1	1	0
Important	45.45	50.00	60.00	40.91	33.33	50.00
	10	5	3	9	1	1
Fairly important	9.09	0.00	0.00	4.55	0.00	0.00
	2	0	0	1	0	0
Very important	31.82	40.00	20.00	50.00	33.33	0.00
	7	4	1	11	1	0
No response	0.00	0.00	0.00	0.00	0.00	50.00
	0	0	0	0	0	1
Total	100.00	100.00	100.00	100.00	100.00	100.00
	22	10	5	22	3	2

Notes: “Not important” was also provided as an option but was not chosen by any locator; Question 78 is only asked to locators that responded “Yes” in Question 77. The same logic applies to Questions 83 and 84, 89 and 90, 95 and 96, 101 and 102, and 107 and 108.

Table IV-4. Number of firms involving each production process, by fuel

Q36 / 79 / 85 / 91 / 97 / 103 / 109. Which of the following processes involved in your main production use electricity / diesel / gasoline / kerosene / LPG / natural gas / propane?

	Electricity	Diesel	Gasoline	Kerosene	LPG	Natural gas	Propane
With heating component							
Fabrication	40	1	1	0	3	1	0
Welding	34	0	1	0	2	0	0
Machine injection or molding	33	0	0	0	0	0	0
Curing (e.g. oven curing, powder paint curing, etc.)	24	0	0	0	2	0	0
Drying or annealing (e.g. oven drying, mold drying, core drying, air handling, etc.)	24	0	0	0	2	0	0
Heat treatment	19	1	0	0	7	0	1
Standby or back-up power generation	12	0	0	0	0	0	0
Die casting or wire bonding	10	0	0	1	2	0	0
Boiler operation (e.g. for steam generation, etc.)	9	3	0	0	2	0	0
Melting or pre-melting	7	0	0	0	6	0	0
Burning	6	0	0	0	1	0	0
Power generation	6	0	0	0	0	0	0
Baking	2	0	0	0	0	0	0
Impregnation	2	0	0	0	1	0	0
Smelting	1	0	0	0	1	0	0
Thermal oxidation	0	0	0	0	0	0	0
Without heating component							
Air conditioning	94	0	0	0	0	0	0
Air compression/ vacuuming	72	1	2	1	1	1	1
Forklift operation	40	11	2	0	0	0	0
Other (cleaning of machine parts, threading)	38	4	1	3	3	2	0
Transportation and logistics (e.g. trucking, distribution, delivery, etc.)	25	10	4	0	0	0	0
Painting	15	0	1	0	0	0	0
Steel cutting	15	0	1	0	3	0	0
Stamping	12	1	0	0	0	0	0
Engine loading or preparation	8	1	0	0	0	0	0
Air/gas mixing (e.g. Selas mixing, etc.)	3	0	1	0	0	0	0
Ice making	1	0	0	0	0	0	0

Notes: All other options that were provided but were not chosen are baking, ice making, machine injection or molding, and thermal oxidation; Question 79 is only asked to locators that responded “Yes” in Question 77. The same logic applies to Questions 83 and 85, 89 and 91, 95 and 97, 101 and 103, and 107 and 109.

Ravago, Majah-Leah; Fabella, Raul; Jandoc, Karl Robert; Frias, Renzi; Magadia, J. Kathleen (2021), “Survey Data on Energy and Fuel Use of Firms in Economic Zones in the Philippines”, Data in Brief.

Table IV-5. Percentage and number of firms by mode of procurement and fuel

Q82 / 88 / 94 / 100 / 106 / 112. How do you procure your diesel / gasoline / kerosene / LPG / natural gas / propane?

	Diesel	Gasoline	Kerosene	LPG	Natural gas	Propane
	%	%	%	%	%	%
Contracted	4.55	0.00	0.00	22.73	33.33	0.00
	1	0	0	5	1	0
Buying as needed	81.82	80.00	80.00	54.55	0.00	50.00
	18	8	4	12	0	1
Other	0.00	0.00	0.00	0.00	0.00	0.00
	0	0	0	0	0	0
No response	13.64	20.00	20.00	22.73	66.67	50.00
	3	2	1	5	2	1
Total	100.00	100.00	100.00	100.00	100.00	100.00
	22	10	5	22	3	2

Table IV-6. Number of firms using diesel, by production process, consumption level, and expenditure level

	Q80. How much diesel do you consume (in liters) for each process per day?						Q81. How much do you spend for your diesel consumption (in pesos) for each process per month?						
	1 – 200	201 – 400	401 – 600	601 – 800	801 – 1K	1K & up	1 – 270K	270K – 540K	540K – 810K	810K – 1.08M	1.08M – 1.35M	1.35M & up	N
With heating component													
Boiler operation (e.g. for steam generation, etc.)	2	0	0	0	0	1	2	0	0	0	0	1	3
Fabrication	1	0	0	0	0	0	1	0	0	0	0	0	1
Heat treatment	0	0	0	0	0	1	0	0	0	0	0	1	1
Without heating component													
Forklift operation	9	1	1	0	0	0	11	0	0	0	0	0	11
Transportation and logistics (e.g. trucking, distribution, delivery, etc.)	9	1	0	0	0	0	10	0	0	0	0	0	10
Other	3	1	0	0	0	0	4	0	0	0	0	0	4
Engine loading or preparation	0	1	0	0	0	0	1	0	0	0	0	0	1
Stamping	1	0	0	0	0	0	1	0	0	0	0	0	1
Air compression/vacuuming	1	0	0	0	0	0	1	0	0	0	0	0	1

Note: All other options that were provided but were not chosen are air/gas mixing (e.g. selas mixing, etc.), baking, burning, curing (e.g. oven curing, powder paint curing, etc.), die casting or wire bonding, drying or annealing (e.g. oven drying, mold drying, core drying, air handling, etc.), ice making, impregnation, machine injection or molding, melting or pre-melting, metal treatment or pre-treatment, painting, smelting, steel cutting, thermal oxidation, and welding.

Table IV-7. Number of firms using gasoline, by production process, consumption level, and expenditure level

	Q86. How much gasoline do you consume (in liters) for each process per day?						Q87. How much do you spend for your gasoline consumption (in pesos) for each process per month?						
	1 – 200	201 – 400	401 – 600	601 – 800	801 – 1K	1K & up	1 – 360K	360K – 720K	720K – 1.08M	1.08M – 1.44M	1.44M – 1.8M	1.8M & up	N
With heating component													
Fabrication	1	0	0	0	0	0	1	0	0	0	0	0	1
Welding	0	0	0	1	0	0	0	1	0	0	0	0	1
Without heating component													
Transportation and logistics (e.g. trucking, distribution, delivery, etc.)	4	0	0	0	0	0	4	0	0	0	0	0	4
Forklift operation	2	0	0	0	0	0	2	0	0	0	0	0	2
Air compression/vacuuming	1	0	1	0	0	0	1	1	0	0	0	0	2
Air/gas mixing (e.g. Selas mixing, etc.)	0	1	0	0	0	0	0	1	0	0	0	0	1
Painting	0	1	0	0	0	0	0	1	0	0	0	0	1
Steel cutting	0	0	0	1	0	0	0	1	0	0	0	0	1
Other	0	1	0	0	0	0	1	0	0	0	0	0	1

Note: All other options that were provided but were not chosen are baking, boiler operation (e.g. for steam generation, etc.), burning, curing (e.g. oven curing, powder paint curing, etc.), die casting or wire bonding, drying or annealing (e.g. oven drying, mold drying, core drying, air handling, etc.), engine loading or preparation, heat treatment, ice making, impregnation, machine injection or molding, melting or pre-melting, metal treatment or pre-treatment, smelting, stamping, and thermal oxidation.

Table IV-8. Number of firms using kerosene, by production process, consumption level, and expenditure level

	Q92. How much kerosene do you consume (in liters) for each process per day?						Q93. How much do you spend for your kerosene consumption (in pesos) for each process per month?						
	1 – 200	201 – 400	401 – 600	601 – 800	801 – 1K	1K & up	1 – 300K	300K – 600K	600K – 900K	900K – 1.2M	1.2M – 1.5M	1.5M & up	N
With heating component													
Die casting or wire bonding	0	1	0	0	0	0	1	0	0	0	0	0	1
Without heating component													
Other (cleaning of machine parts, threading)	3	0	0	0	0	0	3	0	0	0	0	0	3
Air compression/vacuuming	0	1	0	0	0	0	0	1	0	0	0	0	1

Note: All other options that were provided but were not chosen are air/gas mixing (e.g. selas mixing, etc.), baking, boiler operation (e.g. for steam generation, etc.), burning, curing (e.g. oven curing, powder paint curing, etc.), drying or annealing (e.g. oven drying, mold drying, core drying, air handling, etc.), engine loading or preparation, fabrication, forklift operation, heat treatment, ice making, impregnation, machine injection or molding, melting or pre-melting, metal treatment or pre-treatment, painting, smelting, welding, stamping, steel cutting, thermal oxidation, and transportation and logistics (e.g. trucking, distribution, delivery, etc.).

Table IV-9. Number of firms using LPG, by production process, consumption level, and expenditure level

	Q98. How much LPG do you consume (in kilograms) for each process per month?							Q99. How much do you spend for your LPG consumption (in pesos) for each process per month?						
	1 – 20K	20K – 40K	40K – 60K	60K – 80K	80K – 100K	100K & up	No response	1 – 1M	1M – 2M	2M – 3M	3M – 4M	4M – 5M	5M & up	N
Without heating component														
Heat treatment	6	1	0	0	0	0	0	7	0	0	0	0	0	7
Melting or pre-melting	5	0	1	0	0	0	0	5	1	0	0	0	0	6
Fabrication	2	1	0	0	0	0	0	3	0	0	0	0	0	3
Other (brazing, preheating, sanding)	3	0	0	0	0	0	0	3	0	0	0	0	0	3
Boiler operation (e.g. for steam generation, etc.)	1	0	0	0	0	1	0	1	0	0	0	0	1	2
Curing (e.g. oven curing, powder paint curing, etc.)	1	0	0	0	1	0	0	1	0	0	0	0	1	2
Die casting or wire bonding	1	0	0	0	0	0	1	1	1	0	0	0	0	2
Drying or annealing (e.g. oven drying, mold drying, core drying, air handling, etc.)	2	0	0	0	0	0	0	2	0	0	0	0	0	2
Welding	1	1	0	0	0	0	0	2	0	0	0	0	0	2
Burning	1	0	0	0	0	0	0	0	0	0	0	0	0	1
Impregnation	1	0	0	0	0	0	0	0	0	0	0	0	0	1
Metal treatment or pre-treatment	1	0	0	0	0	0	0	0	0	0	0	0	0	1
Smelting	1	0	0	0	0	0	0	0	0	0	0	0	0	1

Without heating component														
Steel cutting	2	1	0	0	0	0	0	3	0	0	0	0	0	3
Air compression/vacuuming	0	1	0	0	0	0	0	0	0	0	0	0	0	1

Note: All other options that were provided but were not chosen are air/gas mixing (e.g. selas mixing, etc.), baking, engine loading or preparation, forklift operation, ice making, machine injection or molding, painting, stamping, thermal oxidation, and transportation and logistics (e.g. trucking, distribution, delivery, etc.).

Table IV-10. Number of firms using natural gas, by production process, consumption level, and expenditure level

	Q104. How much natural gas do you consume (in million standard cubic feet per day or mmscfd) for each process per day?						Q105. How much do you spend for your natural gas consumption (in pesos) for each process per month?						
	1 – 20	21 – 40	41 – 60	61 – 80	81 – 100	101 & up	1 – 1M	1M – 2M	2M – 3M	3M – 4M	4M – 5M	5M & up	N
With heating component													
Other (brazing, calibration of gauges)	1	1	0	0	0	0	2	0	0	0	0	0	2
Fabrication	1	0	0	0	0	0	1	0	0	0	0	0	1
Without heating component													
Air compression/ vacuuming	0	1	0	0	0	0	0	1	0	0	0	0	1

Note: All other options that were provided but were not chosen are air/gas mixing (e.g. selas mixing, etc.), baking, boiler operation (e.g. for steam generation, etc.), burning, curing (e.g. oven curing, powder paint curing, etc.), die casting or wire bonding, drying or annealing (e.g. oven drying, mold drying, core drying, air handling, etc.), engine loading or preparation, forklift operation, heat treatment, ice making, impregnation, machine injection or molding, melting or pre-melting, metal treatment or pre-treatment, painting, smelting, welding, stamping, steel cutting, thermal oxidation, and transportation and logistics (e.g. trucking, distribution, delivery, etc.).

Table IV-11. Number of firms using propane, by production process, consumption level, and expenditure level

	Q110. How much propane do you consume (in kilograms) for each process per month?						Q111. How much do you spend for your propane consumption (in pesos) for each process per month?						
	1 - 20,000	20,001 - 40,000	40,001 - 60,000	60,001 - 80,000	80,001 - 100,000	100,001 & up	1 - 1 million	1,000,001 - 2 million	2,000,001 - 3 million	3,000,001 - 4 million	4,000,001 - 5 million	5,000,001 & up	N
With heating component													
Heat treatment	1	0	0	0	0	0	1	0	0	0	0	0	1
Without heating component													
Air compression/ vacuuming	1	0	0	0	0	0	1	0	0	0	0	0	1

Note: All other options that were provided but were not chosen are air/gas mixing (e.g. selas mixing, etc.), baking, boiler operation (e.g. for steam generation, etc.), burning, curing (e.g. oven curing, powder paint curing, etc.), die casting or wire bonding, drying or annealing (e.g. oven drying, mold drying, core drying, air handling, etc.), engine loading or preparation, fabrication, forklift operation, ice making, impregnation, machine injection or molding, melting or pre-melting, metal treatment or pre-treatment, painting, smelting, welding, stamping, steel cutting, thermal oxidation, transportation and logistics (e.g. trucking, distribution, delivery, etc.), and other.

Table IV-12. Percentage and number of firms using other fuel (1)

Q113. Do you use other fuel (1)* in your main production processes that was not mentioned in the previous questions?	%	N
Yes	12.50	7
No	87.50	49
Total	100.00	56

Note: *electricity, hydrogen, biomass, thuban, argon, rice hull, hydraulic oil

Table IV-13. Percentage and number of firms using other fuel (1), by degree of importance

Q114. How important is other fuel (1) in your main production processes?	%	N
Not important	0.00	0
Slightly important	0.00	0
Important	28.57	2
Fairly important	28.57	2
Very important	42.86	3
Total	100.00	7

Table IV-14. Number of firms using other fuel (1), by production process

Q115. Which of the following processes involved in your main production use other fuel (1)?	N
Air compression/vacuuming	3
Boiler operation (e.g. for steam generation, etc.)	2
Other (casting, mechanical)	2
Drying or annealing (e.g. oven drying, mold drying, core drying, air handling, etc.)	1
Fabrication	1
Forklift operation	1
Machine injection or molding	1

Table IV-15. Mean expenditure of firms for other fuel (1), by production process

Q117. How much do you spend for your other fuel (1) consumption for each process per month?	Mean (pesos)	N
Air compression/vacuuming	6,000	3
Boiler operation (e.g. for steam generation, etc.)	21,500	2
Other (casting, mechanical)	17,600	2
Drying or annealing (e.g. oven drying, mold drying, core drying, air handling, etc.)	934,673	1
Fabrication	40,000	1
Forklift operation	15,000	1
Machine injection or molding	366	1

Ravago, Majah-Leah; Fabella, Raul; Jandoc, Karl Robert; Frias, Renzi; Magadia, J. Kathleen (2020), "Survey Data on Energy and Fuel Use of Firms in Economic Zones in the Philippines", Data in Brief.

Table IV-16. Percentage and number of firms using other fuel (1), by mode of procurement

Q118. How do you procure your other fuel (1)?	%	N
Contracted	14.29	1
Buying as needed	57.14	4
Other	28.57	2
Total	100.00	7

Table IV-17. Percentage and number of firms using other fuel (2)

Q119. Do you use other fuel (2)* in your main production processes that was not mentioned in the previous questions?	%	N
Yes	14.29	1
No	85.71	6
Total	100.00	7

Note: *engine fuel

Table IV-18. Percentage and number of firms using other fuel (2), by degree of importance

Q120. How important is other fuel (2) in your main production processes?	%	N
Not important	0.00	0
Slightly important	0.00	0
Important	100.00	1
Fairly important	0.00	0
Very important	0.00	0
Total	100.00	1

Table IV-19. Number of firms using other fuel (2), by production process

Q121. Which of the following processes involved in your main production use other fuel (2)?	N
Forklift operation	1
Other (motor pool and maintenance)	1

Table IV-20. Mean expenditure of firms for other fuel (2), by production process

Q123. How much do you spend for your other fuel (2) consumption for each process per month?	Mean (pesos)	N
Forklift operation	25000	1
Other (motor pool and maintenance)	25000	1

Ravago, Majah-Leah; Fabella, Raul; Jandoc, Karl Robert; Frias, Renzi; Magadia, J. Kathleen (2020), "Survey Data on Energy and Fuel Use of Firms in Economic Zones in the Philippines", Data in Brief.

Table IV-21. Percentage and number of firms using other fuel (2), by mode of procurement

Q124. How do you procure your other fuel (2)?	%	N
Contracted	0.00	0
Buying as needed	100.00	1
Other	0.00	0
Total	100.00	1

Table IV-22. Percentage and number of firms using other fuel (3)

Q125. Do you use other fuel (3)* in your main production processes that was not mentioned in the previous questions?	%	N
Yes	100.00	1
No	0.00	0
Total	100.00	1

Note: *hydraulic oil

Table IV-23. Percentage and number of firms using other fuel (3), by degree of importance

Q126. How important is other fuel (3) in your main production processes?	%	N
Not important	0.00	0
Slightly important	0.00	0
Important	100.00	1
Fairly important	0.00	0
Very important	0.00	0
Total	100.00	1

Table IV-24. Percentage and number of firms using other fuel (3), by production process

Q127. Which of the following processes involved in your main production use other fuel (3)?	%	N
Forklift operation	100.00	1
Other (loader/bogie train/motor pool/shiplift/transporter)	100.00	1

Ravago, Majah-Leah; Fabella, Raul; Jandoc, Karl Robert; Frias, Renzi; Magadia, J. Kathleen (2020), "Survey Data on Energy and Fuel Use of Firms in Economic Zones in the Philippines", Data in Brief.

Table IV-25. Mean expenditure of firms for other fuel (3), by production process

Q129. How much do you spend for your other fuel (3) consumption for each process per month?	Mean (pesos)	N
Forklift operation	22,000	1
Other (loader/bogie train/motor pool/shiplift/transporter)	28,000	1

Table IV-26. Percentage and number of firms using other fuel (3), by mode of procurement

Q130. How do you procure your other fuel (3)?	%	N
Contracted	0.00	0
Buying as needed	100.00	1
Other	0.00	0
Total	100.00	1

Ravago, Majah-Leah; Fabella, Raul; Jandoc, Karl Robert; Frias, Renzi; Magadia, J. Kathleen (2020), “Survey Data on Energy and Fuel Use of Firms in Economic Zones in the Philippines”, Data in Brief.

SECTION V – Aptitude on Alternative Fuels and Primary Energies

This section covers information on knowledge, considerations, and opinions on alternative fuels (natural gas) and primary energies (solar and wind), and their experiences in using them. Natural gas in this section is defined the same as in the previous questions. On the other hand, solar energy is energy from the sun that is converted to thermal or electrical energy,[34] while wind energy from the airflows that is also converted to the same energies.[35] This section covers Questions 131 to 169 of the survey questionnaire.

However, Questions 142, 155, and 168 are not included in this section due to inconsistencies.

Table V-1. Summary statistics and percentage of firms on extent of knowledge, by fuel/source

Q131 / 144 / 157. With 1 being limited, and 5 being advanced, what is the extent of your knowledge on natural gas as fuel / solar as primary energy / wind as primary energy?

Extent of knowledge	Natural Gas	Solar	Wind
1 (Limited)	44.35%	13.91%	36.52%
2	14.78%	13.04%	18.26%
3	29.57%	38.26%	26.09%
4	9.57%	26.09%	15.65%
5 (Advanced)	1.74%	8.70%	3.48%
Weighted Mean	2.10	3.03	2.31
N	115	115	115

Table V-2. Percentage and number of firms on safety of alternative fuels and primary energies, by fuel/source

Q132 / 145 / 158. Do you think natural gas / solar / wind is safe to utilize as fuel / primary energy in your production process?

	Natural Gas		Solar		Wind	
	%	N	%	N	%	N
Yes	57.39	66	86.09	99	58.26	67
No	42.61	49	13.91	16	41.74	48
Total	100.00	115	100.00	115	100.00	115

[34] https://www.seia.org/initiatives/about-solar-energy
[35] https://www.awea.org/wind-101/basics-of-wind-energy

Ravago, Majah-Leah; Fabella, Raul; Jandoc, Karl Robert; Frias, Renzi; Magadia, J. Kathleen (2020), "Survey Data on Energy and Fuel Use of Firms in Economic Zones in the Philippines", Data in Brief.

Table V-3. Percentage and number of firms on cost-competitiveness of alternative fuels and primary energies, by fuel/source

Q133 / 146 / 159. Do you think natural gas / solar / wind is cost-competitive relative to the fuels and primary energies you are currently using?

	Natural Gas		Solar		Wind	
	%	N	%	N	%	N
Yes	65.22	75	79.13	91	53.91	62
No	34.78	40	20.87	24	46.09	53
Total	100.00	115	100.00	115	100.00	115

Table V-4. Percentage and number of firms on savings in alternative fuels and primary energies, by saving interval and fuel/source

Q134 / 147 / 160. How much (in pesos) do you think can you save if you use natural gas / solar / wind?

	Natural Gas		Solar		Wind	
Interval	%	N	%	N	%	N
1 to 200,000	83.48	96	53.04	61	68.70	79
200,001 to 400,000	6.96	8	20.87	24	10.43	12
400,001 to 600,000	6.96	8	7.83	9	10.43	12
600,001 to 800,000	0.87	1	5.22	6	0.87	1
800,001 to 1,000,000	0.00	0	2.61	3	1.74	2
1,000,001 and above	1.74	2	10.43	12	7.83	9
Total	100.00	115	100.00	115	100.00	115

Ravago, Majah-Leah; Fabella, Raul; Jandoc, Karl Robert; Frias, Renzi; Magadia, J. Kathleen (2020), “Survey Data on Energy and Fuel Use of Firms in Economic Zones in the Philippines”, Data in Brief.

Table V-5. Number of firms by consideration in using alternative fuels and primary energies in production, by degree of importance and fuel/source

Q135 / 148 / 161. In case natural gas / solar / wind would be made available to you, what would be your considerations in using it in your production processes? With 1 being the most important, rank the following.

	Natural gas							Solar							Wind						
	1	2	3	4	5	6	Mean	1	2	3	4	5	6	Mean	1	2	3	4	5	6	Mean
Retrofitting costs of equipment	5	13	17	21	24	24	2.87	4	11	17	17	26	25	2.75	2	12	13	24	27	22	2.72
Environmental concerns	17	18	12	11	18	28	3.24	17	13	9	12	22	27	3.10	15	17	10	10	21	27	3.14
Supply stability and reliability	10	18	16	28	19	13	3.36	18	17	25	17	12	11	3.79	23	17	22	16	7	15	3.88
Price	18	20	18	14	12	22	3.54	23	20	17	14	8	18	3.82	23	19	21	11	8	18	3.84
Safety and security	15	22	26	17	18	5	3.84	8	19	14	27	23	9	3.35	9	14	17	22	28	10	3.24
Compatibility of machines and equipment	40	16	16	13	9	8	4.40	30	20	18	13	9	10	4.19	28	21	17	17	9	8	4.18
No response	10	8	10	11	15	15		15	15	15	15	15	15		15	15	15	15	15	15	
Total	115	115	115	115	115	115		115	115	115	115	115	115		115	115	115	115	115	115	

Ravago, Majah-Leah; Fabella, Raul; Jandoc, Karl Robert; Frias, Renzi; Magadia, J. Kathleen (2020), “Survey Data on Energy and Fuel Use of Firms in Economic Zones in the Philippines”, Data in Brief.

Table V-6. Percentage and number of firms on retrofitting cost of equipment, by fuel/source

Q136 / 149 / 162. How much (in pesos) do you think will the retrofitting of equipment cost?						
	Natural gas		Solar		Wind	
Cost interval	%	N	%	N	%	N
1 to 200,000	45.22	52	36.52	42	34.78	40
200,001 to 400,000	10.43	12	18.26	21	20.00	23
400,001 to 600,000	6.96	8	6.96	8	6.09	7
600,001 to 800,000	5.22	6	3.48	4	2.61	3
800,001 to 1,000,000	4.35	5	4.35	5	0.87	1
1,000,001 and above	22.61	26	26.96	31	29.57	34
No response	5.22	6	3.48	4	6.09	7
Total	100.00	115	100.00	115	100.00	115

Table V-7. Percentage and number of firms open to switch to alternative fuels and primary energies, by fuel/source

Q137 / 150 / 163. Are you open to switching to natural gas / solar / wind in your production processes, self-generation, and back-up generation of power?						
	Natural Gas		Solar		Wind	
	%	N	%	N	%	N
Yes	63.48	73	78.26	90	40.87	47
No	36.52	42	21.74	25	59.13	68
Total	100.00	115	100.00	115	100.00	115

Ravago, Majah-Leah; Fabella, Raul; Jandoc, Karl Robert; Frias, Renzi; Magadia, J. Kathleen (2020), “Survey Data on Energy and Fuel Use of Firms in Economic Zones in the Philippines”, Data in Brief.

Table V-8. Number of firms willing to replace current fuels with alternative fuels and primary energies, by fuel, alternative fuel/source, and purpose

Q138-140 / 151-153 / 164-166. In case you decide to switch to natural gas / solar / wind in your production processes / self-generation of power / back-up power generation, which among the following fuels would you most likely replace with it?

	Production process			Self-generation of power			Back-up power generation		
	Natural Gas	Solar	Wind	Natural Gas	Solar	Wind	Natural Gas	Solar	Wind
Biodiesel	9	7	5	4	6	3	7	5	3
Bunker	2	1	1	1	1	0	1	1	0
Coal	2	4	2	3	4	3	3	4	2
Diesel	24	35	19	31	39	24	36	47	25
Gasoline	11	14	11	11	17	11	14	16	11
Kerosene	1	2	2	1	1	3	3	1	3
LPG	15	11	5	8	8	3	6	7	3
Natural gas				—	3	1	—	3	1
Propane	1	1	1	1	1	1	2	1	1
Solar				10	—	1	12	—	1
Wind				1	1	—	1	1	—
Other*	18	35	13	13	29	10	8	22	8

Note: *Argon, electricity, biomass, not applicable, none

Table V-9. Percentage and number of firms willing to switch, by energy requirement and fuel/source

Q141 / 154 / 167. In case you decide to switch to natural gas / solar / wind, what would be your requirement in mmscfd / kWh?

	Natural gas		Solar		Wind	
	%	N	%	N	%	N
1 to 20	69.86	51	41.11	37	42.55	20
21 to 40	10.96	8	15.56	14	17.02	8
41 to 60	9.59	7	8.89	8	8.51	4
61 to 80	5.48	4	5.56	5	6.38	3
81 to 100	4.11	3	3.33	3	2.13	1
101 and above	0.00	0	25.56	23	23.40	11
Total	100.00	73	100.00	90	100.00	47

Note: Response options for natural gas are in mmscfd while for solar and wind are in kWh.

Ravago, Majah-Leah; Fabella, Raul; Jandoc, Karl Robert; Frias, Renzi; Magadia, J. Kathleen (2020), "Survey Data on Energy and Fuel Use of Firms in Economic Zones in the Philippines", Data in Brief.

Table V-10. Percentage and number of firms with subsidiary/parent/partner company with experience in using alternative fuels and primary energies, by fuel/source

Q143 / 156 / 169. Does your subsidiary / parent / partner company have any experiences using natural gas / solar / wind in their production processes?

	Natural gas		Solar		Wind	
	%	N	%	N	%	N
Yes	7.25	5	20.31	13	8.93	5
No	92.75	64	79.69	51	91.07	51
Total	100.00	69	100.00	64	100.00	56

Ravago, Majah-Leah; Fabella, Raul; Jandoc, Karl Robert; Frias, Renzi; Magadia, J. Kathleen (2020), “Survey Data on Energy and Fuel Use of Firms in Economic Zones in the Philippines”, Data in Brief.

SECTION VI – Other Questions

This section covers information on business expansion considerations. This section covers Question 170 of the survey questionnaire.

Table VI-1. Number of firms by consideration in business expansion, by degree of importance

Q170. When considering a business expansion in the Philippines, what are your considerations? With 1 being the most important, rank the following.

	1	2	3	4	Mean
Cost of manpower	53	16	18	13	1.91
Cost of raw materials	31	43	15	11	2.06
Cost of electricity	16	34	46	4	2.38
Cost of fuels	0	7	21	72	3.65
Total	100	100	100	100	

Note: This question was only asked in the primary survey, hence the total number of responses is only 100.

Ravago, Majah-Leah; Fabella, Raul; Jandoc, Karl Robert; Frias, Renzi; Magadia, J. Kathleen (2020), "Survey Data on Energy and Fuel Use of Firms in Economic Zones in the Philippines", Data in Brief.
